# Purine tautomeric preferences and bond-length alternation in relation with protonation-deprotonation and alkali metal cationization

**DOI:** 10.1007/s00894-020-4343-6

**Published:** 2020-04-04

**Authors:** Ewa D. Raczyńska, Jean-François Gal, Pierre-Charles Maria, Beata Kamińska, Małgorzata Igielska, Julian Kurpiewski, Weronika Juras

**Affiliations:** 1 Department of Chemistry, Warsaw University of Life Sciences (SGGW), 02-776 Warszawa, Poland; 2 Université Côte d’Azur, CNRS, Institut de Chimie de Nice, UMR 7272, 06108 Nice, France; 3 Department of Biotechnology, Warsaw University of Life Sciences (SGGW), 02-776 Warszawa, Poland

**Keywords:** Prototropic tautomers of purine, Protonated/deprotonated forms, Adducts with Li^+^ and Na^+^, Relative stabilities in the gas phase and aqueous solution, Bond-length alternation, HOMED, H^+^ and M^+^ basicities in the gas phase

## Abstract

Quantum chemical calculations were carried out for deprotonated (**P**^**−**^) and protonated purine (**PH**^**+**^) and for adducts with one alkali metal cation (**P**^**−**^**M**^**+**^ and **PM**^**+**^, where M^+^ is Li^+^ or Na^+^) in the gas phase {B3LYP/6-311+G(d,p)}, a model of perfectly apolar environment, and for selected structures in aqueous solution {PCM(water)//B3LYP/6-311+G(d,p)}, a reference polar medium for biological studies. All potential isomers of purine derivatives were considered, the favored structures indicated, and the preferred sites for protonation/deprotonation and cationization reactions determined. Proton and metal cation basicities of purine in the gas phase were discussed and compared with those of imidazole and pyrimidine. Bond-length alternations in the **P**, **PH**^**+**^, **P**^**−**^**M**^**+**^, and **PM**^**+**^ forms were quantitatively measured using the harmonic oscillator model of electron delocalization (HOMED) indices and compared with those for **P**. Variations of the HOMED values when proceeding from the purine structural building blocks, pyrimidine and imidazole, to the bicyclic purine system were also examined. Generally, the isolated NH isomers exhibit a strongly delocalized π-system (HOMED > 0.8). Deprotonation slightly increases the HOMED values, whereas protonation and cationization change the HOMED indices in different way. For bidentate M^+^-adducts, the HOMED values are larger than 0.9 like for the largely delocalized **P**^**−**^. The HOMED values correlate well in a comprehensive relationship with the relative Gibbs energies (Δ*G*) calculated for individual isomers whatever the purine form is, neutral, protonated, or cationized. When PCM-DFT model was utilized for **P**^**−**^, **PH**^**+**^, **PM**^**+**^, and **P**^**−**^**M**^**+**^ (M^+^ = Li^+^) both electron delocalization and relative stability are different from those for the molecules in vacuo. The solvation effects cause a slight increase in HOMEDs, whereas the Δ*E*s decrease, but in different ways. Hence, contribution of particular isomers in the isomeric mixtures of **PH**^**+**^, **PM**^**+**^, and **P**^**−**^**M**^**+**^ also varies.

**Electronic supplementary material:**

The online version of this article (10.1007/s00894-020-4343-6) contains supplementary material, which is available to authorized users.

**Figure:**
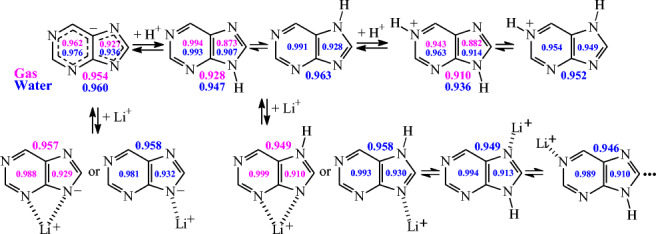
HOMED variations for the favored neutral, deprotonated, protonated, and lithiated forms of purine in the gas phase and aqueous solution

## Introduction

**Fig. 1:**
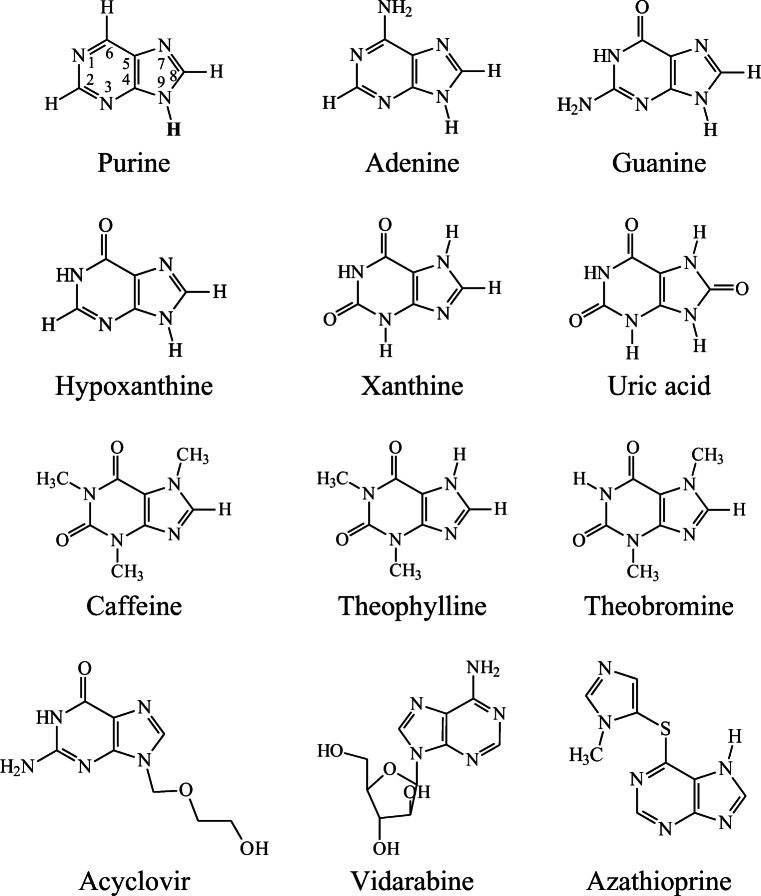
Purine and its selected biologically important derivatives

Purine (**P**, C_5_N_4_H_4_), as highlighted by its systematic name, imidazo[4,5-d]pyrimidine, is a bicyclic heterosystem constituted by the six-membered pyrimidine ring fused with the five-membered imidazole ring (Fig. 1). While not existing alone in nature, the purine moiety is the most widely distributed heterocycle in nature [1]. It is the parent compound of the wide purine family including numerous biomolecules such as nucleobases (adenine and guanine), products of their degradation (hypoxanthine, xanthine, and uric acid), and alkaloids (caffeine, theophylline, and theobromine) [2, 3]. It is also a part of various drugs which display anticancer (thioguanine and pentostatin), antitumor (vidarabine), antiviral (acyclovir, ganciclovir, and penciclovir), and immunosuppressive (azathioprine) properties [4–9]. Some purine analogs reveal the kinase inhibitory action [9, 10].

A clear vision of the structure and the fundamental physicochemical properties of purine itself, particularly its acid-base properties, seems a prerequisite for understanding the mechanisms of actions of natural products and drugs of the purine family in living organisms. In addition to the Brønsted basicity, the interactions of purine with a metal cation consider a facet of its Lewis basicity. These properties govern not only the mechanisms of various reactions, including biochemical transformations for biomolecules and drugs. They can dictate interactions of simple active molecules (e.g., inhibitors) with enzymes or receptors and also cause DNA mutation [11–17]. Moreover, various effects observed for biologically active compounds can be explained by their comparison with those for the parent system and some general conclusions derived. For these reasons, investigations on the structure and acid-base properties for unsubstituted purine are pivotal for chemistry, biochemistry, medicinal chemistry, and pharmacology of biopurines, as well as for molecular biology and genetics.

Purine, a bicyclic molecule, exhibits prototropic tautomerism, analogously to other heterocycles [18, 19]. It contains nine conjugated sites, four nitrogens, and five carbons, between which one labile proton (indicated in bold in Fig. 1) can be transferred. Nine prototropic tautomers are thus possible for purine, four NH tautomers with the labile proton at N atom (N1H, N3H, N7H, or N9H) and five CH tautomers with the labile proton at C atom (C2H, C4H, C5H, C6H, or C8H) [20]. Their amounts in the tautomeric mixture strongly depend on environment. For example, the crystal form of neutral purine favors the N7H isomer [21]. This form has also been found for solid purine when inelastic neutron scattering (INS) and Raman spectroscopy have been employed [22]. However, two isomers, N7H and N9H, have been detected in solid purine using various IR and Raman techniques [23, 24]. The coexistence of N7H and N9H tautomers in polar solvents has been observed in UV [25], NMR [26–29], IR and Raman spectra [30, 31], and at low temperature in argon matrix IR spectra [32]. Their preference has also been proved by quantum chemical methods [33–39]. In the gas phase, the N9H isomer is favored for neutral purine as confirmed on the basis of numerous theoretical and experimental studies [30, 36–47]. One-electron loss and one-electron gain change the composition of the tautomeric mixture of purine when proceeding from the gas phase to aqueous solution [20, 46, 47]. For purine radical cation, the N9H isomer seems to be favored in the gas phase, while the N1H form predominates in aqueous solution. The C6H and C8H forms are favored for radical anion in the gas phase, while the N3H isomer predominates in aqueous solution. All these variations of the tautomeric preferences for purine show clearly how sensitive is the structure of this compound on environment and electron-transfer reactions. It can also be sensitive on other factors such as presence of acid(s) or base(s), cation(s) or anion(s), substituent(s), UV, γ-, X-ray, etc.

In this paper, we report the study of the deprotonation reaction of purine (**P** – H^+^ → **P**^**−**^) that is possible in the presence of a strong base. This reaction gives the possibility to estimate the basicity of each atom in **P**^**−**^ and to confirm quantitatively the basicity of this atom that favorably binds the labile proton and dictates the tautomeric preference in neutral purine. Protonation and metal cationization play an important role in the biochemical transformations [11–13, 16]. Therefore, we also concentrated our attention on two additional factors: presence of a proton-donor acid that can protonate purine (**P** + H^+^ → **PH**^**+**^) and presence of alkali metal cation that can form an adduct with neutral purine (**P** + M^+^ → **PM**^**+**^) or in particular cases with the purine monoanion (**P −** + M^+^ → **P**^**−**^**M**^**+**^). Since the five CH tautomers can be neglected in the tautomeric mixture of neutral purine [20, 46, 47], only NH tautomers have been considered in protonation and cationization reactions. In protonation reactions, purine and its monoanion play the role of Brønsted-Lowry bases that can attach one proton, whereas in cationization reactions, purine and its monoanion behave as Lewis bases that can form adducts with one metal cation. Both reactions, protonation and cationization, can be studied for purine NH tautomers in the gas phase and aqueous solution using quantum chemical methods. The effects of proton and metal cation on tautomeric preferences and bond-length alternation are discussed here, and relations with the energetic stabilities of individual forms, their geometry-based indices of electron delocalization, and H^+^ and M^+^ basicities are examined and quantitatively described.

## Methods

For investigations in two extreme environments, the gas phase as a model of apolar medium and aqueous solution that represents a highly polar medium, typical of the biological environment, we chose the same levels of theory, B3LYP/6-311+G(d,p) [48–51] and PCM(water)//B3LYP/6-311+G(d,p) [52, 53], respectively, as previously described for neutral and ionized purine [20, 46, 47]. These levels represent a good compromise between efficiency and computing costs. The computed information is complementary to the experimental results, which only pertain to major isomers [21–31, 42, 44, 45]. For the two structural purine building blocks (imidazole and pyrimidine), analogous acid-base reactions were also investigated and compared to those for purine. Effects of rings fusion on basicity and aromaticity were analyzed for neutral, deprotonated, protonated, and cationized forms. For complexes with M^+^, we chose the two smallest alkali metal cations, Li^+^ and Na^+^, effects of which on human organisms, particularly on central nervous and cardiovascular systems and on metabolic functions and nucleic acids chemistry, have been discussed in the literature [54–58]. Their noncovalent interactions with some amino acids, peptides, sugars, and nucleobases have also been signaled [59–63].

The Gaussian 03 series of programs [64] were employed for all quantum chemical calculations. Using the B3LYP/6-311+G(d,p) level of theory [48–51], geometries of deprotonated, protonated, lithiated, and sodiated purine isomers and their structural building blocks, imidazole and pyrimidine, were optimized in their ground states. Vibrational frequencies were also calculated for all derivatives using the same DFT method. Calculations confirmed that the optimized structures are energy minima with all real frequencies. The DFT-optimized structures and electronic energies are included in Table S1 in Supplementary Material (SM). Additional calculations were carried out at the G2, G2MP2, G3, and G3B3 levels of theory [65] for imidazole and pyrimidine derivatives. Their calculated enthalpies and Gibbs energies are summarized in Table S2 (SM). For deprotonated, protonated, and lithiated purine isomers, geometry optimization were additionally carried out at the PCM(water)//B3LYP/6-311+G(d,p) level [52, 53]. The structures and electronic energies are included in Table S3 (SM). Results for neutral purine at the same DFT {B3LYP/6-311+G(d,p)} and PCM {PCM(water)//B3LYP/6-311+G(d,p)} levels [20, 46, 47] were reported elsewhere.

The structural HOMED (harmonic oscillator model of electron delocalization) indices [66–68] were determined on the basis of the calculated structures for the isomeric forms of the entire purine molecule (HOMED10, ten bonds), for their imidazole (HOMED5, five bonds) and pyrimidine (HOMED6, six bonds) fragments, as well as for the monocyclic molecules of imidazole and pyrimidine derivatives using Eq. (1). In this equation, *α*(CC) and *α*(CN) are the normalization constants for CC and CN bonds; *R*_o_(CC) and *R*_o_(CN) are the optimum bond lengths for completely delocalized compounds (benzene for CC bonds and 1,3,5-triazine for CN bonds); *R*_i_ is the calculated bond length in purine, imidazole, pyrimidine, and their derivatives; and *n* is the number of bonds (equal to 5, 6, or 10) taken into account for the HOMED estimation.

1 $$ \mathrm{HOMED}=1\hbox{--} \left\{\alpha \left(\mathrm{CC}\right)\Sigma {\left[{R}_{\mathrm{o}}\left(\mathrm{CC}\right)\hbox{--} {R}_{\mathrm{i}}\left(\mathrm{CC}\right)\right]}^2+\alpha \left(\mathrm{CN}\right)\Sigma {\left[{R}_{\mathrm{o}}\left(\mathrm{CN}\right)\hbox{--} {R}_{\mathrm{i}}\left(\mathrm{CN}\right)\right]}^2\right\}/n. $$

2 $$ \alpha =2{\left[{\left({R}_{\mathrm{o}}\hbox{--} {R}_{\mathrm{s}}\right)}^2+{\left({R}_{\mathrm{o}}\hbox{--} {R}_{\mathrm{d}}\right)}^2\right]}^{-1}. $$

3 $$ \alpha =\left(2i+1\right){\left[\left(i+1\right){\left({R}_{\mathrm{o}}\hbox{--} {R}_{\mathrm{s}}\right)}^2+i{\left({R}_{\mathrm{o}}\hbox{--} {R}_{\mathrm{d}}\right)}^2\right]}^{-1}. $$

The normalization α constants, obtained according to Eq. (2), were applied for the HOMED estimation of the pyrimidine fragments (6 bonds) and for the entire purine isomers (10 bonds). For the imidazole parts (5 bonds), the α constants, found from Eq. (3), were used. In Eqs. (2) and (3), *R*_s_ and *R*_d_ are the reference single and double bond lengths, respectively. The bond lengths *R*_o_, *R*_s_, and *R*_d_ for the selected reference molecules (ethane, ethene, and benzene for CC and methylamine, methylimine, and 1,3,5-triazine for CN) were calculated at the same DFT and PCM-DFT levels as *R*_i_ for purine derivatives and their structural building blocks. The α, *R*_o_, *R*_s_, and *R*_d_ values applied here are the same as those previously reported [47, 67, 68]. The choice of the same theoretical method for reference compounds and for investigated systems reduces the HOMED computational errors to minimum [67, 69]. The HOMED indices calculated for isolated isomers of purine derivatives are given in Figs. S1, S2, S3, and S4 (SM) and those for hydrated forms in Table S4 (SM).

The relative thermochemical quantities (Δ*E*_0_, Δ*H*_298_, *T*Δ*S*_298_, and Δ*G*_298_) for protonated and cationized isomers of purine were calculated at the B3LYP/6-311+G(d,p) level. The Δ*G* values include variations in the electronic energy, zero-point energy (ZPE), and thermal corrections to the energy and entropy (vibrational, rotational, and translational). The Δ*H*_298_ and Δ*G*_298_ values are listed in Table S5 (SM). The DFT-calculated thermochemical data for tautomers of neutral purine, reported previously [20, 46, 47], necessary for the estimation of the energetics of proton transfer and cationization, are also given in Table S5. The relative electronic energies (Δ*E*_0_) for selected purine forms calculated at the PCM(water)//B3LYP/6-311+G(d,p) level were compared with those found at the B3LYP/6-311+G(d,p) level in Table S6 (SM).

Proton affinities (PAs) and gas-phase basicities (GBs) [70, 71], defined as the enthalpy and Gibbs energy changes for the deprotonation reactions (**BH**^**+**^ → **B** + H^+^) at 298 K, were obtained for purine tautomers according to Eqs. (4) and (5), respectively. In these equations, base **B** and conjugate acid **BH**^**+**^ refer to neutral purine (**P**) and its monocation (**PH**^**+**^) or correspond to purine monoanion (**P**^**−**^) and its neutral form (**P**), respectively, *H*_298_(H^+^) = *H*_transl_(H^+^) + *RT* = 5/2*RT* = 6.2 kJ mol^−1^ and *G*_298_(H^+^) = *H*_298_(H^+^) − *TS*_transl_(H^+^) = − 26.3 kJ mol^−1^, where *S*_transl_(H^+^) = 108.95 J mol^−1^ K^−1^ at 298 K [72, 73]. The deprotonation reactions for purine refer to the following steps: deprotonation of neutral purine to its monoanion (**P** → **P**^**−**^ + H^+^) and deprotonation of the purine monocation to its neutral form (**PH**^**+**^ → **P** + H^+^). The PAs and GBs for each site in the purine monoanion are listed in Table S7 (SM), and those for the N sites in the purine NH tautomers are included in Table S8 (SM). The proton basicities for imidazole, its monoanion, and pyrimidine were also calculated at the B3LYP/6-311+G(d,p), G2, G2MP2, G3, and G3B3 levels in similar way, using Eqs. (4) and (5) for the PA and GB estimations, respectively.

4 $$ \mathrm{PA}\left(\mathbf{B}\right)={H}_{298}\left(\mathbf{B}\right)+{H}_{298}\left({\mathrm{H}}^{+}\right)-{H}_{298}\left({\mathbf{BH}}^{+}\right). $$

5 $$ \mathrm{GB}\left(\mathbf{B}\right)={G}_{298}\left(\mathbf{B}\right)+{G}_{298}\left({\mathrm{H}}^{+}\right)-{G}_{298}\left({\mathbf{BH}}^{+}\right). $$

Metal cation affinities (MCAs) and metal cation basicities (MCBs) for neutral purine **P** and for the purine monoanion **P**^**−**^, in the gas phase at 298 K, were calculated using the general Eqs. (6) and (7), respectively [74–77], derived for reaction (**BM**^**+**^ → **B** + **M**^**+**^**)** in which **B** stands here for **P** or **P**^**−**^. For individual sites, lithium cation basicity data are given in Tables S9 and S10 (SM) and sodium cation basicity data are listed in Tables S11 and S12 (SM), respectively. In Eqs. (6) and (7), *H*_298_(Li^+^) = − 19,120.35 kJ mol^−1^, *H*_298_(Na^+^) = − 425,554.67 kJ mol^−1^, *G*_298_(Li^+^) = − 19,160.02 kJ mol^−1^, and *G*_298_(Na^+^) = −425,598.75 kJ mol^−1^. The *H*_298_ and *G*_298_ values for Li^+^ and Na^+^ were calculated at the B3LYP/6-311+G(d,p) level.

6 $$ \mathrm{MCA}\left(\mathbf{B}\right)={H}_{298}\left(\mathbf{B}\right)+{H}_{298}\left({\mathrm{M}}^{+}\right)-{H}_{298}\left({\mathbf{BM}}^{+}\right). $$

7 $$ \mathrm{MCB}\left(\mathbf{B}\right)={G}_{298}\left(\mathbf{B}\right)+{G}_{298}\left({\mathrm{M}}^{+}\right)-{G}_{298}\left({\mathbf{BM}}^{+}\right). $$

For purine structural moieties (imidazole and pyrimidine), metal cation basicities were estimated at the B3LYP/6-311+G(d,p), G2, G2MP2, G3, and G3B3 levels using Eqs. (6) and (7) for the MCA and MCB, respectively. The enthalpy (*H*_298_) and Gibbs energy (*G*_298_) for metal cations at the G*n* levels are as follows (all in kJ mol^−1^): for Li^+^ − 18,991.50 and − 19,031.16 (G2 and G2MP2) and − 19,072.76 and − 19,112.43 (G3 and G3B3) and for Na^+^ −424,443.35 and − 424,487.42 (G2 and G2MP2) and − 425,104.83 and − 425,148.91 (G3 and G3B3), respectively. According to the literature [74–77], no correction for basis set superposition error (BSSE) was applied here. Theoretical estimations of Brønsted and Lewis basicities in aqueous solution are beyond the scope of this article and will be a subject of future works.

## Results and discussion

### Proton-transfer equilibria

It is well recognized that tautomeric systems exhibit amphiprotic properties [18, 19]. Depending on environment (basic or acidic), they can lose or attach a proton. Purine (Fig. 1), in fact its imidazole part and imidazole itself, contains one labile proton at the amino nitrogen atom, and thus, they display various types of prototropic tautomerism. Consequently, their tautomeric mixtures, consisting of nine and five tautomers, respectively [20, 46, 47, 78], behave like acids in the presence of bases or like bases in the presence of acids. Amino NH group in NH tautomers or CH group in CH tautomers can lose a proton in deprotonation reaction, while one of N or C atoms can attach a proton in protonation reaction. On the other hand, the pyrimidinic part of purine behaves as a nitrogen base. Its structure changes in acidic media, in which one of imino N atoms binds a proton in protonation reaction.

Protonation of C atoms in nitrogen containing heterocycles can be neglected in acid-base equilibria [18, 79, 80]. Nevertheless, it can be considered in mechanism of particular processes, e.g., in electrophilic reactions [81]. On the other hand, CH tautomers of neutral purine and imidazole possess exceptionally high energies [20, 46, 47, 78, 82], which decrease only in special conditions, e.g., during negative ionization [20, 46, 47, 78]. For simple proton-transfer reactions in the gas phase, CH tautomers can be neglected [18, 19, 71, 82, 83]. For these reasons, particular attention is paid to NH tautomers in the present work. Note that purine and imidazole tautomers contain the push-pull amidine group (–NH–CH=N– ↔ –NH^+^ = CH–N–) [71]. In this group, N(sp^3^)H is an acidic site and can lose a proton, whereas N(sp^2^) is a basic site and can attach a proton or a metal cation [71, 77].

**Scheme 1:**
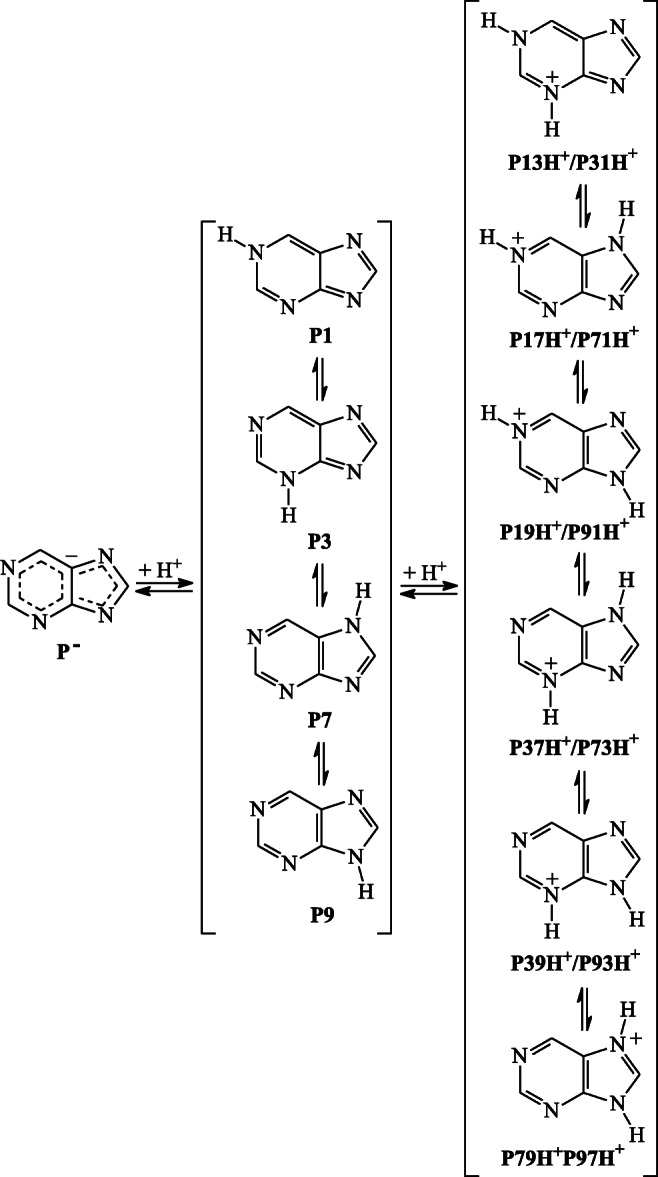
Proton-transfer equilibria possible in the gas phase for purine NH tautomers

Deprotonation of neutral purine, existing principally under the form of four most abundant tautomers of different stabilities {N1H (**P1**), N3H (**P3**), N7H (**P7**), and N9H (**P9**)}, and deprotonation of imidazole, represented essentially by two NH tautomers of equal importance {N1H (**Im1**) and N3H (**Im3**)}, lead to largely electron-delocalized monoanionic forms, **P**^**−**^ (Scheme 1) and **Im**^**−**^ (Scheme S1 in SM), respectively. On the other hand, protonation of the four purine NH tautomers at potential basic N sites gives six conjugate acid isomers, N1HN3H^+^ (**P13H**^**+**^/**P31H**^**+**^ formed from **P1** or **P3**), N1HN7H^+^ (**P17H**^**+**^/**P71H**^**+**^ formed from **P1** or **P7**), N1HN9H^+^ (**P19H**^**+**^/**P91H**^**+**^ formed from **P1** or **P9**), N3HN7H^+^ (**P37H**^**+**^/**P73H**^**+**^ formed from **P3** or **P7**), N3HN9H^+^ (**P39H**^**+**^/**P93H**^**+**^ formed from **P3** and **P9**), and N7HN9H^+^ (**P79H**^**+**^/**P97H**^**+**^ formed from **P7** or **P9**), whereas only one monocation for imidazole, N1HN3H^+^ (**ImH**^**+**^ formed from **Im1** or **Im3**). For neutral pyrimidine, two conjugate acid isomers of equal stabilities are possible, **Pym1H**^**+**^ and **Pym3H**^**+**^ (Scheme S1 in SM). They are protonated at N1 and N3 atoms, respectively.

DFT calculations carried out for all purine isomers given in Scheme 1 and additionally for isomers of imidazole and pyrimidine (Scheme S1 in SM), for which NH isomers have identical structures, confirmed that for neutral purine (Fig. S1 in SM), the **P9** tautomer is favored, and the **P7** form is a non-favored isomer in the gas phase [20, 36–47, 83, 84]. At the DFT level, their percentage contents are as follows: 99.8 and 0.2%, respectively [47]. Other NH tautomers (**P1** and **P3**) are highly unfavorable forms (< 0.01%). For protonated purine, the **P19H**^**+**^**/P91H**^**+**^ isomer, protonated at the N1 atom in the major **P9** tautomer, has the smallest Gibbs energy at the DFT level (Table S5 in SM). This indicates that protonation does not change the tautomeric preferences in purine. The other isomer, **P37H**^**+**^**/P37H**^**+**^ protonated at the N3 atom in the minor **P7** tautomer, possesses higher Gibbs energy than **P19H**^**+**^**/P91H**^**+**^ by 12.9 kJ mol^−1^. The percentage contents of **P19H**^**+**^**/P91H**^**+**^ and **P37H**^**+**^**/P37H**^**+**^ in the isomeric mixture of the purine monocation (99.5 and 0.5%, respectively) indicate that they can be considered as major and minor isomers, respectively. The four remaining isomers, **P13H**^**+**^**/P31H**^**+**^, **P17H**^**+**^**/P71H**^**+**^, **P39H**^**+**^**/P93H**^**+**^, and **P79H**^**+**^**/P97H**^**+**^, are highly unfavorable forms (Δ*G* ≥ 20 kJ mol^−1^), and they can be neglected in the isomeric mixture of **PH**^**+**^. Hence, two neutral tautomers, **P9** and **P7**, and two monocation isomers, **P19H**^**+**^**/P91H**^**+**^ and **P37H**^**+**^**/P73H**^**+**^, should be considered in monoprotonation reaction of purine for proton basicity estimation. These isomers can dictate basicity of neutral purine. Analogous conclusions have been derived investigating H-bonding reactions with HF for purine NH tautomers in the gas phase [83].

In aqueous solution (PCM-DFT), relative stabilities for neutral and protonated isomers of purine change (Table S6 in SM). As we confirmed previously [47], two tautomers, **P7** and **P9** of almost equal amount (Δ*E*_0_ = 1.0 kJ mol^−1^), predominate in the tautomeric mixture of neutral purine with slight preference of **P9**. Their high stabilities influence the composition of the isomeric mixture for protonated purine. Consequently, two isomers **P91H**^**+**^/**P19H**^+^ and **P71H**^**+**^/**P17H**^+^ (Δ*E*_0_ = 2.3 kJ mol^−1^), both protonated at the N1 atom, are favored for protonated purine with preference of **P91H**^**+**^/**P19H**^+^. This PCM-DFT observation is consistent with an experimental ^15^N NMR study carried out in aqueous solution by Gonnella and Roberts for neutral and protonated purine and also for neutral and protonated 7- and 9-methyl purine derivatives [28].

### Proton basicities

Acid-base properties of tautomeric systems are usually dictated by the abundance of each tautomer in the mixture consisting of major, minor, and negligible tautomers [18, 19]. When abundances of exceptionally unfavorable isomers are too low (< 0.01%), they have no effect on acid-base quantities. The labile proton preferentially binds to the site that possesses the strongest basicity in the anion. This means that less basic and less acidic tautomer predominates in the tautomeric mixture [18, 19]. Our DFT calculations confirm this general rule. For deprotonated purine (**P**^**−**^), N9 atom displays the strongest basicity in the gas phase (PA = 1389.4 and GB = 1358.2 kJ mol^−1^). This quantity corresponds to the microscopic basicity of a particular site in one isomer (**P9** → **P**^**−**^ + H^+^). The N9 atom binds a proton in **P**^**−**^, and **P9** becomes the weakest acid among other neutral purine tautomers (Table S7 in SM). Note that in the gas phase, the proton basicity of a base is equal to the proton acidity of its conjugate acid. Considering the major and minor isomers in the tautomeric mixture of neutral purine (**P9** and **P7**) and their percentage contents, we can predict the so-called macroscopic basicity for the purine monoanion (PA = 1389.4 kJ mol^−1^ and GB = 1358.2 kJ mol^−1^ in Table 1), which refers to the deprotonation reaction of the neutral-purine isomeric mixture {**P** (**P9** ⇌ **P7**) → **P**^**−**^ + H^+^} and which is the same as that found for the favored protonation/deprotonation N9 site in **P9**. This means that even the minor tautomer **P7** does not significantly influence the proton basicity of purine. Additionally, the PA value calculated here for N9 is not very different from that theoretically estimated earlier at the B3LYP/6-31+G(d) level (1379.9 kJ mol^−1^) [84]. It is also close to that experimentally determined by the bracketing method (1393 ± 17 kJ mol^−1^) and by the Cooks kinetic method (1389 ± 12 kJ mol^−1^) [84].

**Table 1:** Comparison of the calculated and experimental gas-phase proton basicities (PA and GB at 298 K in kJ mol^−1^) for purine, imidazole, and pyrimidine

Compound	Method	PA_calc_	GB_calc_	PA_exp_	GB_exp_
**P**^**−**^	DFT^a^	1389.4^b^	1358.2^b^	1393 ± 17^c^	
				1389 ± 12^d^	
**P**	DFT^a^	925.5^b^	893.3^b^	920.1^e^	888.2^e^
				921 ± 12^c^	
				925 ± 12^d^	
**Im**^**−**^	DFT^a^	1462.8^b^	1431.3^b^	1464.1^f^	1433.4^f^
	G2	1461.1^b^	1429.8^b^		
	G2MP2	1461.9^b^	1430.6^b^		
	G3	1464.0^b^	1432.7^b^		
	G3B3	1463.9^b^	1432.5^b^		
**Im**	DFT^a^	946.0^b^	913.7^b^	942.8^e^	909.2^e^
	G2	944.0^b^	911.7^b^		
	G2MP2	944.4^b^	912.1^b^		
	G3	945.8^b^	913.5^b^		
	G3B3	945.4^b^	913.1^b^		
**Pym**	DFT^a^	889.8^b^	858.0^b^	885.8^e^	855.7^e^
	G2	886.8^b^	855.2^b^		
	G2MP2	887.2^b^	855.5^b^		
	G3	887.4^b^	855.7^b^		
	G3B3	887.2^b^	855.4^b^		

^a^B3LYP/6-311+G(d,p). ^b^This work. ^c^Ref [84], bracketing method. ^d^Ref [84], Cooks kinetic method. ^e^Ref [70]. ^f^Ref [86]

When going from the gas phase (DFT) to aqueous solution (PCM-DFT), the structures of anionic form **P**^**−**^ and of the favored neutral tautomers **P9** and **P7** do not change significantly. Only the relative energies (Table S6 in SM) and, consequently, the amounts of **P9** and **P7** in the tautomeric mixture are different in the two environments. This difference affects the macroscopic basicity parameter (p*K*_a_) of the tautomeric mixture. Like for other tautomeric systems containing tautomers in almost equal amount [18, 19], the microscopic p*K*_a_ values of both tautomers, **P9** and **P7**, have to be taken into account when deprotonation reaction of neutral purine, **P** (**P9** ⇌ **P7**) → **P**^**−**^ + H^+^, is examined. The experimental p*K*_a_ value (8.9), cited recently by Geremia and Seybold [85] for deprotonation of neutral purine, according to our PCM studies corresponds to the tautomeric mixture (**P9** and **P7**). However, the authors did not give details on different methods of theoretical p*K*_a_ prediction, and it is not clear to which neutral form (**P9**, **P7**, or possibly their mixture) refers the calculated p*K*_a_ value (9.3) reported in their work [85]. It is not clear if they are microscopic p*K*_a_s, referring to one tautomer (**P9** or **P7**), or they are macroscopic p*K*_a_s, corresponding to the tautomeric mixture (**P9** ⇌ **P7**). More detailed investigations are needed to clarify this point.

DFT calculations performed additionally for protonation reaction of neutral purine in the gas phase (Table S8 in SM) showed that the N1 site is preferentially protonated in the major **P9** tautomer. This site (PA = 925.5 and GB = 893.3 kJ mol^−1^) has stronger basicity (by ca 30–40 kJ mol^−1^) than N3 and N7 in **P9**. PA of N1 in **P7** is only slightly lower (by 4 kJ mol^−1^). However, N3 (PA = 928.4 and GB = 896.2 kJ mol^−1^) seems to be the favored site of protonation in the minor **P7** tautomer. Low amount of **P7** (0.2%) and **P73H**^**+**^ (0.5%) in the corresponding tautomeric mixtures have no important effect on the macroscopic basicity of neutral purine (PA = 925.5 and GB = 893.3 kJ mol^−1^) which refers to the following equilibria: **PH**^**+**^ (**P91H**^**+**^/**P19H**^+^ ⇌ **P73H**^**+**^/**P37H**^+^) → **P** (**P9** ⇌ **P7**) + H^+^. Although proton basicities of N7 in **P3** (PA = 953.7 and GB = 921.9 kJ mol^−1^) and N9 in **P1** (PA = 980.2 and GB = 947.9 kJ mol^−1^) are considerably higher than those of N3 in **P7** and N1 in **P9**, they have no influence on the macroscopic basicity parameters of purine. The percentage contents of **P1** and **P3** are too low to affect the PA and GB values for the tautomeric mixture. Note that the PA value calculated here for N1 in the favored tautomer **P9** is not very different from those estimated earlier at the B3LYP/6-31+G(d) level (917.1 kJ mol^−1^) [84] and measured by different experimental techniques (920.1 [53], 921 ± 12 [84], and 925 ± 12 kJ mol^−1^ [84]).

**Chart 1:**
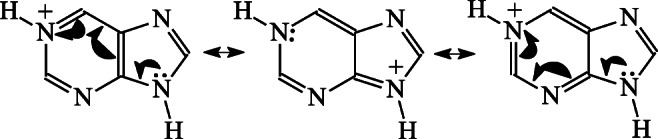
Push-pull effect in the favored purine monocation

When the proton basicity data for N1 in the favored NH tautomer **P9** are compared with those of N1 in model pyrimidine (**Pym**, PA = 889.8, GB = 858.0 kJ mol^−1^ given in Table 1), purine seems to be a considerably stronger base than the six-membered model (by ca. 35 kJ mol^−1^ at the DFT level). This means that the imidazole part in purine acts, generally, as an electron-donating (by resonance) and polarizable substituent that enhances PA and GB of N1 in **P9**. The push-pull effect in **P9** and in its protonated form (**P91H**^**+**^/**P19H**^**+**^), i.e., n-π conjugation between the imidazole amino N9 atom and the pyrimidine imino N1 atom, and polarizability of the –N=CH–NH– fragment can only explain high basicity of purine (Chart 1). Electron-accepting inductive effects of the imidazole N atoms, which decrease basicity, seem to be considerably smaller than the two other favorable effects, push-pull and polarizability, which increase PA and GB. Note that the DFT-calculated proton basicities for **Pym** are not very different from those calculated at the G*n* levels (Table 1) and also from those determined experimentally [70]. Differences in the PA and GB values found by different methods are lower than 4 kJ mol^−1^. This agreement of experimental and theoretical data confirms that the B3LYP/6-311+G(d,p) level was well chosen for PA and GB prediction of nitrogen bases [71, 87, 88].

On the other hand, purine is weaker base in the gas phase than imidazole for the neutral form as well as for its monoanion (Table 1). If we directly compare the proton basicity data of N7 in **P9** (PA = 897.9 and GB = 866.0 kJ mol^−1^, Table S5 in SM) with those of the imino N atom in **Im** (PA = 946.0 and GB = 913.7 kJ mol^−1^, Table 1), both calculated at the same DFT level, we can estimate the total unfavorable effect (ca. 50 kJ mol^−1^) of the pyrimidine –N=CH–N=CH– fragment, playing the role of a substituent at the imidazole ring (4,5-positions). This fragment acts as an electron-withdrawing group by both the flied/inductive (I) and resonance (R) effects. The polarizability (P) effect of this group seems to be weaker than the sum of I and R. In the case of the purine monoanion, the total substituent effect is considerably stronger (ca. 80 kJ mol^−1^) when the DFT-calculated PA of **Im**^**−**^ (1450.4 kJ mol^−1^, Table 1) is compared to that of **P**^**−**^ for 7-position (1373.3 kJ mol^−1^, Table S7 in SM).

The push-pull effects, i.e., n-π conjugation between the imidazole amino N9 atom and the pyrimidine imino N1 atom in **P9** and **P91H**^**+**^/**P19H**^+^ and n-π conjugation between the imidazole amino N7 atom and the pyrimidine imino N1 atom in **P7** and **P71H**^**+**^/**P17H**^+^, also explain the favored protonation sites for neutral purine in aqueous solution [28]. Our PCM-DFT calculations clearly show that the literature experimental (2.20 [28], 2.39 [89], and 2.52 [90]) p*K*_a_ values in water correspond to the following step for isomeric mixtures: **PH**^**+**^ (**P91H**^**+**^/**P19H**^+^ ⇌ **P71H**^**+**^/**P17H**^+^) → **P** (**P9** ⇌ **P7**) + H^+^. However, theoretical p*K*_a_ prediction (2.7 and 1.6) of Geremia and Seybold [85], who used different theoretical methods, is not clear. The authors did not give details on considered protonation reaction of purine. This needs further study.

### Metal cation adduct formation

**Scheme 2:**
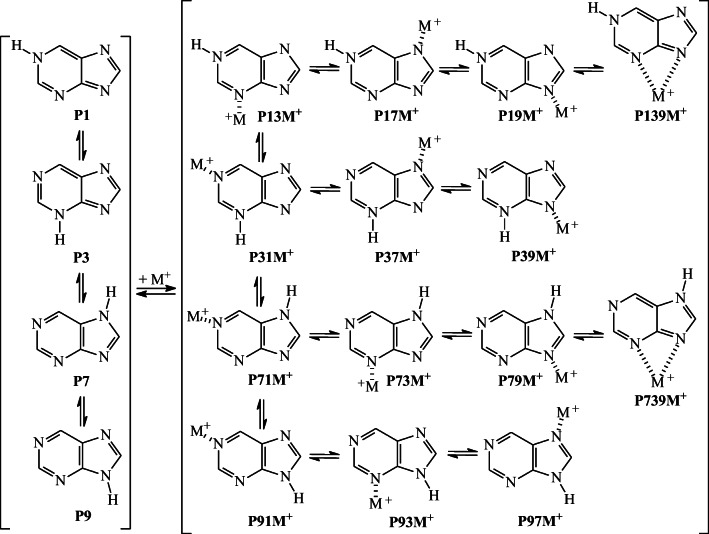
Metal cation adduct formation possible for four NH tautomers of neutral purine

The imino N1, N3, N7, and/or N9 atoms in NH tautomers of neutral purine are also potential basic sites in reaction with a metal cation (M^**+**^). One (or two) of them can bind a metal cation and form a monodentate (or bidentate) adduct. The purine C atoms can be neglected in this reaction. Scheme 2 shows fourteen possible isomers for M^**+**^-adduct that can be formed from four NH tautomers of neutral purine. Two of them are bidentate adducts, N1NN39M^+^ (**P139M**^**+**^ formed from **P1**) and N7HN39M^+^ (**P739M**^**+**^ formed from **P7**), in which a metal cation interacts with two imino N atoms (N3 and N9). The other possible twelve isomers are monodentate adducts, N1HN3M^+^, N1HN7M^+^, and N1HN9M^+^ (**P13M**^**+**^, **P17M**^**+**^, and **P19M**^**+**^, respectively, formed from **P1**); N3HN1M^+^, N3HN7M^+^, and N3HN9M^+^ (**P31M**^**+**^, **P37M**^**+**^, and **P39M**^**+**^, respectively, formed from **P3**); N7HN1M^+^, N7HN3M^+^, and N7HN9M^+^ (**P71M**^**+**^, **P73M**^**+**^, and **P79M**^**+**^, respectively, formed from **P7**); and N9HN1M^+^, N9HN3M^+^, and N9HN7M^+^ (**P91M**^**+**^, **P93M**^**+**^, and **P97M**^**+**^, respectively, formed from **P9**), in which M^+^ can only interact with one imino N atom (N3, N7, or N9 in **P1**; N1, N7, or N9 in **P3**; N1, N3, or N9 in **P7**; and N1, N3, or N7 in **P9**).

**Chart 2:**
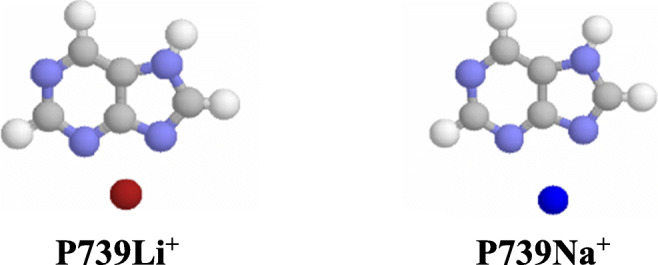
Bidentate M^+^-adducts of neutral purine favored in the gas phase

Generally, bidentate adducts have smaller Gibbs energies than monodentate ones in the gas phase [77, 91, 92]. The lowest *G* value at the DFT level (Table S5 in SM) possesses the bidentate adduct **P739M**^**+**^, formed from the neutral minor tautomer **P7** with the lithium or sodium cation (Chart 2). This adduct predominates in the isomeric **PM**^**+**^ mixture (100%). The other bidentate adduct, **P139M**^**+**^ formed from highly unfavorable tautomer **P1**, has larger *G* value than **P739M**^**+**^ (by ca. 30 kJ mol^−1^), but lower (by less than 10 kJ mol^−1^) than the monodentate adduct **P91M**^**+**^, formed from **P9** and metal cation interacting with N1. The tautomer **P9**, favored for neutral purine, can only form monodentate M^+^-adducts for which the *G* values are considerably larger than that of **P739M**^**+**^ (by 40–70 kJ mol^−1^). The differences in energetic stabilities of **P9** and **P7** and also of **P91M**^**+**^ and **P739M**^**+**^ (Table S5 in SM) clearly indicate how strong chelation effect in the bidentate M^**+**^-adduct is in apolar medium. The exceptional Gibbs energy gain (ca. 50 kJ mol^−1^) changes the tautomeric preference from **P9** for neutral purine to **P7** for the cationized derivative. High stability of bidentate metal cation adducts has also been reported for other neutral bases containing more than one N-basic sites, e.g., biguanide, metformin, imeglimin, and histamine. [77, 91, 92].

**Scheme 3:**
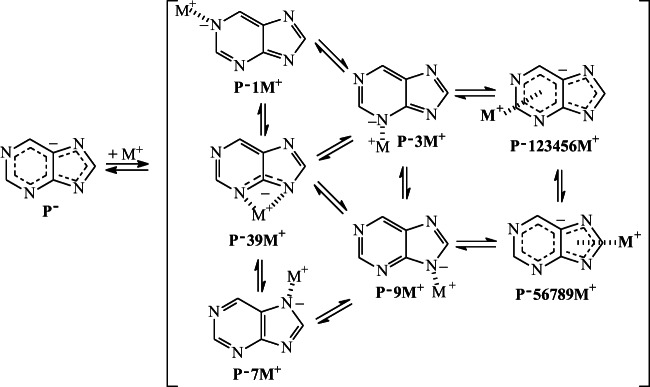
Metal cation adduct formation with the purine monoanion (structures **P**^**−**^**123456M**^**+**^ and **P**^**−**^**56789M**^**+**^ correspond to cation/π adducts)

**Chart 3:**
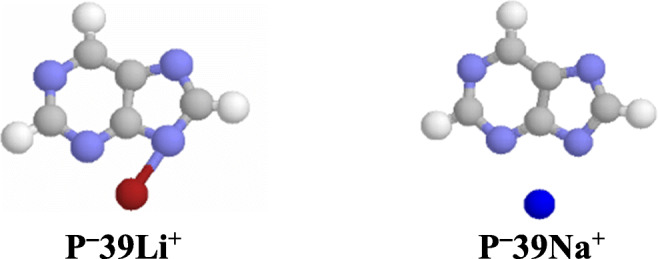
Bidentate M^+^-adducts of deprotonated purine favored in the gas phase

In the case of the purine monoanion **P**^**−**^, the N sites can also interact with a metal cation, and monodentate or bidentate adducts can be formed as in the case of neutral purine. However, lack of labile proton in **P**^**−**^ reduces the number of possible isomers for M^+^-adducts from fourteen for neutral purine (Scheme 2) to five for its monoanion (Scheme 3), one bidentate (**P**^**−**^**39M**^**+**^) and four monodentate adducts (**P**^**−**^**1M**^**+**^, **P**^**−**^**3M**^**+**^, **P**^**−**^**7M**^**+**^, and **P**^**−**^**9M**^**+**^). It also changes the character of M^+^ bonding. For neutral purine, the positive M^+^ ion interacts with N atom(s) of the neutral molecule (**M**^**+**^…**P**), while for deprotonated purine, the positive M^+^ ion interacts with N atom(s) of the negatively charged monoanion (**M**^**+**^…**P**^**−**^). This difference in interaction affects the strength of M^+^ bonding and consequently the MCA and MCB values (vide infra). Nevertheless, DFT calculations performed for metal cation adducts of the purine monoanion confirm that in the gas phase, the bidentate structure (**P**^**−**^**39M**^**+**^) has lower Gibbs energy (by more than 70 kJ mol^−1^) than the monodentate ones as it is the case for neutral purine. The bidentate form (100%) predominates in the isomeric mixture of **P**^**−**^**M**^**+**^ for Li^+^- and Na^+^-adducts (Chart 3).

**Chart 4:**
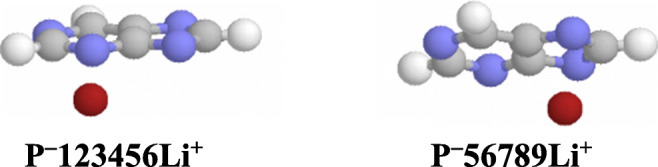
Specific interactions between Li^+^ cation and π-electrons of deprotonated purine in the gas phase

Note that the M^+^ cation can also interact with the negative charge and π-electrons delocalized in deprotonated purine (Scheme 3). Two types of Li^+^/π(−) adducts are found for the pyrimidine (**P**^**−**^**1234569Li**^**+**^) and imidazole (**P**^**−**^**56789Li**^**+**^) moieties in the gas phase (Chart 4). However, they display higher Gibbs energies than the bidentate adduct **P**^**−**^**39Li**^**+**^ (by 116 and 88 kJ mol^−1^, respectively). These specific M^+^/π(−) interactions for purine anion have not yet been described in the literature. In the case of Na^+^-adducts, analogous structures are calculated as not stable at the DFT level, and during geometry optimization, they transform to the most stable bidentate adduct **P**^**−**^**39Na**^**+**^ (Chart 3). Although interactions between M^+^ and π-electrons of neutral purine are also possible, no stable M^+^/π adducts are found for both the pyrimidine and imidazole moieties. During geometry optimization, they transform into the most stable bidentate adduct **P739M**^**+**^ (Chart 2).

**Fig. 2:**
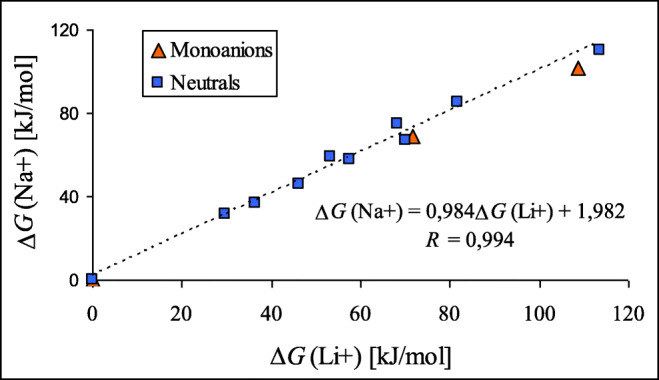
Linear tendencies between the relative Gibbs energies (Δ*G*) estimated for isomers of lithium and sodium cation adducts of neutral and deprotonated purine in the gas phase

Interestingly, the relative stabilities of lithium cation adducts in the gas phase are parallel to those of sodium cation adducts for monodentate and bidentate forms of purine monoanions analogously as it takes place for adducts of NH tautomers of neutral purine (Fig. 2). The slope of linear relationship (0.984) and the correlation coefficient (0.994) found for neutral purine are close to unity. For adducts of deprotonated purine, there are not sufficient number of data points (only three) for a good statistical analysis. Nevertheless, these points placed in Fig. 2 are close to those for neutral isomers, indicating some analogy between monodentate and bidentate Li^+^- and Na^+^-adducts for neutral and deprotonated purine.

**Chart 5:**
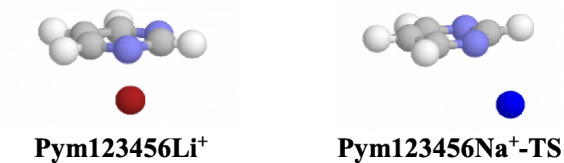
Specific Li^+^/π pyrimidine adduct and transition state of Na^+^ pyrimidine adduct in the gas phase

Analogously, M^**+**^ can interact with one imino N atom of neutral imidazole and neutral pyrimidine (Scheme S2 in SM). The possible isomers of monodentate metal cation adducts have the same structures, analogous stabilities, and identical Gibbs energies. Interactions of M^+^ cation with π-electrons of the ring are also possible (Table S1 in SM). However, a stable adduct (with all positive frequencies) exists only for Li^+^ pyrimidine (**Pym123456Li**^**+**^) at the DFT level (Chart 5). Its Gibbs energy is higher than that of the monodentate adduct **Pym1Li**^**+**^**/Pym3Li**^**+**^ by ca. 90 kJ mol^−1^, indicating that **Pym123456Li**^**+**^ is not a favored form. The N1…M^+^ and N3…M^+^ distances in **Pym123456Li**^**+**^ (2.333 and 2.334 Å) are longer than those in **Pym1Li**^**+**^**/Pym3Li**^**+**^ (1.935 Å) by ca. 0.4 Å. Analogous M^+^/π adducts have been discovered for neutral aromatic hydrocarbons and their derivatives [60, 93–98].

In the case of the Na^+^ pyrimidine adduct, the M^+^/π adduct is not stable, and a transition state (with one negative frequency) was found (**Pym123456Na**^**+**^**-TS**), for which the Na^+^ cation is not centrally located vis-à-vis the pyrimidine ring, like in **Pym123456Li**^**+**^ (Chart 5), but is moved in the NCN direction. The Gibbs energy of **Pym123456Na**^**+**^**-TS** is higher than that of the monodentate adduct (**Pym1Na**^**+**^**/Pym3Na**^**+**^) by ca. 64 kJ mol^−1^. The N1…M^+^ and N3…M^+^ distances in **Pym123456Na**^**+**^**-TS** (2.978 and 2.979 Å) are also longer than that in **Pym1Na**^**+**^**/Pym3Na**^**+**^ (2.324 Å). Their difference (0.7 Å), greater than that in **Pym123456Li**^**+**^, may explain lack of stability of the Na^+^/π adduct for pyrimidine.

**Chart 6:**
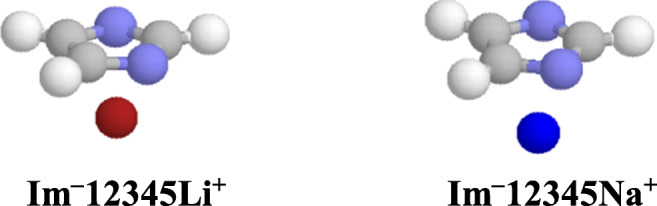
Specific M^+^/π adducts of deprotonated imidazole in the gas phase

For neutral imidazole, no additional M^+^/π adduct could be observed. The structural building block imidazole forms only monodentate M^+^-adducts (**Im13M**^**+**^**/Im31M**^**+**^). However, M^+^-adducts having a cation/π structure are found for the imidazole monoanion (**Im**^**−**^**12345M**^**+**^), where π-electrons and negative charge are delocalized on all five ring atoms which interact with M^+^ (Chart 6). The M^+^/π adducts have greater stabilities and lower *G* values (by ca. 30 kJ mol^−1^ at the G3B3 level) than the monodentate **Im13M**^**+**^**/Im31M**^**+**^ adducts with M^+^ bound to one nitrogen atom, in the plane of the cycle. Analogous conclusions on stability of monodentate and π-adducts have been derived by Elguero and co-workers [99] for monoanionic azoles (including imidazole) interacting with alkali metal cations in the gas phase at the MP2 and G2 levels.

It should be noted here that bidentate adducts are not stable in aqueous solution modeled by using the PCM procedure. Only monodentate adducts were found at the PCM(water)//B3LYP/6-311+G(d,p) level. Since some parallelism exists between the energetics of Li^+^ and Na^+^-adducts in the gas phase, we considered that calculations on series of **PLi**^**+**^ and **P**^**−**^**Li**^**+**^ isomers in aqueous solution were sufficient for giving a general picture of the solvation effects. Analogously as for protonated forms, high stabilities of the neutral purine tautomers **P9** and **P7** in aqueous solution dictate the isomeric preferences for lithium adducts (Table S6 in SM). Consequently, **P79Li**^**+**^ and **P**^**−**^**9Li**^**+**^ possess the smallest electronic energies in series of the **PLi**^**+**^ and **P**^**−**^**Li**^**+**^ isomers, respectively. For adducts of neutral purine, the two other isomers, **P97Li**^**+**^ and **P91Li**^**+**^, display very close electronic energies to that of **P79Li**^**+**^ (Δ*E*_0_ < 1 kJ mol^−1^), and the three other isomers, **P71Li**^**+**^, **P73Li**^**+**^, and **P93Li**^**+**^, have slightly higher electronic energies than the favored one (by 2.1, 3.5, and 7.2 kJ mol^−1^, respectively). However, all of them can be considered as significant in the isomeric mixture of **PLi**^**+**^ in aqueous solution. In the case of the purine anion, although the electronic energy of **P**^**−**^**7Li**^**+**^ is higher than that of **P**^**−**^**9Li**^**+**^ (by 6.7 kJ mol^−1^), both isomers play an important role in the isomeric mixture of **P**^**−**^**Li**^**+**^. The other isomers of **PLi**^**+**^ and **P**^**−**^**Li**^**+**^ (with Δ*E*_0_ > 10 kJ mol^−1^) can be neglected.

### Metal cation basicities

To our knowledge, experimental MCA and MCB data for purine have not been reported. In the literature, one can find the experimental LiCA or LiCB for imidazole (**Im**) and also MCA and MCB (M^+^ = Li^+^, Na^+^, K^+^) for pyrimidine (**Pym**), determined by different methods (equilibrium, kinetic, and threshold dissociation energy methods) [74, 76, 100, 101]. For imidazole, they have been recently revised [91]. The revised experimental LiCA (213.2 kJ mol^−1^ [91]) is close to that predicted at the G3 and G3B3 levels (211.9 and 212.0 kJ mol^−1^, respectively, see in Table 2), and it is not very different from that estimated at the DFT level (216.9 kJ mol^−1^ [91]). Unfortunately, the revised experimental LiCB (172.3 kJ mol^−1^ [91]) seems to be too low in comparison to the G3- and G3B3-calculated values. The *T*Δ*S* term (40.9 kJ mol^−1^) appears to be too high, and this problem needs additional scrutiny.

**Table 2:** Gas-phase metal cation basicities (LiCA, LiCB, NaCA, and NaCB at 298 K in kJ mol^−1^) for purine, imidazole, and pyrimidine

Compound	Method	LiCA	LiCB	NaCA	NaCB
**P**^**−**^	DFT^a^	617.5^b^	584.5^b^	529.2^b^	496.8^b^
**P**	DFT^a^	226.9^b,c^	194.3^b,c^	170.1^b,c^	138.5^b,c^
**Im**^**−**^	DFT^a^	636.1^b^	601.0^b^	527.4^b^	494.6^b^
	G2	627.0^b^	592.5^b^	520.0^b^	487.4^b^
	G2MP2	626.4^b^	592.0^b^	519.6^b^	487.1^b^
	G3	639.0^b^	604.5^b^	545.5^b^	513.0^b^
	G3B3	639.3^b^	604.5^b^	546.0^b^	513.0^b^
**Im**	DFT^a^	216.9^d^	185.9^d^	155.9^b^	126.4^b^
	G2	206.3^b^	175.9^b^	145.4^b^	116.7^b^
	G2MP2	205.9^b^	175.4^b^	145.1^b^	116.4^b^
	G3	211.9^b^	181.4^b^	157.6^b^	128.9^b^
	G3B3	212.0^b^	181.4^b^	157.5^b^	128.5^b^
	Exp	213.2^e^	172.3^e^		
**Pym**	DFT^a^	168.7^b^	138.2^b^	114.7^b^	85.7^b^
	G2	158.1^b^	128.2^b^	104.4^b^	76.3^b^
	G2MP2	157.9^b^	128.0^b^	104.4^b^	76.2^b^
	G3	162.6^b^	132.7^b^	115.5^b^	87.4^b^
	G3B3	162.8^b^	132.7^b^	115.6^b^	87.1^b^
	Exp	156.3 ± 10^f^	128.3 ± 10^f^	103.5 ± 4^f^	76.0 ± 4^f^

^a^B3LYP/6–311 + G(d,p). ^b^This work. ^c^For estimation, the percentage contents of neutral tautomers **P7** and **P9** were taken into account. ^d^Ref [91]. ^e^Revised value taken from ref. [91]. ^f^Taken from ref. [101]

For pyrimidine, the experimental LiCA and NaCA (156.3 ± 10 and 103.5 ± 4 kJ mol^−1^, respectively [101]) are close to those found at the G2 (158.1 and 104.4 kJ mol^−1^) and G2MP2 (157.9 and 104.4 kJ mol^−1^) levels (see in Table 2). They are lower than those calculated at the DFT, G3, and G3B3 levels by ca. 6–12 kJ mol^−1^. Nevertheless, difference between the experimental LiCA and NaCA (52.8 kJ mol^−1^) is close to that estimated at the DFT level (54.0 kJ mol^−1^). The same is true for the experimental and theoretical LiCB and NaCB. Difference between the experimental LiCB and NaCB (52.3 kJ mol^−1^) is almost equal to that estimated at the DFT level (52.5 kJ mol^−1^).

DFT calculations performed for NH tautomers of neutral purine (**P**) show that formation of bidentate M^+^-adducts (**P739M**^**+**^) leads to considerably higher M^+^ basicities than formation of monodentate ones (Tables S10 and S12 in SM). The main reason is that the chelation effects in bidentate M^+^-adducts seem to be stronger than the substituent effects in monodentate M^+^-adducts. Hence, the LiCA and NaCA values (226.9 and 170.1 kJ mol^−1^) as well as the LiCB and NaCB values (194.3 and 138.5 kJ mol^−1^) estimated at the DFT level for formation of bidentate M^+^-adducts from the neutral purine mixture consisting of **P7** (0.2%) and **P9** (99.8%) are larger than those for neutral pyrimidine and even larger than those for neutral imidazole (Table 2). For deprotonated purine, situation is slightly different. Formation of bidentate M^+^-adducts (**P**^**−**^**39M**^**+**^) also leads to considerably higher M^+^ basicities than formation of monodentate ones (Tables S9 and S11 in SM). However, the DFT-calculated LiCA and LiCB values (617.5 and 584.5 kJ mol^−1^) are smaller than those for deprotonated imidazole (636.1 and 601.0 kJ mol^−1^) for which Li^+^/π adduct is the favored form. Note that the minor monodentate M^+^-adduct of **Im**^**−**^ possesses considerably lower LiCA and LiCB (Table S13 in SM). The cation/π-electron effect for Na^+^ in **Im**^**−**^ adduct seems to be smaller than that for Li^+^ at the DFT level, and in this case, the Na^+^ basicity data for **P**^**−**^ are close to those for **Im**^**−**^ (NaCA 529.2 and 527.4 kJ mol^−1^ and NaCB 496.8 and 494.6 kJ mol^−1^). Nevertheless, NaCA and NaCB calculated at the G3 and G3B3 levels for Na^+^/π adduct of **Im**^**−**^ are larger than the DFT ones by ca. 20 kJ mol^−1^ (Table 2), suggesting that the imidazole monoanion is a stronger base than the purine monoanion in both formation of Li^+^- and Na^+^-adducts.

**Fig. 3:**
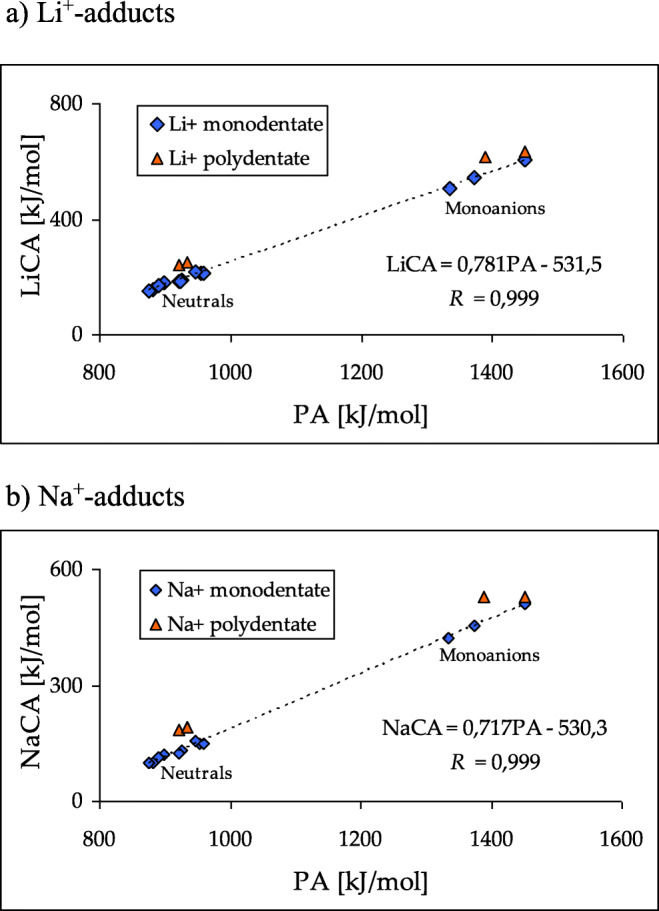
Deviations of bidentate M^+^-adducts from linear relationships between proton (PA) and metal cation basicities (LiCA and NaCA) for monodentate Li^+^- (**a**) and Na^+^-adducts (**b**) in the gas phase

Interestingly, for minor monodentate M^+^-adducts of neutral purine and for favored monodentate M^+^-adducts of neutral imidazole and pyrimidine, the structural effects observed for proton basicities are parallel to those for metal cation basicities. The same is true for monodentate M^+^-adducts of deprotonated purine and imidazole. The DFT-calculated M^+^ basicities (Tables S9, S10, S11, and S12 in SM) correlate very well with H^+^ basicities (Fig. 3) estimated for the same isomers of purine and its building blocks (Tables S7 and S8 in SM). The correlation coefficient (*R* = 0.999) is close to unity. Linear tendencies exist also between M^+^ basicities and N…M^+^ distances for monodentate adducts (Fig. S5 in SM). Stronger M^+^ basicity (larger MCA value) is related with shorter N…M^+^ distance. Some deviations of data points in Fig. 3 take place for bidentate M^+^-adducts of neutral and deprotonated purine and M^+^-cation/π-electrons adducts for deprotonated imidazole due to additional specific effects. Investigations on metal cation basicities in aqueous solution are beyond the scope of this work. In the case of alkali metal cations, this topic on purine and similar structures will be the matter of future studies.

### Bond-length alternation

First, it should be mentioned here that an application of the reformulated harmonic oscillator model of aromaticity (rHOMA) index [102], previously tested for neutral NH and CH tautomers of **P** [47], is not appropriate for analysis of bond-length alternation in heterocompounds [67, 68]. The data points for nine NH and CH tautomers of **P** are scattered between two lines, one found for compounds possessing only CC bonds and the other one obtained for derivatives containing only CN bonds (Fig. S6 in SM) [47]. The scattering of points results mainly from the use of the reference molecules of different electron delocalization in rHOMA (butadiene for CC bonds and methylamine and methylimine for CN bonds [102]). For this reason, the HOMED procedure (in which the reference compounds of similar electron delocalization were chosen) was applied here for isomers of purine derivatives and for their structural building blocks.

**Fig. 4:**
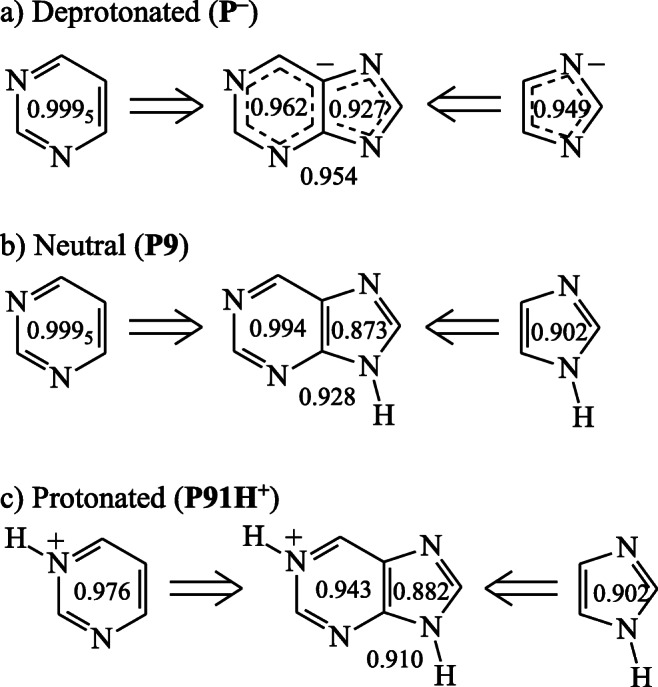
Variations of the HOMED indices estimated at the DFT level when proceeding from the two isolated constituting heterocycles to the bicyclic purine in the favored deprotonated (**a**), neutral (**b**), and protonated (**c**) forms

For the purine deprotonated form (**P**^**−**^), the negative charge, π- and n-electrons are largely delocalized as shown by the possibility to write nine reasonable resonance structures (Scheme S3 in SM) and by calculating for the DFT structure the HOMED5 (0.927), HOMED6 (0.962) and HOMED10 (0.954) values which are not very different from unity (Fig. S2 in SM). Note that for fully delocalized reference compounds, benzene and 1,3,5-triazine containing only CC and only CN bonds, respectively, HOMED = 1 [67, 68]. When the HOMED5 and HOMED6 values of the purine monoanion are compared to those of the purine structural building blocks, the imidazole monoanion and pyrimidine, structural fusion of single rings and additionally cross conjugation of n- and π-electrons in the bicyclic purine monoanion slightly reduce the partial HOMED values and, consequently, change electron delocalization in **P**^**−**^ (Fig. 4).

When the purine monoanion **P**^**−**^ is monoprotonated at one of the nine conjugated sites, nine tautomers can be formed for neutral purine (Fig. S1 in SM). Generally, monoprotonation of **P**^**−**^ reduces HOMEDs for the purine system in the gas phase as well as for the imidazole and pyrimidine fragments in higher degree for the C-protonated tautomers (which lose aromaticity) than for the N-protonated forms (which retain it), and also in higher degree in the ring containing the protonated site than in the other one (Table S7 in SM). Variations of the HOMED values for the favored **P9** tautomer when going from pyrimidine and imidazole to fused bicyclic purine (Fig. 4) show stronger effects for the imidazole than for the pyrimidine fragment. The HOMED indices decrease when going from monoanion to neutral form for the protonated part and from imidazole and pyrimidine to purine.

In the case of protonated bases, the positive charge and labile n- and π-electrons are also largely delocalized in the pyrimidine and imidazole rings and in the fused purine system **PH**^**+**^. The HOMED5, HOMED6, and HOMED10 indices estimated for the DFT structures are larger than 0.8 (Fig. S2 in SM), indicating that all the monocation isomers are aromatic. Aromaticity of the purine forms does not vary significantly when proceeding from the neutral NH tautomers (Fig. S1 in SM) to their monoprotonated forms (Fig. S2 in SM). Differences in the HOMED indices are not larger than ± 0.1 (Table S8 in SM). The lowest HOMED changes (≤ 0.005) take place for protonation at N9 in N7H and at N7 in N9H leading to **P97H**^**+**^**/P79H**^**+**^. For the favored isomer (**P91H**^**+**^), the fusion of imidazole and protonated pyrimidine, constituting the bicyclic protonated purine, reduces the HOMED values, similarly as it takes place for deprotonated and neutral purine (Fig. 4). Analogous effect has also been observed for H-bonding adducts of purine with HF in the gas phase [83].

**Fig. 5:**
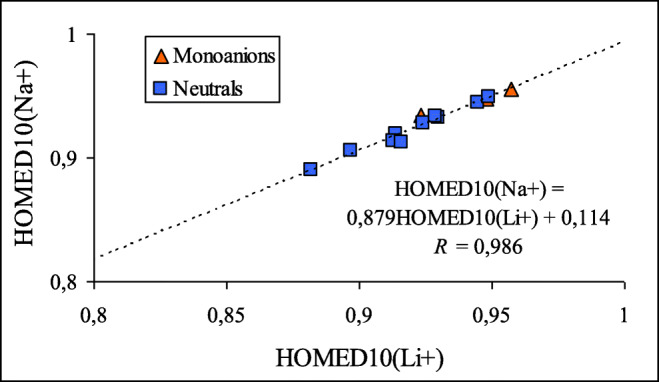
Linear tendencies between the HOMED values estimated at the DFT level for lithium and sodium cation adducts of purine NH tautomers

Interestingly, cationization of purine NH tautomers favors electron delocalization, and therefore, bond-length alternation diminishes in their bidentate M^+^-adducts (Tables S6 and S7 in SM). In the gas phase, the HOMED values increase when proceeding from neutral to cationized isomers. The same is true for the bidentate M^+^-adducts of the purine monoanion. The HOMED values are close to or even larger than 0.9 (Figs. S3 and S4 in SM). Moreover, independently on the type of adduct formation (monodentate or bidentate), the HOMED indices for lithiated isomers are parallel to those for sodiated forms. A good linear relationship (*R* = 0.986) can be observed on Fig. 5 between the HOMED10 values estimated for all ten isomers of **PLi**^**+**^ and **PNa**^**+**^. The same phenomenon takes place for M^+^-adducts of the purine monoanion. The HOMED indices for the three **P**^**−**^**Li**^**+**^ isomers are parallel to those for **P**^**−**^**Na**^**+**^. They fit well in the linear relationships found for adducts of neutral tautomers of purine.

**Fig. 6:**
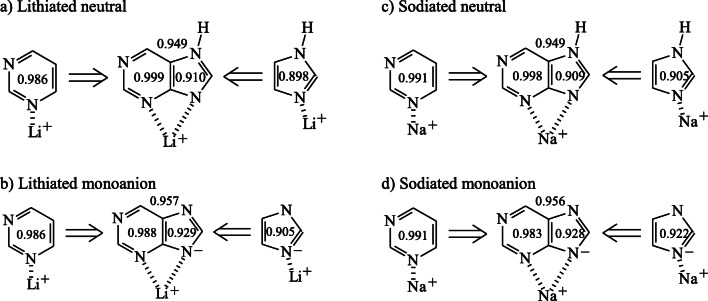
Comparison of the HOMED indices estimated at the DFT level for favored bidentate Li^+^- (**a**, **b**) and Na^+^-adducts (**c**, **d**) of neutral and deprotonated purine

When the HOMED indices estimated at the DFT level for the favored bidentate M^+^-adducts of neutral and deprotonated purine are compared with those of the corresponding cationized forms of imidazole and pyrimidine (Fig. 6), the favorable effects are almost similar. Chelation of M^+^ by two N atoms in neutral and deprotonated purine enhances delocalization of labile electrons in the bicyclic purine system in comparison to unfavorable protonation effects (Fig. 4). For the favored M^+^-adducts, the HOMED10 values for neutral purine (0.949 for both Li^+^- and Na^+^-adducts) are only slightly lower than those for the deprotonated form (0.957 and 0.956 for Li^+^- and Na^+^-adducts, respectively) and close to that for the free purine monoanion (0.954).

**Fig. 7:**
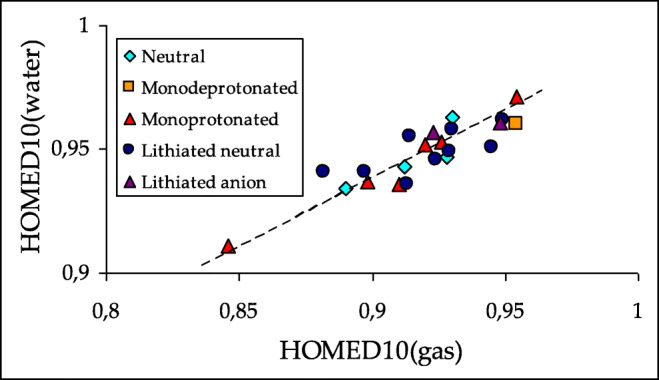
Linear trend between HOMEDs estimated in the gas phase and aqueous solution for the entire bicyclic system of neutral purine NH tautomers and their derivatives

**Fig. 8:**
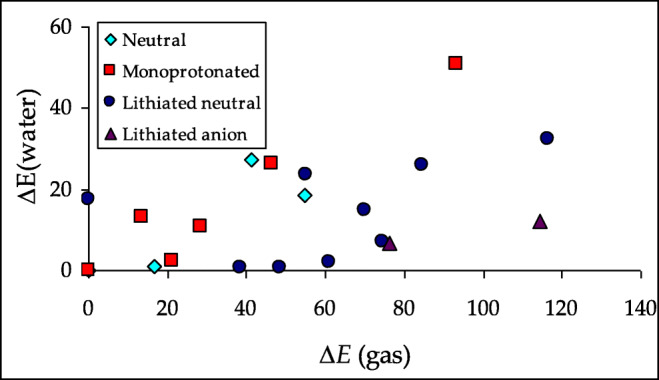
Large scatter observed in the plot of calculated relative electronic energies (∆*E* in kJ mol^−1^) in aqueous solution versus gas phase for tautomers of neutral purine and its derivatives

Generally, when proceeding from the gas phase to aqueous solution, the HOMED indices increase [47, 69, 103]. The same is true for neutral purine and its derivatives. The HOMED values for the monocyclic imidazole and pyrimidine fragments as well as for the entire bicyclic purine system are higher for PCM-DFT (Table S4 in SM) than DFT structures (Figs. S1, S2, and S3 in SM). Favorable effects take place for neutral, deprotonated, and protonated purine and for lithium cation adducts. One explanation of this phenomenon is that interactions of purine species with polar solvent change electron delocalization in the rings, and consequently, their aromatic character enhances. These effects seem to be parallel for all purine species. The HOMED values estimated in aqueous solution are parallel to those in the gas phase. The linear trend for the HOMED10 values estimated in both phases is shown in Fig. 7. On the other hand, a plot of the energetic parameters in water and in the gas phase that correspond to the relative stabilities of particular isomers presents a large scatter (Fig. 8). This means that basicities of purine N atoms strongly depend on environment and their changes are not parallel for N1, N3, N7, and N9.

**Fig. 9:**
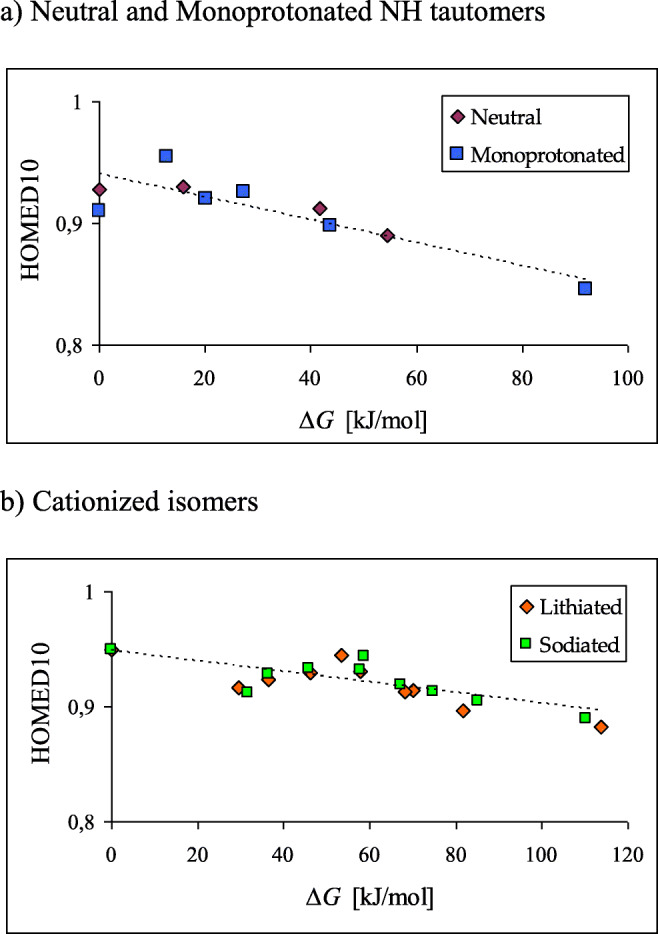
Linear trends between the HOMED10 and Δ*G* values for neutral and monoprotonated NH tautomers of purine (**a**) and for its isomers of cationized adducts (**b**) found at the DFT level

Interestingly, in the gas phase, where only structural (intramolecular) effects operate, we found recently good relations between HOMED indices that quantify bond-length alternation and relative Gibbs energies that govern tautomeric equilibria for tautomeric compounds containing CC and CN bonds [77, 91, 103]. Analogous relation takes place in the case of neutral purine (**P**). The HOMED values correlate well with the Δ*G* values for all nine NH and CH tautomers, and a linear relationship could be distinguished (see Fig. S7 in SM) [47]. The absolute value of the correlation coefficient (*R =* 0.990) is close to unity. An analogous relation between the HOMED10 and Δ*G* values exists for monoprotonated purine (**PH**^**+**^). In Fig. 9, the data points for the six NH tautomers of **PH**^**+**^ are close to those for the four neutral tautomers of **P**, and a common linear tendency exists. A linear trend exists also for isomers of cationized adducts, which seems to be common for Li^+^ and Na^+^ forms.

## Conclusions

Comparing the results of quantum chemical calculations for deprotonated (**P**^−^), protonated (**PH**^+^), and cationized (**P**M^+^) purine in the gas phase with those previously reported for neutral form (**P**) [47], all obtained with the use of the same levels of theory, leads to the following conclusions. In the gas phase, the N9 atom possesses the highest basicity in **P**^−^, a fact which is in agreement with the tautomeric preferences (N9H) in the neutral form **P**. Push-pull effect in the N9H tautomer (Chart 1) dictates the favored site of protonation (N1). On the other hand, cationization changes the tautomeric preferences in neutral purine from N9H (**P9**) to N7H (**P7**), for which a bidentate M^+^-adduct of the highest stability can be formed with N3 and N9 atoms (Chart 2). In deprotonated purine, these atoms interact the most favorably with M^+^ (Chart 3). Generally, relative stabilities of Li^+^-adducts are parallel to those of Na^+^-adducts for both **P** and **P**^−^ (Fig. 2). Lithium and sodium cation (Lewis) basicities for monodentate adducts correlate well with proton (Brőnsted) basicities, and linear relationships exist for them with correlation coefficients close to unity (Fig. 2), but bidentate M^+^-adducts deviate from these lines.

When compared with structural building blocks, in the gas phase, purine is a weaker base than imidazole but a stronger base than pyrimidine for the protonation reaction, whereas it is a stronger acid than imidazole for the deprotonation reaction (Table 1). On the other hand, purine acting as a bidentate N-ligand with M^+^ is a stronger base than the two monocyclic models that prefer to act as monodentate N-ligands.

Interactions of Li^+^ with π-electrons are observed for the purine anion in the gas phase, and two adducts are found, one for the pyrimidine fragment and the other one for the imidazole part (Chart 4). However, their energies are higher than that of the **P**^−^ bidentate (N3…Li^+^…N9) adduct. Analogously, the Li^+^/π adduct of neutral pyrimidine (Chart 5) has higher energy than its monodentate Li^+^-adduct, in which Li^+^ interacts with one N atom. Only M^+^/π adducts found for the imidazole anion for both Li^+^ and Na^+^ (Chart 6) have smaller energies than the monodentate ones (M^+^…N).

Protonation/deprotonation (Fig. 4) and cationization (Fig. 6) influence the bond-length alternation in purine when proceeding from imidazole and pyrimidine to fused purine. When this alternation is measured using the geometry-based HOMED index at the DFT level, linear relationships are found not only between the HOMED indices for Na^+^- and Li^+^-adducts (Fig. 5) but also between the HOMED indices and relative Gibbs energies for neutral, protonated, and cationized purine isomers (Fig. 9). This observation confirms linear trends observed earlier between geometric (HOMED) and energetic (Δ*G*) parameters for N-containing push-pull tautomeric compounds of biological importance such as adenine [103], metformin [91], and imeglimin [77].

When proceeding from apolar to polar medium, as modeled by gas phase and aqueous solution, the composition of the isomeric mixtures of neutral, protonated, and cationized purine changes. However, monoprotonation of **P** does not change the tautomeric preferences. The two very stable tautomers of neutral purine (**P9** and **P7** with slight preference of the first one) dictate high stability of two monocationic isomers protonated at the same favored site N1 (**P91H**^**+**^ and **P71H**^**+**^ with slight preference of the first one). On the other hand, formation of an alkali metal cation adduct enhances the stability of **P7** and slightly favors the monodentate adduct **P79Li**^**+**^. This isomer together with two other monodentate adducts of **P7** and three monodentate adducts of **P9** significantly contribute to the isomeric mixture of cationized purine. Interestingly, bidentate adducts with an alkali metal cation seem to be not stable in polar environment.

Hydration of purine isomers of neutral, deprotonated, protonated, and cationized forms enhances electron delocalization. The structural HOMED descriptors increase when going from apolar to polar environment, and linear trend is found between the HOMED values estimated at the DFT and PCM levels for all isomers of purine derivatives (Fig. 7).

## Electronic supplementary material

ESM 1 (PDF 1361 kb)

## References

[1] Rosemeyer H (2004). The chemodiversity of purine as a constituent of natural products. Chem Biodivers.

[2] Saenger W (1994). Principles of nucleic acid structure.

[3] Pozharskii AF, Soldatenkov AT, Katritzky AR (1997). Heterocycles in life and society.

[4] Cheson BD (1996). Perspectives on purine analogues. Hematol Cell Ther.

[5] Freeman S, Gardiner JM (1996). Acyclic nucleosides as antiviral compounds. Mol Biotechnol.

[6] Lamanna N, Weiss M (2004). Purine analogs in leukemia. Adv Pharmacol.

[7] Robak P, Robak T (2013). Older and new purine nucleoside analogs for patients with acute leukemias. Cancer Treat Rev.

[8] Elgemeie GH (2003). Thiopurine, mercaptopurine: their analogs and nucleosides as antimetabolites. Curr Pharm Des.

[9] Sharma S, Mehndiratta S, Kumar S, Singh J, Bedi PM, Nepali K (2015). Purine analogues as kinase inhibitors: a review. Recent Pat Anticancer Drug Discov.

[10] Sharma S, Singh J, Ojha R, Singh H, Kaur M, Bedi PMS, Nepali K (2016). Design strategies, structure activity relationship and mechanistic insights for purines as kinase inhibitors. Eur J Med Chem.

[11] Nelson DL, Cox MJ (2013) Lehninger principles of biochemistry. Freeman, New York, 6^th^ Ed

[12] Lemke TL, Williams DA, Roche VF, Zito SW (2013). Foye’s principles of medicinal chemistry (International Edition).

[13] Eichhorn GL, Marzilli LC (1981). Advances in inorganic biochemistry, metal ions in genetic information transfer.

[14] Florian J, Leszczynski J (1996). Spontaneous DNA mutations induced by proton transfer in the guanine cytosine base pairs: an energetic perspective. J Am Chem Soc.

[15] Brovarets OO, Kolomiets IM, Hovorun DM, Tada T (2012). Elementary molecular mechanisms of the spontaneous point mutations in DNA: a novel quantum-chemical insight into the classical understanding. Quantum chemistry – molecules for innovations.

[16] Cerón-Carrasco JP, Requena A, Jacquemin D (2015). Impact of DFT functionals on the predicted magnesium-DNA interaction: an ONIOM study. Theor Chem Accounts.

[17] Cerón-Carrasco JP, Jacquemin D (2015). DNA spontaneous mutation and its role in the evolution of GC-content: assessing the impact of the genetic sequence. Phys Chem Chem Phys.

[18] Elguero J, Marzin C, Katritzky AR, Linda P (1976). The tautomerism of heterocycles.

[19] Raczyńska ED, Kosińska W, Ośmiałowski B, Gawinecki R (2005). Tautomeric equilibria in relation to pi-electron delocalization. Chem Rev.

[20] Raczyńska ED, Kamińska B, Szeląg M (2009). Influence of one-electron oxidation and one-electron reduction on the tautomeric preferences for purine. Anal Bioanal Electrochem.

[21] Watson DG, Sweet RM, Marsh RE (1965). The crystal and molecular structure of purine. Acta Crystallogr.

[22] Parker SF, Jeans R, Devonshire R (2004). Inelastic neutron scattering, Raman spectroscopy and periodic DFT study of purine. Vib Spectrosc.

[23] Majoube M, Henry M, Vergoten G (1994). Vibrational spectra for purine and its 15N- and D-substituted analogues. Assignment of normal modes from ab initio 3-21G force fields. J Raman Spectrosc.

[24] Cao X, Fischer G (1999). New infrared spectra and the tautomeric studies of purine and αL-alanine with an innovative sampling technique. Spectrochim Acta.

[25] Peral F, Gallego E (2000). A study by ultraviolet spectroscopy on self association of purine, 6-methylpurine, benzimidazole, and imidazo[1,2-a]pyridine in aqueous solution. Spectrochim Acta.

[26] Chenon MT, Pugmire RJ, Grant DM, Panzica RP, Townsend LB (1975). Carbon-13 magnetic resonance. XXVI. A quantitative determination of the tautomeric populations of certain purines. J Am Chem Soc.

[27] Schumacher M, Guenther H (1982). Carbon-13 proton spin-spin coupling. 9. Purine. J Am Chem Soc.

[28] Gonella NC, Roberts JD (1982). Studies of the tautomerism of purine and the protonation of purine, and its 7- and 9-methyl derivatives, by nitrogen-15 nuclear magnetic resonance spectroscopy. J Am Chem Soc.

[29] Bartl Tomáš, Zacharová Zuzana, Sečkářová Pavlína, Kolehmainen Erkki, Marek Radek (2009). NMR Quantification of Tautomeric Populations in Biogenic Purine Bases. European Journal of Organic Chemistry.

[30] Majoube M, Millié P, Chinsky L, Turpin PY, Vergoten G (1995). Resonance Raman spectra for purine. J Mol Struct.

[31] Ten GN, Burova TG, Baranov VI (2004). Investigations of tautomeric purine forms by the methods of vibrational spectroscopy and resonance Raman scattering. I. Modeling of the purine structure in different phase states. Russ Phys J.

[32] Stepanian SG, Sheina GG, Radchenko ED, Blagoi YP (1985). Theoretical and experimental studies of adenine, purine and pyrimidine isolated molecule structure. J Mol Struct.

[33] Broo A, Holmen A (1996). Ab initio MP2 and DFT calculations of geometry and solution tautomerism of purine and some purine derivatives. Chem Phys.

[34] Borin AC, Serrano-Andrés L, Fülscher MP, Ross BO (1999). A theoretical study of the electronic spectra of N9 and N7 purine tautomers. J Phys Chem A.

[35] Burova TG, Ten GN, Kucherova VV (2004). Investigations of tautomeric purine forms by the methods of vibrational spectroscopy and resonance Raman scattering. II. Quantum-mechanical calculations of the resonance Raman scattering spectra of purine tautomers. Russ Phys J.

[36] Kwiatkowski JS, Leszczynski J (1990). Ab initio quantum-mechanical study of tautomerism of purine, adenine and guanine. J Mol Struct (THEOCHEM).

[37] Nowak MJ, Lapinski L, Kwiatkowski JS (1989). An infrared matrix isolation study of tautomerism in purine and adenine. Chem Phys Lett.

[38] Nowak MJ, Lapinski L, Kwiatkowski JS, Leszczynski J (1991). Infrared matrix isolation and ab initio quantum mechanical studies of purine and adenine. Spectrochim Acta.

[39] Nowak MJ, Rostkowska H, Lapinski L, Kwiatkowski JS, Leszczynski J (1994). Experimental matrix isolation and theoretical ab initio HF/6-31G(d,p) studies of infrared spectra of purine, adenine, and 2-chloroadenine. Spectrochim Acta.

[40] Nowak MJ, Rostkowska H, Lapinski L, Kwiatkowski JS, Leszczynski J (1994). Tautomerism N(9)H→N(7)H of purine, adenine, and 2-chloroadenine: combined experimental IR matrix isolated and ab initio quantum-mechanical studies. J Phys Chem.

[41] Houben L, Schoone K, Smets J, Adamowicz L, Maes G (1997). Combined matrix-isolation FT-IR and ab initio 6-31++G** studies on tautomeric properties of nucleic acid bases and simpler model molecules. J Mol Struct.

[42] Lin J, Yu C, Peng S, Akiyama I, Li K, Lee LK, LeBreton PR (1980). Ultraviolet photoelectron studies of the ground-state electronic structure and gas-phase tautomerism of purine and adenine. J Am Chem Soc.

[43] Kassimi NE, Thakkar AJ (1996). Polarizabilities of purine, allopurinol, hypoxanthine, xanthine and alloxanthine. J Mol Struct (THEOCHEM).

[44] Caminati W, Maccaferri G, Favero PG, Favero LB (1996). Free jet absorption milimeter wave spectrum of purine. Chem Phys Lett.

[45] Favero LB, Uriate I, Spada L, Ecija P, Calabrese C, Caminati W, Cocinero EJ (2016). Solving the tautomeric equilibrium of purine through analysis of the complex hyperfine structure of the four N-14 nuclei. J Phys Chem Lett.

[46] Raczyńska ED, Kamińska B (2010). Prototropy and π-electron delocalization for purine and its radical ions – DFT studies. J Phys Org Chem.

[47] Raczyńska ED, Kamińska B (2013). Variations of the tautomeric preferences and π-electron delocalization for the neutral and redox forms of purine when proceeding from the gas phase (DFT) to water (PCM). J Mol Model.

[48] Parr RG, Yang W (1989). Density functional theory of atoms and molecular orbital theory.

[49] Becke AD (1993). Density-functional thermochemistry. III. The role of exact exchange. J Chem Phys.

[50] Lee C, Yang W, Parr RG (1988). Development of the colle-salvetti correlation-energy formula into a functional of the electron density. Phys Rev B.

[51] Hehre WJ, Radom L, Schleyer PR, Pople JA (1986). Ab initio molecular theory.

[52] MiertusS Tomasi J (1982). Approximate evaluation of the electrostatic free energy and internal energy changes in solution processes. Chem Phys.

[53] Miertus S, Scrocco E, Tomasi J (1981). Electrostatic interaction of a solute with a continuum. Adirect utilization of ab initio molecular potentials for the prevision of solvent effects. Chem Phys.

[54] Oruch R, Elderbi MA, Khattab HA, Pryme IF, Lund A (2014). Lithium: a review of pharmacology, clinical uses, and toxity. Eur J Pharmacol.

[55] Briggs KT, Giulian GG, Li G, Kao JPY, Marino JP (2016). A molecular model for lithium’s bioactive form. Biophys J.

[56] Dudev T, Grauffel C, Lim C (2017). How native and alien metal cations bind ATP: implications for lithium as a therapeutic agent. Sci Rep.

[57] Angus M, Ruben P (2019). Voltage gated sodium channels in cancer and their potential mechanisms of action. Channels.

[58] Gonzalez-Vicente A, Saez F, Monzon CM, Asirwatham J, Garvin JL (2019). Thick ascending limb sodium transport in the pathogenesis of hypertension. Physiol Rev.

[59] Rodgers MT, Armentrout PB (2000). Noncovalent interactions of nucleic acid bases (uracil, thymine, and adenine) with alkali metal ions. Threshold collision induced dissociation and theoretical studies. J Am Chem Soc.

[60] Rodgers MT, Armentrout PB (2016). Cationic noncovalent interactions: energetics and periodic trends. Chem Rev.

[61] Rajabi K, Gillis EAL, Fridgen TD (2010). Structures of alkali metal ion-adenine complexes and hydrated complexes by IRMPD spectroscopy and electronic structure calculations. J Phys Chem A.

[62] Müller-Dethlefs K, Hobza P (2000). Noncovalent interactions: a challenge for experiment and theory. Chem Rev.

[63] Hobza P, Müller-Dethlefs K (2010). Non-covalent interaction: theory and experiment.

[64] Firsch MJ, Trucks GW, Schlegel HB, Scuseria GE, Robb MA, Cheeseman JR, Montgomery JA, Vreven T, Kudin KN, Burant JC, Millam JM, Iyengar SS, Tomasi J, Barone V, Mennucci B, Cossi M, Scalmani G, Rega N, Petersson R, Nakatsuji H, Hada M, Ehara M, Toyota K, Fukuda R, Hasegawa J, Ishida M, Nakajima T, Honda Y, Kitao O, Nakai H, Klene M, Li X, Knox JE, Hratchian HP, Cross JB, Bakken V, Adamo C, Jaramillo R, Gomperts R, Stratmann RE, Yazyev O, Austin AJ, Cammi R, Pomelli C, Ochterski JW, Ayala PY, Morokuma K, Voth GA, Salvador P, Dannenberg JJ, Zakrzewski VG, Dapprich S, Daniels AD, Strain MC, Farkas O, Malick DK, Rabuck AD, Raghavachari K, Foresman JB, Oritz JV, Cui Q, Baboul AG, Clifford S, Cioslowski J, Stefanov BB, Liu G, Liashenko A, Piskorz P, Komaromi I, Martin RL, Fox DJ, Keith T, Al-Laham MA, Peng CY, Nanayakkara A, Challacombe M, Gill PMW, Johnson B, Chen W, Wong MW, Gonzalez C, Pople JA (2004). Gaussian-03, Revision E.01.

[65] Curtiss LA, Redfern PC, Raghavachari K (2011). G*n* theory. WIREs Comput Mol Sci.

[66] Raczyńska ED, Krygowski TM, Duczmal K, Hallmann M (2006) On geometry-based HOMED (a measure of hyperconjugation, n-π, and π-π conjugation) and HOMA index (a measure of aromaticity). In: Cyrański MM, Woźniak K, Krygowski TM (eds) XVIII International Conference on Physical Organic Chemistry, ICPOC-18, August 20-25, Warsaw (p. 31–31), International Union of Pure and Applied Chemistry, http://www.science24.com/paper/8306

[67] Raczyńska ED, Hallmann M, Kolczyńska K, Stępniewski TM (2010). On the harmonic oscillator model of electron delocalization (HOMED) index and its application to heteroatomic π-electron systems. Symmetry.

[68] Raczyńska ED (2019). Application of the extended HOMED (harmonic oscillator model of aromaticity) index to simple and tautomeric five-membered heteroaromatic cycles with C, N, O, P, and S atoms. Symmetry.

[69] Raczynska ED, Juras W (2019). Effects of ionization and proton-transfer on bond length alternation in favored and rare isomers of isocytosine. Comput Theor Chem.

[70] Hunter EPL, Lias SG (1998). Evaluated gas phase basicities and proton affinities of molecules: an update. J Phys Chem Ref Data.

[71] Raczyńska ED, Gal J-F, Maria P-C (2016). Enhanced basicity of push-pull nitrogen bases in the gas phase. Chem Rev.

[72] Bartmess JE (1995). Thermodynamics of the electron and the proton. J Phys Chem.

[73] Fifen JJ, Dhaouadi Z, Nsangou M (2014). Revision of the thermodynamics of the proton in the gas phase. J Phys Chem A.

[74] Burk P, Koppel IA, Koppel I, Kurg R, Gal J-F, Maria P-C, Herreros M, Notario R, Abboud J-LM, Anvia F, Taft RW (2000). Revised and expanded scale of the gas-phase lithium-cation basicities - an experimental and theoretical study. J Phys Chem A.

[75] Duczmal K, Hallman M, Raczynska ED, Gal J-F, Maria P-C (2007). Comparison of the proton (H^+^) and alkali metal ion (Li^+^, Na^+^ and K^+^) binding affinities of pyruvate and oxamate anions in the gas phase. Quantum-chemical studies. Pol J Chem.

[76] Mayeux C, Burk P, Gal J-F, Kaljurand I, Koppel I, Leito I, Sikk L (2014). Gas-phase lithium cation basicity: revisiting the high basicity range by experiment and theory. J Am Soc Mass Spectrom.

[77] Raczynska ED, Gal J-F, Maria P-C, Fontaine-Vive F (2018). Biguanide antidiabetic drugs: imeglimin exhibits higher proton basicity but smaller lithium-cation basicity than metformin in vacuo. ACS Omega.

[78] Raczyńska ED (2012). Quantum-chemical studies of the consequences of one-electron oxidation and one-electron reduction for imidazole in the gas phase and water. Comput Theor Chem.

[79] Moet-Ner M, Liebman JF, Del Bene JE (1986). Proton affinities of azoles: experimental and theoretical studies. J Organomet Chem.

[80] Kabli S, van Beelen ESE, Ingemann S, Henriksen L, Hammerum S (2006). The proton affinities of saturated and unsaturated hetrocyclic molecules. Int J Mass Spectrom.

[81] Vianello R (2011). Protonation of azines and purines as model for the electrophilic aromatic substitution – rationalization by triadic formula. Acta Chim Slov.

[82] Alkorta I, Elguero J, Liebman JF (2006). The annular tautomerism of imidazoles and pyrazoles: the possible existence of nonaromatic forms. Struct Chem.

[83] Stasyuk OA, Szatylowicz H, Krygowski TM (2012). Effect of the H-bonding on aromaticity of purine tautomer. J Organomet Chem.

[84] Zhachkina Michelson A, Chen M, Wang K, Lee JK (2012). Gas-phase studies of purine 3-methyladenine DNA glycosylase II (AlkA) substrates. J Am Chem Soc.

[85] Geremia KL, Seybold PG (2019). Computational estimation of the acidities of purines and indoles. J Mol Model.

[86] Bartmess JE (2019) Negative ion energetics data. In NIST Chemistry WebBook, NIST Standard Reference Database No. 69, Linstrom PJ, Mallard WG (Eds) National Institute of Standards and Technology, Gaithersburg, MD, 20899; http://webbook.nist.gov/chemistry. Accessed 3 Mar 2019

[87] Maksić ZB, Kovačević B, Vianello R (2012). Advances in determining the absolute proton affinities of neutral organic molecules in the gas phase and their interpretation: a theoretical account. Chem Rev.

[88] Leito I, Koppel IA, Koppel I, Kaupmees K, Tshepelevitsh S, Saame J (2015). Basicity limits of neural organic superbases. Angew Chem Int Ed.

[89] Albert A, Brown D (1954) Purine studies. Part I. Stability to acid and alkali. Solubility. Ionization. Comparison with pteridines. J Chem Soc:2060–2071

[90] Bendich A, Russell PJ, Fox JJ (1954). The synthesis and properties of 6-chloropurine and purine. J Am Chem Soc.

[91] Raczyńska ED, Gal J-F, Maria P-C, Michalec P, Zalewski M (2017). Exceptionally high proton and lithium cation gas-phase basicity of anti-diabetic drug metformin. J Phys Chem A.

[92] Hallmann M, Raczyńska ED, Gal J-F, Maria P-C (2007). Gas-phase lithium cation basicity of histamine and its agonist 2-(β-aminoethyl)-pyridine. Experimental and theoretical studies (DFT) of chelation effect. Int J Mass Spectrom.

[93] Kumpf RA, Dougherty DA (1993). A mechanism for ion selectivity in potassium channels: computational studies of cation-pi interactions. Science.

[94] Ma JC, Dougherty DA (1997). The cation-π interaction. Chem Rev.

[95] Dougherty DA (2013). The cation-π interaction. Acc Chem Res.

[96] Gal J-F, Maria P-C, Decouzon M, Mó O, Yáñez M (2002). Gas-phase lithium-cation basicities of some benzene derivatives. An experimental and theoretical study. Int J Mass Spectrom.

[97] Mó O, Yáñez M, Gal J-F, Maria P-C, Decouzon M (2003). Enhanced Li binding energies in alkylbenzene derivatives. The scorpion effects. Eur Chem J.

[98] Gal J-F, Maria P-C, Mó O, Yáñez M, Kuck D (2006). Complexes between lithium cation and diphenylalkanes in the gas phase. The pincer effect. Chem Eur J.

[99] Blanco F, Alkorta I, Elguero J (2008). The structure of alkali metal derivatives of azoles: N-σ versus π structures. J Phys Chem A.

[100] Rodgers MT, Armentrout PB (2007). A critical evaluation of the experimental and theoretical determination of lithium cation affinities. Int J Mass Spectrom.

[101] Amunugama R, Rodgers MT (2000). Absolute alkali metal ion binding affinities of several azines determined by threshold collision-induced dissociation and ab initio theory. Int J Mass Spectrom.

[102] Krygowski TM (1993). Crystallographic studies of inter- and intramolecular interactions reflected in aromatic character of π-electron systems. J Chem Inf Comput Sci.

[103] Raczyńska ED, Makowski M, Hallmann M, Kamińska B (2015). Geometric and energetic consequences of prototropy for adenine and its structural models – a review. RSC Adv.

