# Supplementary material for: Purine tautomeric preferences and bond-length alternation in relation with protonation-deprotonation and alkali metal cationization
Source: J Mol Model. 2020 Apr 4;26(5):93. doi: 10.1007/s00894-020-4343-6 (PMC7256107; doi:10.1007/s00894-020-4343-6)
Supplement: Supplementary file 1 — (PDF 1361 kb) [file 894_2020_4343_MOESM1_ESM.pdf]

Supplementary Material

Purine tautomeric preferences and bond-length alternation in relation with protonation-deprotonation and alkali metal cationization

Ewa D. Raczyńska,[a*] Jean-François Gal,[b] Pierre-Charles Maria,[b] Beata Kamińska,[a] Małgorzata Igielska,[c] Julian Kurpiewski,[c] Weronika Juras[c]

[a]*Warsaw University of Life Sciences (SGGW), Department of Chemistry, 02-776 Warszawa, Poland*
[b] *Université Côte d'Azur, CNRS, Institut de Chimie de Nice, UMR 7272, 06108 NICE, France*
[c]*Warsaw University of Life Sciences (SGGW), Department of Biotechnology, 02-776 Warszawa, Poland*

Table of content	Page
Structures, electronic energies, and atom coordinates for deprotonated, neutral, protonated, and cationized forms of purine and its structural building blocks calculated in the gas phase at the B3LYP/6-311+G(d,p) level (Table S1**)**	S2
Enthalpies and Gibbs energies calculated for imidazole and pyrimidine derivatives at the DFT(B3LYP)/6-311+G(d,p) and G*n* levels (Table S2)	S17
Structures, electronic energies, and atom coordinates for deprotonated, neutral, protonated, and lithiated forms of purine calculated in aqueous solution at the PCM(water)//B3LYP/6-311+G(d,p) level (Table S3)	S18
PCM parameters	S26
HOMED indices for the imidazole and pyrimidine fragments, and also for the entire purine system of the deprotonated, neutral, protonated, lithiated neutral, and lithiated monoanionic forms estimated at the PCM(water)//B3LYP/6-311+G(d,p) level (Table S4)	S27
Relative enthalpies and relative Gibbs energies for isomers of purine derivatives calculated at the B3LYP/6-311+G(d,p) level (Table S5)	S27
Comparison of the relative electronic energies for isomers of purine derivatives calculated in aqueous solution at the PCM(water)//B3LYP/6-311+G(d,p)} level with those found in the gas phase at the B3LYP/6-311+G(d,p) level (Table S6)	S28
Differences in the HOMED values when proceeding from deprotonated to neutral purine, and proton basicities for individual N and C sites in purine monoanion calculated at the B3LYP/6-311+G(d,p) level (Table S7)	S28
Differences in the HOMED values when proceeding from neutral to protonated purine, and proton basicities for individual N atoms in neutral NH tautomers calculated at the B3LYP/6-311+G(d,p) level (Table S8)	S29
Differences in the HOMED values when proceeding from deprotonated purine to lithiated purine monoanion, and lithium-cation basicities for N atoms in monoanion calculated at the B3LYP/6-311+G(d,p) level (Table S9)	S29
Differences in the HOMED values when proceeding from neutral to lithiated purine, and lithium-cation basicities for N atoms in neutral NH tautomers calculated at the B3LYP/6-311+G(d,p) level (Table S10)	S30
Differences in the HOMED values when proceeding from deprotonated purine to sodiated purine monoanion, and sodium-cation basicities for N atoms in monoanion calculated at the B3LYP/6-311+G(d,p) level (Table S11)	S30
Differences in the HOMED values when proceeding from neutral to sodiated purine, and sodium-cation basicities for N atoms in neutral NH tautomers calculated at the B3LYP/6-311+G(d,p) level (Table S12)	S30

Gas-phase metal-cation basicities for the monodentate M⁺-adduct formed with the imidazole monoanion (Table S13)	S31
Nine prototropic tautomers of neutral purine, their percentage contents, and geometry-based indices estimated at the B3LYP/6-311+G(d,p) level (Fig S1)	S31
Monodeprotonated and monoprotonated forms of purine NH tautomers, their percentage contents, HOMED5, HOMED6, and HOMED10 estimated at the B3LYP/6-311+G(d,p) level (Fig S2)	S32
Lithiated isomers of purine monoanion and of its neutral NH tautomers, their percentage contents, HOMED5, HOMED6, and HOMED10 estimated at the B3LYP/6-311+G(d,p) level (Fig S3)	S33
Sodiated isomers of purine monoanion and of its neutral NH tautomers, their percentage contents, HOMED5, HOMED6, and HOMED10 estimated at the B3LYP/6-311+G(d,p) level (Fig S4)	S34
Linear trends between N…M⁺ and MCA for monodentate adducts of neutral and deprotonated forms of purine and its structural building blocks found at the B3LYP/6-311+G(d,p) level (Fig S5)	S35
Lack of correlation between rHOMA and HOMED for all nine neutral purine tautomers at the B3LYP/6-311+G(d,p) level (Fig S6)	S35
Linear trend between the HOMED indices and relative Gibbs energies estimated for all nine possible neutral purine tautomers in the gas phase at the B3LYP/6-311+G(d,p) level (Fig S7)	S36
Deprotonation/protonation reactions for purine building blocks (Scheme S1)	S36
Cationization reactions for purine building blocks (Scheme S2)	S37
Resonance structures for the purine monoanion (Scheme S3)	S37

Table S1 Structures, electronic energies (*E* in Hartree), and atom coordinates (in Å) for deprotonated, neutral [47], protonated, and cationized forms of purine and its structural building blocks calculated in the gas phase at the B3LYP/6-311+G(d,p) level

a) Deprotonated forms

Structure	Electronic energy and atom coordinates
**P⁻**<br>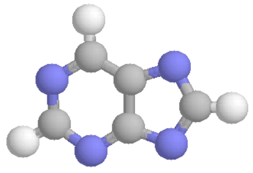	*E*: -411.515040<br>C -0.878216 -1.282011 0.000000<br>N 0.313760 -1.902280 0.000000<br>C 1.410891 -1.117219 0.000000<br>N 1.484055 0.211973 0.000000<br>C 0.286183 0.829175 0.000000<br>C -0.956229 0.103339 0.000000<br>N -1.995649 0.999937 0.000000<br>C -1.337806 2.171637 0.000000<br>N 0.017370 2.160095 0.000000<br>H -1.767947 -1.910512 0.000000<br>H 2.361276 -1.648979 0.000000<br>H -1.880257 3.112587 0.000000
**Im⁻**<br>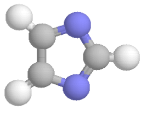	*E*: -225.714033<br>C -0.925232 -0.339362 0.000000<br>N -1.010368 1.006866 0.000000<br>N 0.319566 -0.859035 0.000000<br>C 1.112802 0.258671 0.000000<br>C 0.304974 1.392062 0.000000<br>H -1.809549 -0.969663 0.000000<br>H 2.195199 0.187629 0.000000<br>H 0.590939 2.438413 0.000000

b) Neutral isomers

**P1**<br>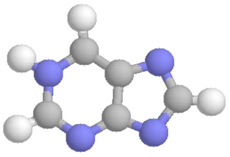	*E*: -412.034525<br>C -0.732855 -1.466148 -0.048200<br>N 0.523632 -2.010808 -0.027329<br>C 1.662381 -1.246157 0.016998<br>N 1.673741 0.054071 0.043546<br>C 0.455848 0.649462 0.024669<br>C -0.797874 -0.103778 -0.022339<br>N -1.835498 0.779293 -0.030849<br>N 0.173840 1.945360 0.043736<br>C -1.196277 1.949767 0.009040<br>H -1.576029 -2.143092 -0.083268<br>H 2.593318 -1.801911 0.029394<br>H -1.739579 2.886499 0.014194<br>H 0.624954 -3.015777 -0.045054
**P2**<br>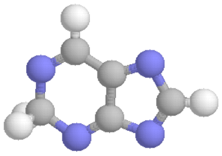	*E*: -411.985240<br>C -0.707247 -1.561090 0.023972<br>N 0.429013 -2.120859 -0.190537<br>C 1.614579 -1.309414 -0.334301<br>N 1.604060 0.136270 -0.249679<br>C 0.456600 0.649998 -0.036004<br>C -0.796727 -0.124066 0.120779<br>N -1.793268 0.677940 0.326783<br>N 0.119041 2.004093 0.095830<br>C -1.160971 1.960360 0.298837<br>H -1.594962 -2.181988 0.130868<br>H 2.069269 -1.571189 -1.300351<br>H -1.766838 2.845415 0.446861<br>H 2.343206 -1.669082 0.406148
**P3**<br>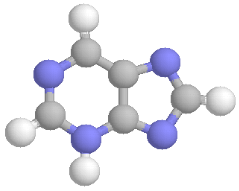	*E*: -412.039654<br>C -0.698722 -1.517480 -0.029300<br>N 0.521500 -2.122503 -0.006600<br>C 1.599226 -1.383745 0.024585<br>N 1.592261 -0.021183 0.036342<br>C 0.402394 0.637044 0.014803<br>C -0.808569 -0.146294 -0.019830<br>N -1.855650 0.737597 -0.036130<br>N 0.141504 1.927961 0.020117<br>C -1.240897 1.914317 -0.011721<br>H -1.563569 -2.171352 -0.054865<br>H 2.575427 -1.854178 0.042867<br>H -1.790180 2.846782 -0.016163<br>H 2.460423 0.502592 0.060844
**P4**<br>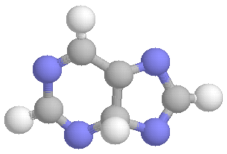	*E*: -411.969039<br>C -0.732781 -1.567053 0.161968<br>N 0.299858 -2.132264 -0.364509<br>C 1.387654 -1.304578 -0.759952<br>N 1.590704 -0.063860 -0.520833<br>C 0.597678 0.551006 0.329180<br>C -0.731869 -0.125801 0.339634<br>N -1.706224 0.720036 0.337261<br>N 0.257584 1.965011 0.176261<br>C -1.024269 1.979022 0.253980<br>H -1.607021 -2.170072 0.399565<br>H 2.129061 -1.835786 -1.349622<br>H -1.610322 2.889998 0.249663<br>H 1.016344 0.497822 1.354271

**P5**<br>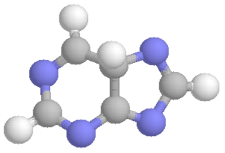	*E*: -411.979351<br>C -0.661039 -1.487385 -0.184064<br>N 0.424808 -2.083329 0.140322<br>C 1.437806 -1.306529 0.757846<br>N 1.526156 -0.021493 0.803303<br>C 0.443059 0.630220 0.254693<br>C -0.893808 -0.038900 0.167736<br>N -1.676078 0.848941 -0.682190<br>N 0.445180 1.760464 -0.373276<br>C -0.870045 1.832607 -0.898916<br>H -1.456183 -2.055416 -0.664571<br>H 2.240975 -1.895772 1.188886<br>H -1.157676 2.705281 -1.473263<br>H -1.403414 -0.066256 1.148064
**P6**<br>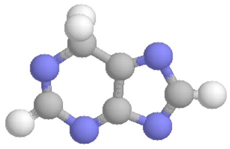	*E*: -411.981451<br>C -0.676658 -1.601323 0.006073<br>N 0.680870 -2.095443 -0.156442<br>C 1.653494 -1.270391 -0.225047<br>N 1.636606 0.151238 -0.165608<br>C 0.472160 0.663760 -0.022617<br>C -0.787681 -0.127841 0.077475<br>N -1.792260 0.667090 0.215131<br>N 0.122886 2.012436 0.068705<br>C -1.165022 1.961260 0.201808<br>H -1.100999 -2.069407 0.904742<br>H 2.654565 -1.673666 -0.346459<br>H -1.786032 2.842542 0.302692<br>H -1.282000 -1.999502 -0.819629
**P7**<br>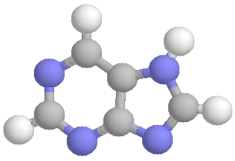	*E*: -412.049062<br>C -0.614755 -1.574005 -0.006897<br>N 0.593962 -2.134080 0.001958<br>C 1.667820 -1.319782 0.011524<br>N 1.686090 0.010494 0.013382<br>C 0.477505 0.574262 0.004514<br>C -0.713795 -0.189771 -0.005925<br>N -1.732928 0.742451 -0.012800<br>N 0.183062 1.924058 0.003919<br>C -1.122553 1.975583 -0.006260<br>H -1.477521 -2.235061 -0.014603<br>H 2.632536 -1.816624 0.018531<br>H -1.706107 2.885494 -0.009685<br>H -2.725544 0.568011 -0.021183
**P8**<br>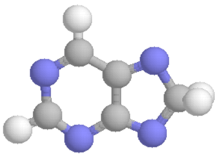	*E*: -411.991503<br>C -0.563266 -1.636935 -0.010340<br>N 0.635233 -2.120550 -0.006752<br>C 1.722931 -1.221971 0.002708<br>N 1.720138 0.071070 0.008699<br>C 0.457436 0.631968 0.005161<br>C -0.754102 -0.201634 -0.004607<br>N -1.826329 0.518499 -0.006494<br>N 0.146323 1.887148 0.009455<br>C -1.302042 1.880738 0.002420<br>H -1.411311 -2.318984 -0.017641<br>H 2.686582 -1.722301 0.005084<br>H -1.688542 2.413457 0.881492<br>H -1.680030 2.421660 -0.875343

P9

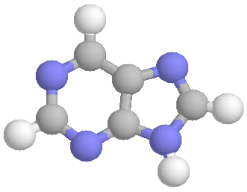

E: -412.055396

C	-0.679710	-1.556297	0.000000
N	0.514841	-2.153190	0.000000
C	1.613752	-1.380070	0.000000
N	1.673510	-0.044581	0.000000
C	0.472504	0.516849	0.000000
C	-0.761244	-0.166526	0.000000
N	-1.816449	0.732495	0.000000
N	0.137694	1.850754	0.000000
C	-1.242921	1.905003	0.000000
H	0.782380	2.626580	0.000000
H	-1.563551	-2.187401	0.000000
H	2.564542	-1.902584	0.000000
H	-1.764839	2.851306	0.000000

Im1/Im3

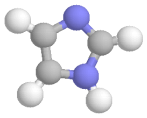

E: -226.282788

C	-2.098146	-0.192239	0.000000
C	-2.167632	1.176347	0.000000
N	-0.855390	1.603091	0.000000
C	-0.067285	0.486727	0.000000
N	-0.786360	-0.611100	0.000000
H	-2.913587	-0.898013	0.000000
H	-2.996155	1.864084	0.000000
H	-0.539455	2.559748	0.000000
H	1.010631	0.538644	0.000000

Pym

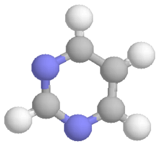

E: -264.393903

C	-1.255026	-0.202984	0.000000
N	-1.321641	1.130527	0.000000
C	-0.151233	1.775383	0.000000
C	1.066260	1.102370	0.000000
C	1.008851	-0.287574	0.000000
N	-0.150181	-0.952658	0.000000
H	-2.201590	-0.735277	0.000000
H	-0.195479	2.860992	0.000000
H	2.010188	1.633178	0.000000
H	1.913491	-0.889348	0.000000

c) Protonated isomers

$P13H^+/P31H^+$

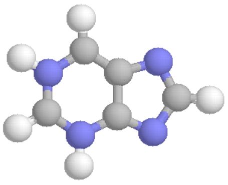

E: -412.383710

C	-1.013657	-1.305410	0.008794
N	0.294794	-1.766382	-0.008150
C	1.376969	-0.984341	-0.020202
N	1.244751	0.339576	-0.016332
C	-0.002520	0.912590	0.000049
C	-1.181631	0.043583	0.013140
N	-2.269625	0.857119	0.027923
C	-1.735680	2.069186	0.023429
N	-0.339572	2.162460	0.006346
H	-1.798783	-2.049343	0.017225
H	2.360990	-1.431119	-0.033112
H	-2.336280	2.968834	0.032352
H	0.449933	-2.769510	-0.011771
H	2.072781	0.933565	-0.025550

P17H$^+$/P71H$^+$

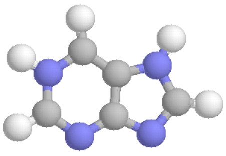

E: -412.411162

C	-1.020915	-1.357139	0.011033
N	0.269474	-1.780578	-0.007322
C	1.337788	-0.925319	-0.020875
N	1.223670	0.374073	-0.017543
C	-0.027522	0.852558	0.000336
C	-1.185995	0.001093	0.015190
N	-2.250491	0.866606	0.030935
C	-1.714138	2.128857	0.025269
N	-0.398944	2.154500	0.007251
H	-1.801048	-2.106977	0.020726
H	2.317797	-1.388313	-0.034986
H	-2.345551	3.006943	0.035150
H	0.456442	-2.778507	-0.011277
H	-3.236417	0.639582	0.044394

P19H$^+$/P91H$^+$

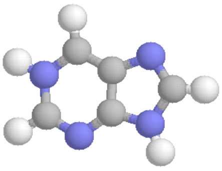

E: -412.419202

C	-0.999655	-1.293671	0.010070
N	0.279135	-1.751548	-0.007250
C	1.373208	-0.933306	-0.021957
N	1.294035	0.373776	-0.020763
C	0.050294	0.849361	-0.003921
C	-1.148408	0.072442	0.012200
N	-2.254266	0.886618	0.027173
C	-1.764488	2.094940	0.020719
N	-0.373752	2.134222	0.001981
H	-1.807188	-2.014196	0.020906
H	2.340602	-1.420673	-0.035065
H	-2.349449	3.004064	0.028713
H	0.438430	-2.754658	-0.009492
H	0.213322	2.960219	-0.005854

P37H$^+$/P73H$^+$

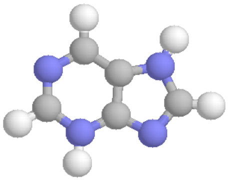

E: -412.414001

C	-0.949241	-1.343875	0.010739
N	0.305247	-1.827822	-0.006306
C	1.327682	-1.003240	-0.019488
N	1.202799	0.348129	-0.017035
C	-0.033210	0.894792	-0.000175
C	-1.149569	0.026145	0.014277
N	-2.222172	0.894096	0.029096
C	-1.706663	2.156396	0.022916
N	-0.382832	2.191257	0.005186
H	-1.755514	-2.070136	0.021069
H	2.336766	-1.397948	-0.033218
H	-2.338916	3.033096	0.031858
H	2.023939	0.950873	-0.027735
H	-3.205017	0.652566	0.042295

P39H$^+$/P93H$^+$

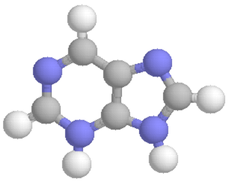

E: -412.401536

C	-0.941383	-1.272051	0.011952
N	0.295214	-1.807852	-0.004795
C	1.344419	-1.028628	-0.020002
N	1.275779	0.338026	-0.020136
C	0.055480	0.908872	-0.003658
C	-1.109011	0.105854	0.013175
N	-2.224911	0.914545	0.028083
C	-1.765885	2.126859	0.020689
N	-0.363459	2.190836	0.001160
H	-1.783015	-1.956479	0.024224
H	2.341449	-1.453602	-0.033525
H	-2.357838	3.031100	0.028210
H	2.134880	0.882172	-0.032537
H	0.193613	3.036437	-0.007430

**P79H$^+$/P97H$^+$**<br>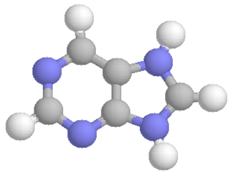	*E*: -412.408366<br>C -0.945260 -1.344749 0.015799<br>N 0.290124 -1.823251 -0.002180<br>C 1.333422 -0.970772 -0.019574<br>N 1.278569 0.364296 -0.021058<br>C 0.045015 0.822430 -0.003094<br>C -1.114310 0.042714 0.015918<br>N -2.185323 0.936071 0.030605<br>C -1.710323 2.182478 0.021218<br>N -0.376192 2.152718 0.001025<br>H -1.769942 -2.050901 0.029576<br>H 2.320316 -1.418264 -0.033760<br>H -2.314191 3.077152 0.028833<br>H -3.171618 0.703169 0.046002<br>H 0.233893 2.963689 -0.009650
**ImH$^+$**<br>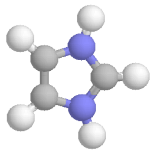	*E*: -226.654842<br>C -1.662195 -0.642468 0.000000<br>N -1.647303 0.691512 0.000000<br>C -0.342452 1.149546 0.000000<br>C 0.453901 0.046834 0.000000<br>N -0.391202 -1.047819 0.000000<br>H -0.098789 -2.017746 0.000000<br>H -2.476076 1.274086 0.000000<br>H -0.097259 2.197889 0.000000<br>H 1.526162 -0.050069 0.000000<br>H -2.536016 -1.273515 0.000000
**Pym1H$^+$/Pym3H$^+$**<br>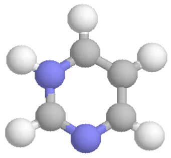	*E*: -264.744074<br>C -1.275660 -0.248930 0.000000<br>N -1.277367 1.105467 0.000000<br>C -0.125308 1.813474 0.000000<br>C 1.064477 1.118042 0.000000<br>C 0.994021 -0.279264 0.000000<br>N -0.167394 -0.948485 0.000000<br>H -2.239433 -0.746519 0.000000<br>H -0.205281 2.893028 0.000000<br>H 2.011135 1.641635 0.000000<br>H 1.894881 -0.884540 0.000000<br>H -2.168761 1.596821 0.000000

d) Lithiated neutral isomers

**P17Li$^+$**<br>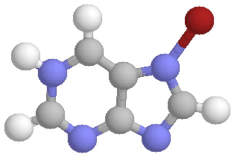	*E*: -419.401633<br>C -1.039288 -1.331367 0.010277<br>N 0.248825 -1.774611 -0.007981<br>C 1.326461 -0.929885 -0.022178<br>N 1.228460 0.368432 -0.019666<br>C -0.023732 0.860444 -0.001836<br>C -1.208512 0.026732 0.013834<br>N -2.303746 0.861572 0.029791<br>C -1.720156 2.094459 0.022809<br>N -0.387425 2.151186 0.004228<br>H -1.818110 -2.082792 0.020422<br>H 2.299980 -1.406183 -0.036120<br>H -2.316129 2.999867 0.031854<br>H 0.425530 -2.772378 -0.011318<br>Li -4.178357 0.691384 0.055615

**P139Li^{+}**<br>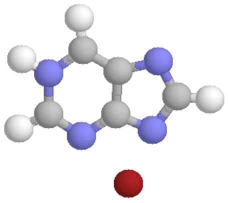	*E*: -419.416934<br>C -1.038341 -1.236662 0.007735<br>N 0.277884 -1.620318 -0.008769<br>C 1.330867 -0.754509 -0.020274<br>N 1.175017 0.548110 -0.016478<br>C -0.110185 0.961182 -0.000230<br>C -1.270134 0.112255 0.012557<br>N -2.378114 0.910043 0.027246<br>C -1.872296 2.133236 0.022654<br>N -0.490126 2.237470 0.006331<br>H -1.789113 -2.015866 0.015701<br>H 2.320000 -1.193594 -0.032960<br>H -2.494696 3.017327 0.031488<br>H 0.489542 -2.611708 -0.012791<br>Li 1.379835 2.757456 -0.015509
**P31Li^{+}**<br>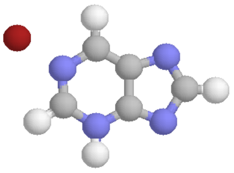	*E*: -419.383926<br>C -1.041351 -1.252312 0.004513<br>N 0.230946 -1.794178 -0.011268<br>C 1.281297 -0.985392 -0.020249<br>N 1.187354 0.358077 -0.014820<br>C -0.039127 0.955120 0.000713<br>C -1.216610 0.101440 0.010831<br>N -2.303003 0.922199 0.025006<br>C -1.755748 2.131598 0.023602<br>N -0.366670 2.216148 0.008863<br>H -1.878899 -1.941895 0.011282<br>H 2.282496 -1.396558 -0.032628<br>H -2.352109 3.034009 0.032965<br>H 2.027444 0.931318 -0.022130<br>Li 0.311340 -3.721134 -0.018209
**P37Li^{+}**<br>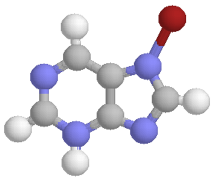	*E*: -419.407385<br>C -1.044801 -1.255440 0.009706<br>N 0.198410 -1.781808 -0.005658<br>C 1.238465 -0.984148 -0.017119<br>N 1.147489 0.371178 -0.014498<br>C -0.076726 0.951166 0.000689<br>C -1.230274 0.113384 0.013419<br>N -2.321888 0.964855 0.027298<br>C -1.745352 2.191808 0.021863<br>N -0.401226 2.241614 0.006099<br>H -1.862072 -1.970707 0.018584<br>H 2.237949 -1.402130 -0.029435<br>H -2.332632 3.101454 0.029889<br>H 1.983049 0.950881 -0.023727<br>Li -4.185251 0.655652 0.048680
**P39Li^{+}**<br>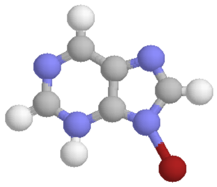	*E*: -419.396102<br>C -1.070587 -1.199239 0.005487<br>N 0.167376 -1.742267 -0.006539<br>C 1.211956 -0.959019 -0.014455<br>N 1.142306 0.406081 -0.011397<br>C -0.075909 1.002603 0.000548<br>C -1.235665 0.173513 0.009439<br>N -2.342861 0.986020 0.020860<br>C -1.836782 2.190608 0.018502<br>N -0.435121 2.295448 0.005999<br>H -1.911704 -1.883868 0.011817<br>H 2.209285 -1.383165 -0.024148<br>H -2.456193 3.079736 0.025656<br>H 2.003784 0.941617 -0.018158<br>Li 0.265466 4.050873 0.002747

P71Li$^+$

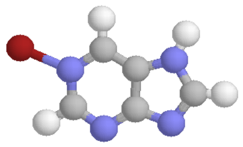

E:	-419.405104		
C	-1.096838	-1.294990	0.004671
N	0.176033	-1.753885	-0.008738
C	1.186373	-0.838992	-0.016292
N	1.079326	0.472791	-0.012090
C	-0.172772	0.935516	0.001093
C	-1.303645	0.066178	0.009944
N	-2.390194	0.908886	0.022486
C	-1.881145	2.186144	0.020516
N	-0.571651	2.240737	0.008287
H	-1.902831	-2.021219	0.010701
H	2.196698	-1.240540	-0.027016
H	-2.533769	3.048019	0.028929
H	-3.369275	0.660488	0.031365
Li	0.702891	-3.597914	-0.018125

P739Li$^+$

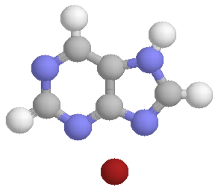

E:	-419.428334		
C	-1.025688	-1.293156	0.007674
N	0.245943	-1.697052	-0.004901
C	1.237333	-0.797981	-0.013891
N	1.090622	0.535070	-0.011605
C	-0.184508	0.928265	0.000961
C	-1.291080	0.075061	0.011157
N	-2.374434	0.940757	0.022289
C	-1.883796	2.211463	0.018424
N	-0.563196	2.251093	0.005609
H	-1.794152	-2.059812	0.014654
H	2.249387	-1.185242	-0.023942
H	-2.532044	3.075517	0.025346
H	-3.355185	0.694854	0.031633
Li	1.460139	2.553085	-0.013368

P91Li$^+$

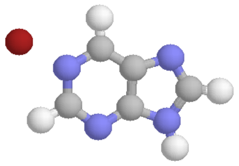

E:	-419.413619		
C	-1.115976	-1.302951	0.003584
N	0.100822	-1.898457	-0.004643
C	1.206898	-1.106845	-0.010860
N	1.241231	0.214921	-0.009832
C	0.037526	0.774394	-0.001679
C	-1.196924	0.075340	0.005414
N	-2.255632	0.956771	0.012751
C	-1.692960	2.134503	0.010940
N	-0.307227	2.092075	0.001518
H	0.328122	2.879209	-0.001960
H	-2.002249	-1.929081	0.008498
H	2.167326	-1.613325	-0.017451
H	-2.223904	3.075762	0.015214
Li	0.288017	-3.808717	-0.007324

P93Li$^+$

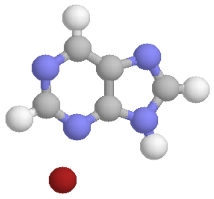

E:	-419.399964		
C	-1.043174	-1.307097	0.009673
N	0.163289	-1.892112	0.002540
C	1.240469	-1.127564	-0.006108
N	1.295255	0.231375	-0.008914
C	0.079763	0.802104	-0.001701
C	-1.135681	0.078169	0.007830
N	-2.212001	0.941582	0.013460
C	-1.685048	2.128414	0.008237
N	-0.290957	2.116616	-0.001667
H	0.299699	2.935348	-0.007303
H	-1.917997	-1.948730	0.016701
H	2.195193	-1.650107	-0.011691
H	-2.231830	3.060572	0.010334
Li	3.117741	0.841231	-0.022332

**P97Li$^+$**<br>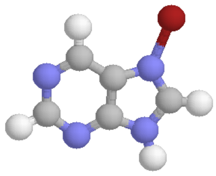	*E*: -419.409825<br>C -1.056637 -1.358372 0.010086<br>N 0.143413 -1.927425 0.002988<br>C 1.235155 -1.141241 -0.005730<br>N 1.265150 0.194842 -0.008215<br>C 0.059322 0.731052 -0.001031<br>C -1.156835 0.033270 0.008345<br>N -2.218745 0.947165 0.013849<br>C -1.638027 2.140177 0.008041<br>N -0.287618 2.071027 -0.001077<br>H 0.357648 2.850986 -0.006825<br>H -1.918842 -2.021983 0.017005<br>H 2.193177 -1.648050 -0.011266<br>H -2.160812 3.085470 0.009992<br>Li -4.080810 0.519593 0.027259
**P123456Li$^+$**	Structure not found for the π-adduct
**P56789Li$^+$**	Structure not found for the π-adduct
**Im13Li$^+$/Im31LI$^+$**<br>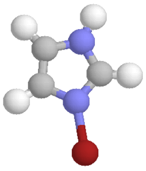	*E*: -233.652192<br>C -1.579834 -0.680861 0.000000<br>N -1.624497 0.648127 0.000000<br>C -0.300711 1.073063 0.000000<br>C 0.527206 -0.009682 0.000000<br>N -0.303287 -1.109427 0.000000<br>H -0.007895 -2.075933 0.000000<br>Li -3.125499 1.809452 0.000000<br>H -0.030292 2.116565 0.000000<br>H 1.600348 -0.094273 0.000000<br>H -2.426788 -1.348781 0.000000
**Im12345Li$^+$**	Structure not found for the π-adduct
**Pym1Li$^+$/Pym3Li$^+$**<br>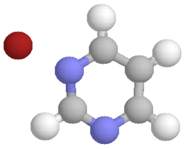	*E*: -271.744446<br>C -1.184696 -0.241048 0.000000<br>N -1.238138 1.108941 0.000000<br>C -0.049398 1.752059 0.000000<br>C 1.150399 1.063201 0.000000<br>C 1.078962 -0.328870 0.000000<br>N -0.089623 -0.979476 0.000000<br>H -2.132170 -0.774028 0.000000<br>H -0.070701 2.837180 0.000000<br>H 2.097527 1.586394 0.000000<br>H 1.974825 -0.941769 0.000000<br>Li -2.950448 2.010645 0.000000
**Pym123456Li$^+$**<br>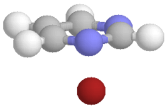	*E*: -271.709420<br>Li -1.246548 -0.057935 -1.274635<br>C 0.126455 -0.472170 -3.280506<br>N 0.842339 0.109060 -2.301086<br>C 0.443042 1.325955 -1.899230<br>N -0.671114 1.957446 -2.301241<br>C -1.382095 1.370200 -3.280636<br>C -1.007753 0.137881 -3.825515<br>H 0.466795 -1.447799 -3.614882<br>H 1.052054 1.824671 -1.150975<br>H -2.271383 1.896294 -3.615121<br>H -1.574462 -0.326203 -4.623842

e) Sodiated neutral isomers

**P17Na+**<br>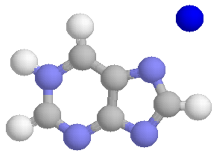	*E*: -574.179444<br>C -1.015288 -1.330436 0.009962<br>N 0.272067 -1.778648 -0.008311<br>C 1.352738 -0.936674 -0.022580<br>N 1.260281 0.361370 -0.020150<br>C 0.008492 0.859037 -0.002303<br>C -1.181892 0.027469 0.013479<br>N -2.274892 0.861166 0.029438<br>C -1.691275 2.086243 0.022395<br>N -0.352388 2.146932 0.003735<br>H -1.796917 -2.078874 0.020175<br>H 2.324162 -1.417116 -0.036509<br>H -2.282507 2.995003 0.031389<br>H 0.446106 -2.776386 -0.011614<br>Na -4.534888 0.737773 0.060625
**P139Na+**<br>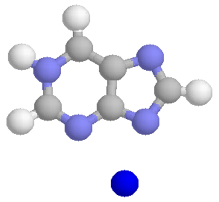	*E*: -574.196872<br>C -1.034473 -1.264340 0.008049<br>N 0.283263 -1.637684 -0.008403<br>C 1.323120 -0.755604 -0.019968<br>N 1.159773 0.545000 -0.016410<br>C -0.128821 0.957499 -0.000271<br>C -1.276645 0.081519 0.012594<br>N -2.404066 0.849910 0.027111<br>C -1.921301 2.082994 0.022886<br>N -0.544255 2.221931 0.006414<br>H -1.780954 -2.047540 0.016138<br>H 2.316571 -1.185473 -0.032540<br>H -2.564209 2.952706 0.031811<br>H 0.506064 -2.626083 -0.012309<br>Na 1.596072 3.069584 -0.018402
**P31Na+**<br>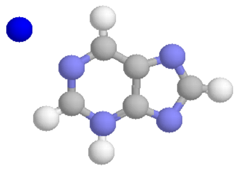	*E*: -574.165685<br>C -1.028503 -1.226873 0.004365<br>N 0.239888 -1.766588 -0.011335<br>C 1.283109 -0.955604 -0.020036<br>N 1.187618 0.391954 -0.014522<br>C -0.039158 0.984761 0.000822<br>C -1.212064 0.127563 0.010893<br>N -2.302484 0.943873 0.025693<br>C -1.759171 2.155885 0.023491<br>N -0.372606 2.246603 0.008466<br>H -1.866420 -1.916089 0.011317<br>H 2.286837 -1.360823 -0.032363<br>H -2.359898 3.055420 0.033162<br>H 2.025769 0.967210 -0.021824<br>Na 0.284441 -4.088853 -0.019660
**P37Na+**<br>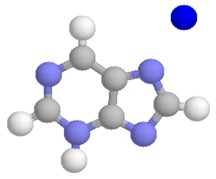	*E*: -574.185701<br>C -1.024791 -1.246397 0.009353<br>N 0.216246 -1.781264 -0.005907<br>C 1.259961 -0.989027 -0.017334<br>N 1.177070 0.367033 -0.014821<br>C -0.044188 0.955498 0.000288<br>C -1.205566 0.122189 0.013076<br>N -2.292856 0.974392 0.027077<br>C -1.713860 2.193362 0.021506<br>N -0.363081 2.244146 0.005393<br>H -1.845963 -1.957239 0.018364<br>H 2.257282 -1.412070 -0.029639<br>H -2.295460 3.106752 0.029435<br>H 2.016198 0.940879 -0.024124<br>Na -4.535852 0.629507 0.053124

**P39Na⁺**<br>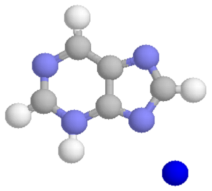	*E*: -574.175157<br>C -1.073967 -1.228367 0.005683<br>N 0.165998 -1.770400 -0.006484<br>C 1.208818 -0.984917 -0.014606<br>N 1.137301 0.379547 -0.011556<br>C -0.081809 0.976676 0.000543<br>C -1.242157 0.142721 0.009567<br>N -2.347380 0.955482 0.021092<br>C -1.832573 2.160476 0.017864<br>N -0.437168 2.265854 0.006141<br>H -1.912847 -1.915599 0.012136<br>H 2.206545 -1.407887 -0.024338<br>H -2.455003 3.047875 0.024952<br>H 1.995975 0.918913 -0.018354<br>Na 0.303616 4.418567 0.003721
**P71Na⁺**<br>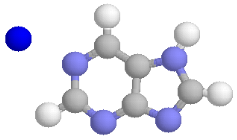	*E*: -574.185429<br>C -1.103120 -1.269928 0.004857<br>N 0.164947 -1.730301 -0.008485<br>C 1.171301 -0.817077 -0.016020<br>N 1.067699 0.497712 -0.011895<br>C -0.183311 0.961100 0.001278<br>C -1.312903 0.093307 0.010129<br>N -2.400179 0.935994 0.022631<br>C -1.891183 2.214268 0.020747<br>N -0.582945 2.269115 0.008267<br>H -1.910555 -1.995009 0.010940<br>H 2.182584 -1.216238 -0.026708<br>H -2.544911 3.075212 0.029111<br>H -3.378784 0.687383 0.031510<br>Na 0.840559 -3.934319 -0.020634
**P739Na⁺**<br>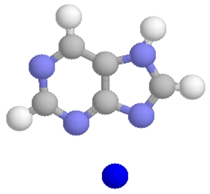	*E*: -574.208969<br>N -1.850076 -1.290023 0.294310<br>C -1.969803 0.026116 0.121726<br>C -0.842921 0.755984 -0.246520<br>C 0.357180 0.051165 -0.413967<br>N 0.481428 -1.267878 -0.241884<br>C -0.658833 -1.874880 0.110941<br>N -0.506763 2.070707 -0.523444<br>C 0.820488 2.090063 -0.829670<br>N 1.382480 0.897094 -0.776611<br>H -2.949526 0.466666 0.278739<br>H -0.618648 -2.947059 0.264643<br>H 1.332887 3.005758 -1.085841<br>H -1.116771 2.876467 -0.505883<br>Na 2.807209 -1.033398 -0.808100
**P91Na⁺**<br>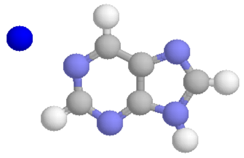	*E*: -574.193963<br>C -1.111536 -1.277724 0.003708<br>N 0.100728 -1.872280 -0.004487<br>C 1.201478 -1.080336 -0.010692<br>N 1.238117 0.244504 -0.009718<br>C 0.034385 0.802761 -0.001595<br>C -1.197490 0.102511 0.005532<br>N -2.258612 0.982257 0.012967<br>C -1.697860 2.161347 0.010867<br>N -0.313291 2.121824 0.001538<br>H 0.319832 2.910259 -0.001953<br>H -1.999123 -1.902333 0.008735<br>H 2.163733 -1.583156 -0.017215<br>H -2.230929 3.101317 0.015098<br>Na 0.325638 -4.167351 -0.008615

**P93Na⁺** 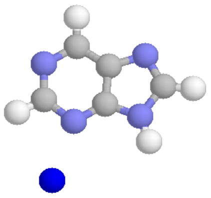	*E*: -574.181485<br>C -1.072773 -1.312047 0.009294<br>N 0.133263 -1.894369 0.003129<br>C 1.210980 -1.124167 -0.004648<br>N 1.266631 0.229393 -0.007500<br>C 0.051892 0.793863 -0.000952<br>C -1.165716 0.073792 0.007624<br>N -2.241021 0.939340 0.012880<br>C -1.710569 2.125551 0.007882<br>N -0.318559 2.111139 -0.000802<br>H 0.275774 2.926497 -0.004325<br>H -1.947419 -1.953967 0.015520<br>H 2.163616 -1.650376 -0.009174<br>H -2.256611 3.058126 0.009883<br>Na 3.485231 0.887026 -0.029751
**P97Na⁺** 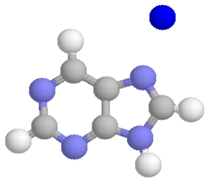	*E*: -574.190315<br>C -1.040189 -1.341511 0.009939<br>N 0.154277 -1.922318 0.002829<br>C 1.251806 -1.143296 -0.006096<br>N 1.292409 0.191746 -0.008499<br>C 0.090333 0.739354 -0.001284<br>C -1.133078 0.050259 0.008189<br>N -2.189128 0.964790 0.013898<br>C -1.604053 2.150402 0.007558<br>N -0.247525 2.078540 -0.001227<br>H 0.400890 2.855188 -0.006917<br>H -1.908505 -1.997857 0.017019<br>H 2.205670 -1.657916 -0.011282<br>H -2.119718 3.099554 0.009262<br>Na -4.417649 0.409576 0.030030
**P123456Na⁺**	Structure not found for the π-adduct
**P56789Na⁺**	Structure not found for the π-adduct
**Im13Na⁺/Im31Na⁺** 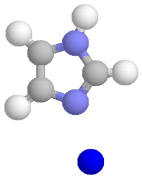	*E*: -388.431056<br>N -0.562534 -0.949287 0.000000<br>C -0.479845 0.399235 0.000000<br>N 0.786857 0.791552 0.000000<br>C 1.541288 -0.372339 0.000000<br>C 0.716248 -1.459766 0.000000<br>H -1.348661 1.038521 0.000000<br>H 2.619377 -0.361507 0.000000<br>H 0.917835 -2.517214 0.000000<br>H -1.417035 -1.488071 0.000000<br>Na 1.675190 2.886936 0.000000
**Im12345Na⁺**	Structure not found for the π-adduct
**Pym1Na⁺/Pym3Na⁺** 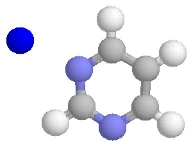	*E*: -426.526113<br>C -1.527978 -0.263846 0.000000<br>N -0.395510 -0.973672 0.000000<br>C 0.737041 -0.288967 0.000000<br>N 0.863004 1.051877 0.000000<br>C -0.288736 1.752988 0.000000<br>C -1.525467 1.129571 0.000000<br>H -2.454962 -0.828400 0.000000<br>H 1.653719 -0.873168 0.000000<br>H -0.212121 2.835652 0.000000<br>H -2.443798 1.701877 0.000000<br>Na 2.979978 2.009687 0.000000
**Pym123456Na⁺**	Structure not found for the π-adduct

f) Lithiated monoanion isomers

$P^{-}39Li^{+}$

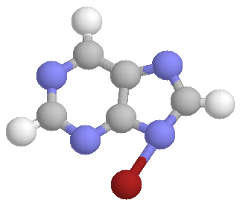

E: -419.037169

C	-0.957193	-1.214334	0.010812
N	0.319715	-1.639675	-0.006645
C	1.306071	-0.739065	-0.020588
N	1.170093	0.595932	-0.019322
C	-0.113956	0.999049	-0.001905
C	-1.242817	0.145354	0.014138
N	-2.371099	0.933331	0.029569
C	-1.878559	2.167246	0.022257
N	-0.512880	2.299496	0.003066
H	-1.730669	-1.976602	0.021863
H	2.319825	-1.125319	-0.034296
H	-2.518463	3.039816	0.030761
Li	1.373361	2.575022	-0.021949

$P^{-}1Li^{+}$

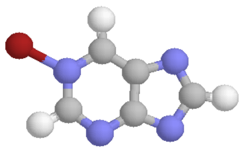

E: -418.993596

C	-0.988681	-0.653895	-0.000006
N	0.233870	-1.258856	-0.000047
C	1.066354	-0.212619	0.000034
C	0.283065	1.010990	0.000003
N	-1.041936	0.684143	0.000057
H	-1.894378	-1.248706	-0.000016
N	2.417069	-0.207029	0.000027
C	0.963959	2.202016	-0.000014
N	2.329360	2.200979	-0.000008
C	2.960749	0.988677	0.000012
H	0.456347	3.163813	-0.000012
H	4.050172	1.028587	0.000012
Li	3.332661	3.739881	-0.000031

$P^{-}7Li^{+}$

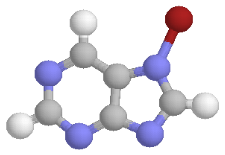

E: -419.008129

C	-0.997998	-0.630327	0.000010
N	0.185575	-1.219125	0.000017
C	1.060362	-0.162078	0.000007
C	0.325933	1.055961	-0.000001
N	-1.024822	0.741646	-0.000014
Li	-2.437647	1.882970	-0.000022
H	-1.918945	-1.203234	0.000024
N	2.397704	-0.200916	-0.000019
C	1.051568	2.239341	0.000014
N	2.386152	2.207823	-0.000012
C	2.977848	0.996828	-0.000099
H	0.584957	3.225532	0.000024
H	4.063683	1.006910	0.000072

$P^{-}123456Li^{+}$

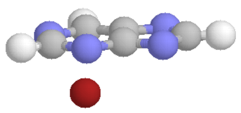

E: -418.992569

Li	-0.013326	-0.754301	-1.545878
C	-1.227536	-1.394955	0.264241
N	-0.023780	-2.015908	0.207751
C	1.092273	-1.246210	0.186681
N	1.157824	0.091394	0.116070
C	-0.037400	0.725414	0.174673
C	-1.299068	-0.004851	0.261316
N	-2.318872	0.893745	0.261455
C	-1.659040	2.062097	0.182563
N	-0.302477	2.039490	0.110681
H	-2.109567	-2.028752	0.289747
H	2.036561	-1.781439	0.178765
H	-2.194581	3.003446	0.170694

P^{-}56789Li^{+}

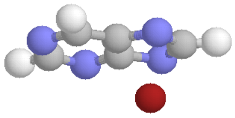

E:	-419.003473		
C	-1.454379	-1.244195	-0.001735
N	-0.269852	-1.839730	0.001883
C	0.838957	-1.054847	0.009831
N	0.909060	0.266950	0.015892
C	-0.295224	0.875169	-0.010436
C	-1.530775	0.157528	-0.009959
N	-2.575522	1.062005	-0.005787
C	-1.927719	2.248906	-0.057878
N	-0.569271	2.220082	0.002322
H	-2.341320	-1.873105	-0.014541
H	1.783540	-1.589977	0.013434
H	-2.469399	3.185997	-0.103193
Li	-1.441276	1.412067	1.769376

Im^{-}1Li^{+}/Im^{-}3Li^{+}

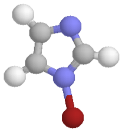

E:	-233.232136		
C	-0.834389	-0.572697	0.000000
N	0.370205	-1.121190	0.000000
C	1.225750	-0.046856	0.000000
C	0.501107	1.123257	0.000000
N	-0.845699	0.795707	0.000000
Li	-2.275890	1.891858	0.000000
H	-1.748005	-1.155925	0.000000
H	2.298209	-0.177493	0.000000
H	0.843203	2.149200	0.000000

Im^{-}12345Li^{+}

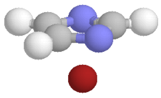

E:	-233.243556		
N	-1.181280	-0.381751	0.022069
C	0.126023	-0.739938	0.039989
N	1.003819	0.292949	0.023254
C	0.195842	1.404041	0.054412
C	-1.140417	0.991441	0.053686
H	0.445213	-1.773700	0.053948
H	0.607622	2.402962	0.086839
H	-2.043729	1.584296	0.085401
Li	-0.186884	0.276519	-1.697657

g) Sodiated monoanion isomers

P^{-}39Na^{+}

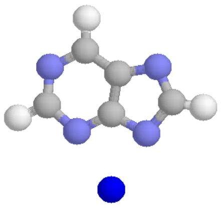

E:	-573.805625		
C	2.086653	0.438263	-0.000723
N	2.286625	-0.891678	-0.000442
C	1.220991	-1.698525	0.000391
N	-0.069717	-1.335190	0.000865
C	-0.255007	-0.000055	0.000569
C	0.798068	0.954596	-0.000103
N	0.243467	2.213347	0.000665
C	-1.061246	1.959467	-0.000185
N	-1.451218	0.645653	-0.000221
H	2.969267	1.071609	-0.000883
H	1.423099	-2.765240	0.000396
H	-1.797273	2.753595	-0.000164
Na	-2.399632	-1.400670	-0.000464

**P⁻1Na⁺**<br>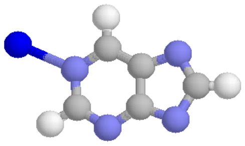	*E:* -573.764320<br>C -1.003314 -0.681483 0.000132<br>N 0.216818 -1.285046 0.000289<br>C 1.050626 -0.235100 -0.000151<br>C 0.266687 0.985599 -0.000042<br>N -1.057878 0.658693 -0.000347<br>H -1.909775 -1.275544 0.000082<br>N 2.399587 -0.226787 -0.000116<br>C 0.950941 2.178533 0.000048<br>N 2.311398 2.180722 0.000045<br>C 2.941780 0.973260 -0.000043<br>H 0.440370 3.139090 0.000023<br>H 4.031607 1.011371 -0.000035<br>Na 3.529784 4.014661 0.000125
**P⁻7Na⁺**<br>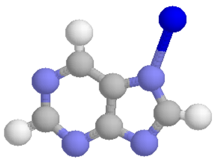	*E:* -573.777053<br>Na -2.620309 2.225883 -0.000016<br>C -1.007264 -0.637693 -0.000004<br>N 0.183092 -1.227466 -0.000026<br>C 1.050429 -0.169224 -0.000007<br>C 0.305739 1.046621 0.000020<br>N -1.038582 0.726934 0.000026<br>H -1.925299 -1.215146 0.000010<br>N 2.389607 -0.195051 -0.000002<br>C 1.025373 2.233432 0.000017<br>N 2.360142 2.214749 -0.000011<br>C 2.960346 1.006739 -0.000008<br>H 0.550678 3.217810 -0.000018<br>H 4.046218 1.025482 0.000020
**P⁻123456Na⁺**	Structure not found for the π-adduct
**P⁻56789Na⁺**	Structure not found for the π-adduct
**Im⁻1Na⁺/Im⁻3Na⁺**<br>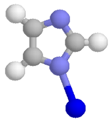	*E:* -387.998233<br>Na -2.577806 2.087626 0.000000<br>C -0.838108 -0.566931 0.000000<br>N 0.368903 -1.121257 0.000000<br>C 1.224075 -0.049130 0.000000<br>C 0.496403 1.122892 0.000000<br>N -0.846509 0.796096 0.000000<br>H -1.751984 -1.150860 0.000000<br>H 2.296854 -0.180647 0.000000<br>H 0.839283 2.149090 0.000000
**Im⁻12345Na⁺**<br>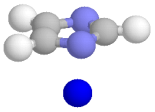	*E:* -388.003849<br>N -1.183319 -0.379697 0.073507<br>C 0.123486 -0.731808 0.089567<br>N 1.004294 0.295778 0.074692<br>C 0.194160 1.406115 0.099000<br>C -1.140242 0.994088 0.098270<br>H 0.443003 -1.766657 0.118949<br>H 0.604624 2.405815 0.149530<br>H -2.042924 1.588324 0.148085<br>Na -0.176874 0.244861 -2.129660

Table S2 Enthalpies and Gibbs energies (*H* and *G*, respectively, in Hartree) calculated for imidazole and pyrimidine derivatives at the DFT(B3LYP)/6-311+G(d,p) and G*n* levels

Derivative	Quantity	DFT	G2	G2MP2	G3	G3B3
**Im$^-$**	*H*	−225.652381	−225.272378	−225.267381	−225.497877	−225.503232
	G	−225.682984	−225.302951	−225.297953	−225.528450	−225.533958
**Im1/Im3**	*H*	−226.207174	−225.826525	−225.821819	−226.053128	−226.058437
	G	−226.238155	−225.857559	−225.852854	−226.084162	−226.089591
**ImH$^+$**	*H*	−226.565141	−226.183714	−226.179157	−226.411010	−226.416159
	G	−226.596196	−226.214831	−226.210274	−226.442128	−226.447385
**Im1Li$^+$/Im3Li$^+$**	*H*	−233.572368	−233.138594	−233.133708	−233.398266	−233.403617
	G	−233.606632	−233.173143	−233.168257	−233.432815	−233.438244
**Im1Na$^+$/Im3Na$^+$**	*H*	−388.351755	−387.543818	−387.539002	−388.027025	−388.032305
	G	−388.388280	−387.580709	−387.575893	−388.063916	−388.069213
**Im$^-$1Li$^+$/Im$^-$3Li$^+$**	*H*	−233.166158	−232.732024	−232.726942	−232.991568	−232.996981
	G	−233.200820	−232.766958	−232.761876	−233.026502	−233.032374
**Im$^-$1Na$^+$/Im$^-$3Na$^+$**	*H*	−387.932871	−387.123652	−387.118652	−387.607632	−387.613954
	G	−387.969878	−387.161901	−387.156900	−387.645880	−387.649122
**Im$^-$12345Li$^+$**	*H*	−233.177204	−232.744654	−232.739461	−233.005681	−233.011171
	G	−233.209548	−–232.777214	−–232.772021	−233.038240	−233.043726
**Im$^-$12345Na$^+$**	*H*	−387.938480	−387.132367	−387.127233	−387.619535	−387.625065
	G	−387.973384	−387.167324	−387.162190	−387.654492	−387.660004
**Pym**	*H*	−264.312056	−263.859968	−263.854343	−264.131615	−264.137333
	G	−264.344561	−263.892481	−263.886855	−264.164128	−264.170004
**Pym1H$^+$/Pym3H$^+$**	*H*	−264.648587	−264.195393	−264.189897	−264.467233	−264.472879
	G	−264.681366	−264.228225	−264.222729	−264.500065	−264.505838
**Pym1Li$^+$/Pym3Li$^+$**	*H*	−271.658882	−271.153683	−271.147968	−271.457983	−271.463789
	G	−271.694868	−271.189899	−271.184185	−271.494199	−271.500095
**Pym123456Li$^+$**	*H*	−217.624449	a	a	a	a
	G	−217.660720	a	a	a	a
**Pym1Na$^+$/Pym3Na$^+$**	*H*	−426.440965	−425.561677	−425.556034	−426.089492	−426.095233
	G	−426.479201	−425.600255	−425.594613	−426.128070	−426.133829

[a] Not calculated.

Table S3 Structures, electronic energies (*E* in Hartree), and atom coordinates (in Å) for deprotonated, neutral [47], protonated, and lithiated forms of purine calculated in aqueous solution at the PCM(water)//B3LYP/6-311+G(d,p) level

a) Deprotonated forms

Structure	Electronic energy and atom coordinates
**P⁻**<br>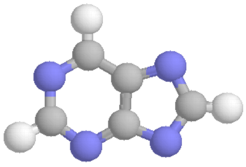	*E*: -411.607184<br>C -0.882662 -1.284561 0.000000<br>N 0.315664 -1.896106 0.000000<br>C 1.418015 -1.120644 0.000000<br>N 1.480966 0.211905 0.000000<br>C 0.280511 0.820815 0.000000<br>C -0.955355 0.100701 0.000000<br>N -1.992209 1.006164 0.000000<br>C -1.341917 2.180858 0.000000<br>N 0.015859 2.154442 0.000000<br>H -1.769154 -1.919478 0.000000<br>H 2.369526 -1.650756 0.000000<br>H -1.881813 3.124403 0.000000

b) Neutral isomers

**P1**<br>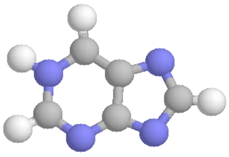	*E*: -412.072756<br>C -0.722803 -1.469618 -0.048051<br>N 0.525116 -2.006185 -0.027300<br>C 1.658925 -1.253614 0.016748<br>N 1.666570 0.056746 0.043257<br>C 0.447745 0.641744 0.023989<br>C -0.791084 -0.101037 -0.022225<br>N -1.833014 0.789057 -0.031087<br>N 0.166979 1.952591 0.043582<br>C -1.198896 1.964992 0.009845<br>H -1.564479 -2.156992 -0.083398<br>H 2.592878 -1.810724 0.029266<br>H -1.745961 2.904098 0.014716<br>H 0.627625 -3.034276 -0.044802
**P2**<br>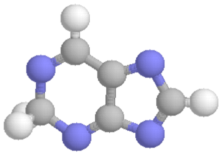	*E*: -412.002504<br>C -0.709694 -1.561542 0.024264<br>N 0.431383 -2.115717 -0.190710<br>C 1.619217 -1.311072 -0.335084<br>N 1.601712 0.133588 -0.249683<br>C 0.453485 0.645539 -0.036124<br>C -0.796217 -0.125141 0.120606<br>N -1.791460 0.682967 0.327055<br>N 0.119185 2.000028 0.095392<br>C -1.164941 1.963173 0.299645<br>H -1.599840 -2.185838 0.131295<br>H 2.074733 -1.572952 -1.303528<br>H -1.770938 2.854179 0.448332<br>H 2.349130 -1.670824 0.407745

**P3**<br>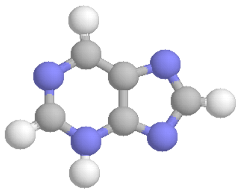	*E*: -412.069375<br>C -0.699570 -1.521945 -0.029917<br>N 0.516303 -2.125558 -0.006327<br>C 1.600644 -1.378503 0.025612<br>N 1.588526 -0.025623 0.035838<br>C 0.401792 0.627082 0.015144<br>C -0.802986 -0.147024 -0.019783<br>N -1.855929 0.738699 -0.036009<br>N 0.131330 1.929094 0.020402<br>C -1.242973 1.921616 -0.011550<br>H -1.571239 -2.174501 -0.055764<br>H 2.582242 -1.846589 0.043772<br>H -1.797193 2.856175 -0.016152<br>H 2.484199 0.496634 0.059683
**P4**<br>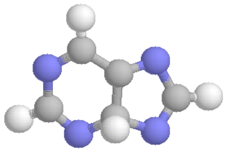	*E*: -411.988544<br>C -0.732781 -1.567053 0.161968<br>N 0.299858 -2.132264 -0.364509<br>C 1.387654 -1.304578 -0.759952<br>N 1.590704 -0.063860 -0.520833<br>C 0.597678 0.551006 0.329180<br>C -0.731869 -0.125801 0.339634<br>N -1.706224 0.720036 0.337261<br>N 0.257584 1.965011 0.176261<br>C -1.024269 1.979022 0.253980<br>H -1.607021 -2.170072 0.399565<br>H 2.129061 -1.835786 -1.349622<br>H -1.610322 2.889998 0.249663<br>H 1.016344 0.497822 1.354271
**P5**<br>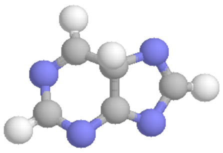	*E*: -412.000831<br>C -0.667742 -1.503061 -0.177928<br>N 0.417074 -2.094747 0.177291<br>C 1.436124 -1.312208 0.761588<br>N 1.527826 -0.020197 0.782422<br>C 0.441740 0.623255 0.237239<br>C -0.881035 -0.049543 0.125953<br>N -1.661443 0.846688 -0.716167<br>N 0.433759 1.781044 -0.348523<br>C -0.871497 1.854098 -0.892674<br>H -1.472237 -2.085378 -0.635141<br>H 2.250345 -1.893170 1.194158<br>H -1.166100 2.743770 -1.445623<br>H -1.387073 -0.068118 1.121977
**P6**<br>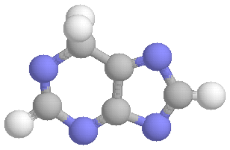	*E*: -411.999290<br>C -0.676398 -1.599736 0.006188<br>N 0.681331 -2.092117 -0.156242<br>C 1.659963 -1.272230 -0.225962<br>N 1.635068 0.148397 -0.165557<br>C 0.469496 0.659377 -0.022189<br>C -0.787076 -0.130527 0.077436<br>N -1.790498 0.671238 0.214957<br>N 0.123360 2.006974 0.067883<br>C -1.170072 1.960956 0.203130<br>H -1.103963 -2.067218 0.907086<br>H 2.664522 -1.676162 -0.347693<br>H -1.790815 2.848817 0.303101<br>H -1.284989 -1.997016 -0.821316

P7

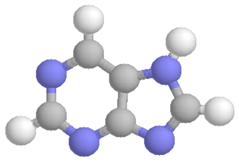

E: -412.079446

C	-0.618484	-1.567087	-0.007143
N	0.595690	-2.130865	0.001880
C	1.674862	-1.324277	0.011644
N	1.693887	0.008956	0.013602
C	0.482135	0.575811	0.004714
C	-0.710885	-0.184331	-0.005953
N	-1.725721	0.744805	-0.012546
N	0.190487	1.920578	0.004092
C	-1.130087	1.968061	-0.006620
H	-1.489369	-2.221497	-0.014849
H	2.641957	-1.824115	0.018579
H	-1.718445	2.880327	-0.010166
H	-2.738254	0.564664	-0.020758

P8

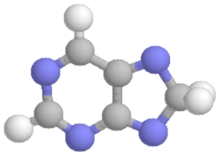

E: -412.008798

C	-0.563266	-1.636935	-0.010340
N	0.635233	-2.120550	-0.006752
C	1.722931	-1.221971	0.002708
N	1.720138	0.071070	0.008699
C	0.457436	0.631968	0.005161
C	-0.754102	-0.201634	-0.004607
N	-1.826329	0.518499	-0.006494
N	0.146323	1.887148	0.009455
C	-1.302042	1.880738	0.002420
H	-1.411311	-2.318984	-0.017641
H	2.686582	-1.722301	0.005084
H	-1.688542	2.413457	0.881492
H	-1.680030	2.421660	-0.875343

P9

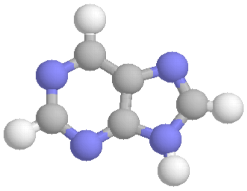

E: -412.079810

C	-0.683751	-1.558978	0.000000
N	0.516123	-2.156987	0.000000
C	1.616426	-1.383512	0.000000
N	1.671403	-0.047378	0.000000
C	0.469133	0.521427	0.000000
C	-0.761800	-0.171975	0.000000
N	-1.816968	0.726889	0.000000
N	0.133836	1.848264	0.000000
C	-1.237464	1.905722	0.000000
H	0.779028	2.650255	0.000000
H	-1.569401	-2.194038	0.000000
H	2.571453	-1.905817	0.000000
H	-1.757510	2.858465	0.000000

c) Protonated isomers

P13H⁺/P31H⁺

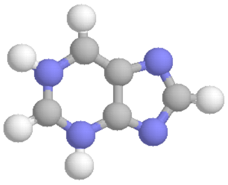

E: -412.510929

C	-1.002181	-1.306122	0.008858
N	0.289731	-1.766349	-0.008040
C	1.366557	-0.981795	-0.020154
N	1.234994	0.338842	-0.016198
C	-0.005322	0.904355	0.000052
C	-1.171095	0.047508	0.013165
N	-2.271167	0.863255	0.027923
C	-1.735216	2.077939	0.023439
N	-0.354000	2.170208	0.006402
H	-1.790826	-2.055802	0.017395
H	2.358775	-1.429366	-0.033264
H	-2.342760	2.978938	0.032204
H	0.451700	-2.795104	-0.012278
H	2.093280	0.934301	-0.025364

**P17H+/P71H+**<br>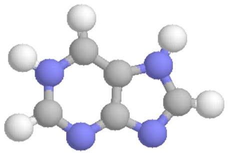	*E*: -412.529404<br>C -1.013679 -1.347531 0.011004<br>N 0.268401 -1.772195 -0.007293<br>C 1.336590 -0.930273 -0.020964<br>N 1.232201 0.377022 -0.017778<br>C -0.021619 0.855374 0.000206<br>C -1.174082 0.013764 0.014800<br>N -2.240074 0.872503 0.030587<br>C -1.722357 2.126271 0.025626<br>N -0.396435 2.162160 0.007327<br>H -1.804142 -2.095744 0.021031<br>H 2.314818 -1.407216 -0.035199<br>H -2.366529 3.001301 0.035860<br>H 0.454774 -2.794770 -0.010842<br>H -3.243716 0.626713 0.043915
**P19H+/P91H+**<br>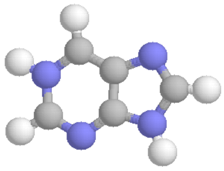	E: -412.530274<br>C -0.994060 -1.296854 0.009890<br>N 0.279510 -1.749627 -0.007269<br>C 1.366504 -0.934974 -0.021976<br>N 1.289907 0.377022 -0.020808<br>C 0.043022 0.851800 -0.003807<br>C -1.147202 0.069524 0.011930<br>N -2.256490 0.887851 0.026944<br>C -1.759129 2.100693 0.020662<br>N -0.381698 2.136539 0.002159<br>H -1.797335 -2.031268 0.020872<br>H 2.335436 -1.430285 -0.035204<br>H -2.340723 3.018139 0.028700<br>H 0.444909 -2.775808 -0.009097<br>H 0.209169 2.984837 -0.005539
**P37H+/P73H+**<br>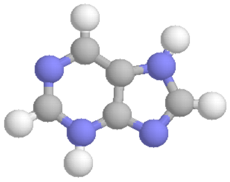	*E*: -412.525251<br>C -0.953221 -1.335330 0.010656<br>N 0.299497 -1.830730 -0.006447<br>C 1.326071 -1.000699 -0.019696<br>N 1.207033 0.343585 -0.016929<br>C -0.022638 0.896119 -0.000396<br>C -1.142260 0.033177 0.014056<br>N -2.215532 0.892281 0.029302<br>C -1.712609 2.147721 0.022590<br>N -0.380667 2.194880 0.005117<br>H -1.778242 -2.047036 0.021414<br>H 2.341674 -1.391882 -0.033326<br>H -2.357972 3.021579 0.031777<br>H 2.058840 0.943731 -0.027669<br>H -3.216674 0.636935 0.043032
**P39H+/P93H+**<br>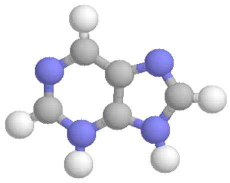	*E*: -412.520225<br>C -0.946037 -1.279650 0.012266<br>N 0.293087 -1.813138 -0.004743<br>C 1.341549 -1.016432 -0.020213<br>N 1.261566 0.336003 -0.020110<br>C 0.044259 0.897605 -0.003552<br>C -1.118475 0.094159 0.013421<br>N -2.233238 0.911921 0.028164<br>C -1.752348 2.127334 0.020172<br>N -0.367647 2.176650 0.001469<br>H -1.789203 -1.969927 0.024528<br>H 2.347714 -1.431558 -0.033776<br>H -2.336733 3.042820 0.027479<br>H 2.131977 0.910890 -0.032569<br>H 0.218862 3.029412 -0.007126

P79H⁺/P97H⁺

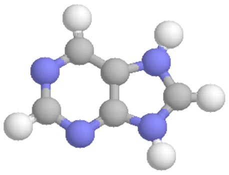

E: -412.526197

C	-0.947364	-1.334143	0.015855
N	0.290540	-1.828312	-0.002100
C	1.333593	-0.975427	-0.019622
N	1.285771	0.358548	-0.021150
C	0.049900	0.833630	-0.003137
C	-1.108062	0.047324	0.015668
N	-2.178728	0.926531	0.030482
C	-1.707126	2.169592	0.021081
N	-0.371126	2.151325	0.001117
H	-1.788439	-2.026563	0.030074
H	2.323483	-1.427098	-0.034024
H	-2.317510	3.068689	0.028755
H	-3.185835	0.679786	0.046153
H	0.235083	2.992896	-0.009493

d) Lithiated neutral isomers

P13Li⁺

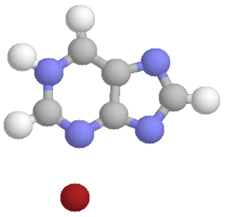

E: -419.569413

C	-0.927768	-0.460587	-0.023977
N	0.252733	-1.134500	-0.044006
C	1.461775	-0.521986	-0.077118
C	0.473249	1.508578	-0.071479
C	-0.840347	0.905308	-0.037399
N	-1.775347	1.906542	-0.024564
C	-1.016356	3.003792	-0.048904
N	0.342178	2.838094	-0.078760
H	-1.453016	3.998930	-0.047971
N	1.619311	0.783625	-0.091227
H	2.327945	-1.179460	-0.090513
H	-1.841090	-1.050138	0.001556
H	0.236963	-2.168500	-0.034677
Li	3.588320	1.675652	-0.160621

P17Li⁺

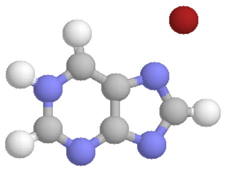

E: -419.573013

C	-1.030310	-1.306660	0.016841
N	0.247055	-1.763869	-0.007028
C	1.332440	-0.943278	-0.027187
N	1.257283	0.364976	-0.025018
C	0.005882	0.871688	-0.001540
C	-1.183230	0.055835	0.020078
N	-2.274431	0.888927	0.037595
C	-1.706398	2.105572	0.027659
N	-0.350434	2.166792	0.004585
H	-1.827337	-2.046471	0.031470
H	2.299409	-1.441153	-0.046072
H	-2.309776	3.009506	0.036979
H	0.411852	-2.785030	-0.010250
Li	-4.338202	0.580026	0.031622

P19Li⁺

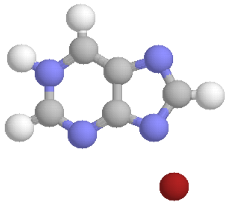

E: -419.573778

C	-1.057269	-1.280127	0.011413
N	0.213926	-1.754590	-0.007755
C	1.310213	-0.948472	-0.021780
N	1.253374	0.361348	-0.016440
C	0.008907	0.882559	-0.002183
C	-1.190994	0.084692	0.014118
N	-2.277667	0.920737	0.026075
C	-1.710734	2.125580	0.015951
N	-0.341722	2.180829	-0.000766
H	-1.866435	-2.006538	0.022644
H	2.270319	-1.459467	-0.036284
H	-2.301558	3.037616	0.019908
H	0.365900	-2.777705	-0.011697
Li	0.853881	3.877960	0.003498

**P139Li+**	Structure not found for the bidentate adduct
**P31Li⁺**<br>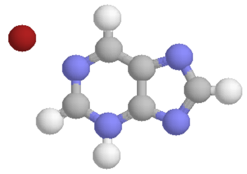	*E*: -419.566369<br>C -0.877116 -0.384019 -0.045951<br>N 0.263540 -1.129041 -0.048367<br>C 1.432433 -0.512549 -0.049451<br>C 0.474540 1.617537 -0.046425<br>C -0.814981 0.990357 -0.045325<br>N -1.755100 1.992676 -0.041697<br>C -1.006866 3.094627 -0.043399<br>N 0.360631 2.939313 -0.044567<br>H -1.445934 4.088465 -0.040662<br>N 1.576129 0.828029 -0.047927<br>H 2.351661 -1.094065 -0.052425<br>H -1.819352 -0.930196 -0.045309<br>Li 0.164286 -3.296964 -0.028673<br>H 2.527498 1.240880 -0.047872
**P37Li⁺**<br>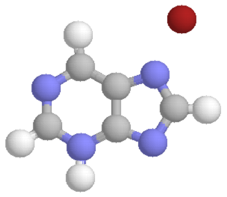	*E*: -419.569632<br>C -1.041933 -1.241117 0.014579<br>N 0.200160 -1.782964 -0.004239<br>C 1.248123 -0.984437 -0.019373<br>N 1.168777 0.365043 -0.016559<br>C -0.048280 0.957121 0.001950<br>C -1.212313 0.128195 0.017774<br>N -2.302933 0.971203 0.031246<br>C -1.740424 2.185721 0.025762<br>N -0.376376 2.248246 0.007193<br>H -1.880470 -1.936557 0.026011<br>H 2.251747 -1.403675 -0.035193<br>H -2.338462 3.092790 0.033924<br>H 2.036229 0.933674 -0.029395<br>Li -4.358705 0.614517 0.032110
**P39Li⁺**<br>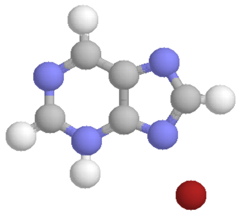	*E*: -419.568811<br>C -0.842273 -0.456697 -0.011590<br>N 0.298021 -1.189952 -0.012247<br>C 1.457464 -0.567255 -0.042990<br>C 0.487633 1.557519 -0.071310<br>C -0.792570 0.921433 -0.043590<br>N -1.747665 1.911486 -0.050940<br>C -1.022550 3.021767 -0.082388<br>N 0.350586 2.884813 -0.096716<br>H -1.472846 4.010548 -0.096475<br>N 1.594179 0.779019 -0.072319<br>H 2.383267 -1.138481 -0.045868<br>H -1.782344 -1.006391 0.015337<br>H 2.543780 1.195429 -0.095474<br>Li 1.640998 4.538552 -0.137640
**P71Li⁺**<br>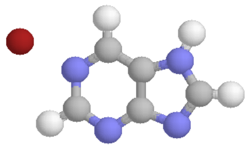	*E*: -419.577825<br>C -1.067445 -1.273768 -0.000217<br>N 0.198269 -1.722059 -0.013834<br>C 1.200339 -0.814719 -0.020373<br>N 1.090133 0.510019 -0.014004<br>C -0.169383 0.961110 0.000241<br>C -1.285961 0.091590 0.006323<br>N -2.383300 0.919911 0.021484<br>C -1.904882 2.193125 0.024351<br>N -0.585133 2.270076 0.011509<br>H -1.872302 -2.007529 0.005311<br>H 2.209328 -1.223466 -0.030126<br>H -2.576281 3.046257 0.036432<br>H -3.374965 0.645078 0.029433<br>Li 0.640785 -3.824405 -0.000798

P739Li$^+$

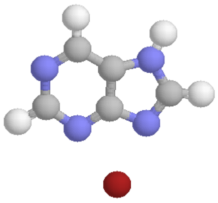

E: -419.571985

C	-1.051962	-1.300014	0.007586
N	0.216993	-1.730432	-0.002990
C	1.212323	-0.824974	-0.012505
N	1.086303	0.503927	-0.013585
C	-0.178136	0.928265	-0.000560
C	-1.291314	0.066262	0.010876
N	-2.381324	0.909219	0.023058
C	-1.897429	2.179653	0.018195
N	-0.575401	2.240920	0.003987
H	-1.846072	-2.045542	0.011562
H	2.225844	-1.221271	-0.022667
H	-2.561593	3.038345	0.024123
H	-3.376380	0.644672	0.036733
Li	1.697489	2.842892	-0.013775

P73Li$^+$

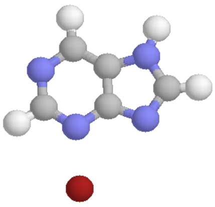

E: -419.577122

C	-0.404591	-0.335865	0.196910
N	0.807465	-0.871378	0.005482
C	1.839812	-0.050531	-0.248539
C	0.598926	1.825595	-0.146418
C	-0.544213	1.040324	0.125575
N	-1.574361	1.941474	0.256404
C	-1.035342	3.174794	0.067807
N	0.263737	3.156125	-0.176263
H	-1.644288	4.071872	0.119401
N	1.811342	1.282780	-0.337968
H	2.807649	-0.525303	-0.397195
H	-1.237648	-1.007073	0.402521
H	-2.561765	1.736858	0.457374
Li	3.490567	2.566028	-0.765132

P79Li$^+$

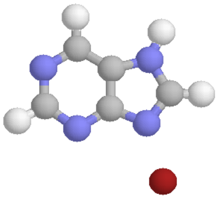

E: -419.578641

C	-0.608652	-0.487533	0.255287
N	0.603412	-1.020214	0.063019
C	1.633249	-0.191732	-0.197033
C	0.392206	1.670809	-0.098542
C	-0.750178	0.889490	0.178563
N	-1.781837	1.792396	0.309012
C	-1.252041	3.023420	0.115120
N	0.049676	3.006517	-0.132977
H	-1.861618	3.920305	0.165060
N	1.601806	1.138240	-0.290624
H	2.601122	-0.666850	-0.347149
H	-1.439773	-1.159561	0.465920
H	-2.769578	1.587168	0.514354
Li	1.322335	4.634394	-0.478310

P91Li$^+$

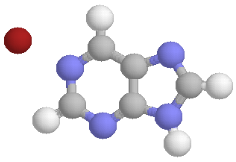

E: -419.578342

C	-1.103424	-1.296418	-0.002739
N	0.106735	-1.882827	-0.010355
C	1.202413	-1.094979	-0.015203
N	1.238076	0.237564	-0.012318
C	0.029898	0.793845	-0.003255
C	-1.193788	0.086763	0.001199
N	-2.258652	0.972159	0.011997
C	-1.692195	2.156957	0.012995
N	-0.319534	2.114611	0.003865
H	0.316142	2.924356	0.003583
H	-1.981384	-1.941568	0.001902
H	2.162162	-1.608433	-0.021471
H	-2.222915	3.103874	0.020808
Li	0.291535	-4.022305	0.013162

**P93Li$^+$**<br>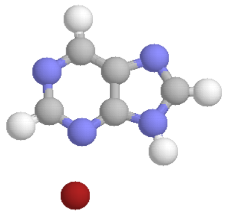	*E*: -419.575903<br>C -1.082305 -1.329335 0.007697<br>N 0.119038 -1.925717 0.003065<br>C 1.215296 -1.155889 -0.002214<br>N 1.269853 0.185511 -0.003199<br>C 0.064655 0.758263 0.001670<br>C -1.162308 0.056018 0.007321<br>N -2.222521 0.947595 0.010564<br>C -1.651895 2.129209 0.007221<br>N -0.278983 2.079584 0.001904<br>H 0.357151 2.889388 -0.001145<br>H -1.966798 -1.965908 0.011256<br>H 2.172753 -1.674095 -0.005918<br>H -2.176419 3.079569 0.008300<br>Li 3.217204 1.135608 -0.037463
**P97Li$^+$**<br>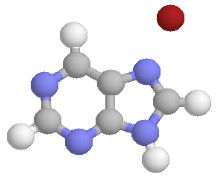	*E*: -419.578340<br>C -1.038138 -1.357814 0.013728<br>N 0.169981 -1.935661 0.003453<br>C 1.258652 -1.146026 -0.008288<br>N 1.291278 0.190227 -0.011094<br>C 0.080983 0.738940 -0.000661<br>C -1.137339 0.028315 0.012215<br>N -2.202001 0.920468 0.018941<br>C -1.634369 2.110212 0.010662<br>N -0.270810 2.063342 -0.001305<br>H 0.365032 2.873687 -0.007856<br>H -1.913219 -2.007518 0.022992<br>H 2.221632 -1.653375 -0.016417<br>H -2.168806 3.055076 0.012492<br>Li -4.287335 0.596637 0.014559

e) Lithiated monoanion isomers

**P$^-$1Li$^+$**<br>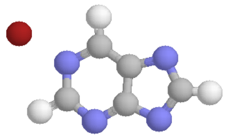	*E*: -419.108755<br>C -1.014418 -0.679738 -0.000298<br>N 0.204777 -1.280732 -0.000069<br>C 1.050863 -0.219872 -0.001294<br>C 0.283360 0.988317 -0.000042<br>N -1.052886 0.661564 -0.001718<br>H -1.927115 -1.270246 -0.000045<br>N 2.397353 -0.229412 -0.000792<br>C 0.979135 2.183981 0.001684<br>N 2.329006 2.176624 0.003139<br>C 2.955265 0.977857 0.001210<br>H 0.484138 3.155010 0.002768<br>H 4.043763 1.014183 0.002210<br>Li 3.435371 3.960444 -0.006743
**P$^-$3Li$^+$**<br>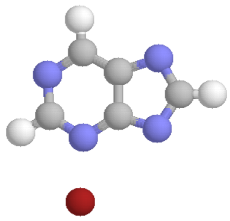	*E*: -419.108409<br>C -0.695932 -0.410005 -0.013972<br>N 0.389991 -1.206510 -0.022303<br>C 1.598687 -0.623763 -0.040792<br>C 0.787264 1.485250 -0.048577<br>C -0.546397 0.966943 -0.028187<br>N -1.426176 2.025116 -0.026126<br>C -0.600230 3.081268 -0.045425<br>N 0.737932 2.839411 -0.059401<br>H -0.982638 4.098768 -0.050966<br>N 1.875072 0.686554 -0.052458<br>H 2.455478 -1.296069 -0.045089<br>H -1.671859 -0.895523 0.004843<br>Li 3.849979 1.392970 0.009473

**P⁻7Li⁺**<br>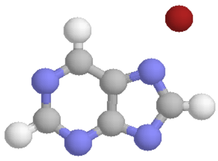	*E:* -419.110910<br>C -0.989727 -0.604177 -0.004844<br>N 0.211956 -1.218913 0.000698<br>C 1.081822 -0.170579 0.000395<br>C 0.343654 1.050979 -0.005970<br>N -1.001135 0.744174 -0.009341<br>Li -2.695233 1.904134 0.027225<br>H -1.915678 -1.173424 -0.005610<br>N 2.425779 -0.212694 0.005898<br>C 1.070824 2.232329 -0.007284<br>N 2.414223 2.194169 -0.002085<br>C 3.010665 0.986147 0.004425<br>H 0.597458 3.214579 -0.012759<br>H 4.099763 0.994606 0.009254
**P⁻9Li⁺**<br>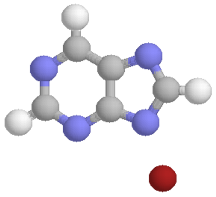	*E:* -419.111345<br>C -1.019732 -1.280594 0.023634<br>N 0.213019 -1.817046 0.006344<br>C 1.265429 -0.976605 -0.020650<br>N 1.245340 0.357853 -0.031147<br>C 0.010931 0.888036 -0.014542<br>C -1.176521 0.097719 0.012225<br>N -2.269444 0.936761 0.022591<br>C -1.703326 2.147245 0.002896<br>N -0.342032 2.204871 -0.019720<br>H -1.866397 -1.967089 0.047019<br>H 2.248006 -1.446138 -0.034985<br>H -2.298599 3.056570 0.004938<br>Li 0.856756 3.858669 0.029157
**P⁻39Li⁺**	Structure not found for the bidentate adduct
**P⁻123456Li⁺**	Structure not found for the π-adduct
**P⁻56789Li⁺**	Structure not found for the π-adduct

Polarizable Continuum Model (PCM)

Model:	PCM
Atomic radii:	UA0 (Simple United Atom Topological Model)
Polarization charges:	Total charges
Charge compensation:	None
Solution method:	Matrix inversion
Cavity:	GePol (RMin=0.200 OFac=0.890)<br>Default sphere list used, NSphG= 9<br>Tesserae with average area of 0.200 Ang**2
1st derivatives:	Analytical V*U(x)*V algorithm (CHGder, D1EAlg=0)<br>Cavity 1st derivative terms included
Solvent:	Water<br>Eps = 78.390000<br>Eps(inf)= 1.776000<br>RSolv = 1.385000 Ang

Table S4 HOMED indices for the imidazole (HOMED5) and pyrimidine (HOMED6) fragments, and also for the entire purine system (HOMED10) of the hydrated deprotonated **P**$^-$, neutral **P** [47], protonated **PH**$^+$, lithiated neutral **PLi**$^+$, and lithiated monoanionic **P**$^-$**Li**$^+$ forms estimated at the PCM(water)//B3LYP/6-311+G(d,p) level

Structure	HOMED5	HOMED6	HOMED10	Structure	HOMED5	HOMED6	HOMED10
**P**$^-$	0.936	0.976	0.960	**P13Li**$^+$	0.924	0.925	0.934
**P1**	0.927	0.923	0.934	**P17Li**$^+$	0.927	0.930	0.936
**P2**	0.656	0.424	0.519	**P19Li**$^+$	0.927	0.933	0.936
**P3**	0.927	0.953	0.943	**P31Li**$^+$	0.925	0.950	0.941
**P4**	0.411	0.474	0.423	**P37Li**$^+$	0.938	0.960	0.951
**P5**	0.473	0.543	0.505	**P39Li**$^+$	0.923	0.958	0.942
**P6**	0.616	0.369	0.480	**P71Li**$^+$	0.933	0.986	0.958
**P7**	0.928	0.991	0.963	**P739Li**$^+$	0.936	0.995	0.962
**P8**	0.418	0.731	0.554	**P73Li**$^+$	0.936	0.991	0.962
**P9**	0.907	0.993	0.947	**P79Li**$^+$	0.930	0.993	0.958
**P13H**$^+$	0.893	0.914	0.911	**P91Li**$^+$	0.910	0.989	0.946
**P17H**$^+$	0.949	0.954	0.952	**P93Li**$^+$	0.923	0.993	0.955
**P19H**$^+$	0.914	0.963	0.936	**P97Li**$^+$	0.913	0.994	0.949
**P37H**$^+$	0.963	0.981	0.971	**P**$^-$**1Li**$^+$	0.937	0.969	0.957
**P39H**$^+$	0.905	0.980	0.937	**P**$^-$**3Li**$^+$	0.938	0.974	0.961
**P79H**$^+$	0.922	0.995	0.953	**P**$^-$**7Li**$^+$	0.936	0.980	0.961
				**P**$^-$**9Li**$^+$	0.932	0.981	0.958

Table S5 Relative enthalpies and relative Gibbs energies (ΔH_{298} and ΔG_{298} in kJ mol^{-1}, referring to the most stable neutral **P9**, protonated **P19H**$^+$**/P91H**$^+$, lithiated neutral **P739Li**$^+$, lithiated monoanion **P**$^-$**39Li**$^+$, sodiated neutral **P739Na**$^+$, and sodiated monoanion **P**$^-$**39Na**$^+$, respectively) for isomers of purine derivatives calculated at the B3LYP/6-311+G(d,p) level

Isomer	ΔH_{298}	ΔG_{298}	Isomer	ΔH_{298}	ΔG_{298}	Isomer	ΔH_{298}	ΔG_{298}
**P1**	54.7[a]	54.6[a]	**P139Li**$^+$	30.4	29.6	**P139Na**$^+$	32.1	31.7
**P2**	179.0[a]	177.4[a]	**P17Li**$^+$	70.0	68.3	**P17Na**$^+$	77.2	74.8
**P3**	41.5[a]	41.6[a]	**P31Li**$^+$	115.7	113.7	**P31Na**$^+$	112.8	110.2
**P4**	221.1[a]	219.5[a]	**P37Li**$^+$	55.1	53.4	**P37Na**$^+$	61.0	58.7
**P5**	194.2[a]	192.6[a]	**P39Li**$^+$	83.7	81.7	**P39Na**$^+$	87.8	85.0
**P6**	188.2[a]	185.5[a]	**P71Li**$^+$	59.8	57.7	**P71Na**$^+$	60.5	57.9
**P7**	16.1[a]	15.8[a]	**P739Li**$^+$	**0.0**	**0.0**	**P739Na**$^+$	**0.0**	**0.0**
**P8**	162.3[a]	161.0[a]	**P91Li**$^+$	38.1	36.4	**P91Na**$^+$	38.8	36.5
**P9**	**0.0**[a]	**0.0**[a]	**P93Li**$^+$	72.8	70.0	**P93Na**$^+$	70.5	67.2
**P13H**$^+$	92.5	92.1	**P97Li**$^+$	48.0	46.3	**P97Na**$^+$	48.3	45.9
**P17H**$^+$	20.5	20.1	**P**$^-$**1Li**$^+$	112.7	108.6	**P**$^-$**1Na**$^+$	106.9	101.8
**P19H**$^+$	**0.0**	**0.0**	**P**$^-$**39Li**$^+$	**0.0**	**0.0**	**P**$^-$**39Na**$^+$	**0.0**	**0.0**
**P37H**$^+$	13.3	12.9	**P**$^-$**7Li**$^+$	75.1	71.7	**P**$^-$**7Na**$^+$	73.8	69.0
**P39H**$^+$	44.6	43.8	**P**$^-$**123456Li**$^+$	115.2	115.6			
**P79H**$^+$	27.6	27.3	**P**$^-$**56789Li**$^+$	87.2	87.7			

[a] Taken from refs [46,47].

Table S6 Comparison of the relative electronic energies for isomers of purine derivatives calculated in aqueous solution at the PCM(water)//B3LYP/6-311+G(d,p)} level with those found in the gas phase at the B3LYP/6-311+G(d,p) level (ΔE_o in kJ mol^{-1}, referring to the most stable neutral **P9**, protonated **P19H**$^+$/**P91H**$^+$, lithiated neutral **P79Li**$^+$, and lithiated monoanion **P**$^-$**9Li**$^+$ in aqueous solution, and to the most stable neutral **P9**, protonated **P19H**$^+$/**P91H**$^+$, lithiated neutral **P739Li**$^+$, lithiated monoanion **P**$^-$**39Li**$^+$ in the gas phase, respectively)

Isomer	ΔE_0(water)	ΔE_0(gas)	Isomer	ΔE_0(water)	ΔE_0(gas)
**P1**	18.5[a]	54.8[a]	**P19Li**$^+$	12.8	b
**P2**	203.0[a]	184.2[a]	**P31Li**$^+$	32.2	116.6
**P3**	27.4[a]	41.3[a]	**P37Li**$^+$	23.6	55.0
**P4**	239.6[a]	226.7[a]	**P39Li**$^+$	25.8	84.6
**P5**	207.4[a]	199.6[a]	**P71Li**$^+$	2.1	61.0
**P6**	211.4[a]	194.1[a]	**P739Li**$^+$	17.4	**0.0**
**P7**	1.0[a]	16.6[a]	**P73Li**$^+$	3.5	b
**P8**	186.4[a]	167.8[a]	**P79Li**$^+$	**0.0**	b
**P9**	**0.0**[a]	**0.0**[a]	**P91Li**$^+$	0.8	38.6
**P13H**$^+$	50.8	93.2	**P93Li**$^+$	7.2	74.5
**P17H**$^+$	2.3	21.1	**P97Li**$^+$	0.8	48.5
**P19H**$^+$	**0.0**	**0.0**	**P**$^-$**1Li**$^+$	12.3	114.4
**P37H**$^+$	13.2	13.6	**P**$^-$**3Li**$^+$	13.2	b
**P39H**$^+$	26.4	46.4	**P**$^-$**7Li**$^+$	6.7	76.2
**P79H**$^+$	10.7	28.4	**P**$^-$**9Li**$^+$	**0.0**	b
**P139Li**$^+$	c	29.9	**P**$^-$**39Li**$^+$	c	**0.0**
**P13Li**$^+$	24.2	b	**P**$^-$**123456Li**$^+$	d	117.1
**P17Li**$^+$	14.8	70.1	**P**$^-$**56789Li**$^+$	d	88.5

[a] Taken from refs [47]. [b] Monodentate adduct not found in the gas phase. [c] Bidentate adduct not found in aqueous solution. [d] π-Adduct not found in aqueous solution.

Table S7 Differences in HOMEDs {ΔHOMED = HOMED(**P**) – HOMED(**P**$^-$)} when proceeding from deprotonated to neutral purine, and proton basicities (PA and GB, respectively, in kJ mol^{-1}) for individual N and C sites in purine monoanion calculated at the B3LYP/6-311+G(d,p) level

Protonation reaction	Site of protonation	ΔHOMED5	ΔHOMED6	ΔHOMED10	PA	GB
**P**$^-$ + H$^+$ → **P9**	N9	–0.054	0.032	–0.026	**1389.4**	**1358.2**
**P**$^-$ + H$^+$ → **P7**	N7	–0.048	0.028	–0.024	1373.3	1342.3
**P**$^-$ + H$^+$ → **P3**	N3	–0.029	–0.047	–0.042	1347.9	1316.6
**P**$^-$ + H$^+$ → **P1**	N1	–0.038	–0.111	–0.064	1334.8	1303.5
**P**$^-$ + H$^+$ → **P8**	C8	–0.548	–0.266	–0.432	1227.2	1197.2
**P**$^-$ + H$^+$ → **P2**	C2	–0.319	–0.581	–0.478	1210.4	1180.7
**P**$^-$ + H$^+$ → **P6**	C6	–0.373	–0.639	–0.527	1201.2	1172.7
**P**$^-$ + H$^+$ → **P5**	C5	–0.516	–0.523	–0.526	1195.2	1165.6
**P**$^-$ + H$^+$ → **P4**	C4	–0.582	–0.533	–0.579	1168.4	1138.7

Table S8 Differences in HOMEDs {ΔHOMED = HOMED(**PH**$^+$) – HOMED(**P**)} when proceeding from neutral to protonated purine, and proton basicities (PA and GB, respectively, in kJ mol^{-1}) for individual N atoms in neutral NH tautomers calculated at the B3LYP/6-311+G(d,p) level

Protonation reaction	Site of protonation	ΔHOMED5	ΔHOMED6	ΔHOMED10	PA	GB
**P9** + H$^+$ → **P91H**$^+$	N1	0.009	–0.051	–0.018	**925.5**	**893.3**
**P9** + H$^+$ → **P93H**$^+$	N3	–0.018	–0.035	–0.030	880.9	849.4
**P9** + H$^+$ → **P97H**$^+$	N7	0.005	–0.003	–0.002	897.9	866.0
**P7** + H$^+$ → **P71H**$^+$	N1	0.033	–0.069	–0.010	921.2	889.0
**P7** + H$^+$ → **P73H**$^+$	N3	0.063	–0.018	0.024	928.4	896.2
**P7** + H$^+$ → **P79H**$^+$	N9	–0.001	0.001	–0.004	914.0	881.9
**P3** + H$^+$ → **P31H**$^+$	N1	–0.083	–0.072	–0.066	874.5	842.8
**P3** + H$^+$ → **P37H**$^+$	N7	0.044	0.057	0.042	953.7	921.9
**P3** + H$^+$ → **P39H**$^+$	N9	–0.043	0.044	–0.014	922.4	891.0
**P1** + H$^+$ → **P13H**$^+$	N3	–0.074	–0.008	–0.044	887.7	855.9
**P1** + H$^+$ → **P17H**$^+$	N7	0.023	0.070	0.030	959.7	927.8
**P1** + H$^+$ → **P19H**$^+$	N9	–0.007	0.092	0.020	980.2	947.9

Table S9 Differences in HOMEDs {ΔHOMED = HOMED(**P**$^-$**Li**$^+$) – HOMED(**P**$^-$)} when proceeding from deprotonated purine to lithiated purine monoanion, and lithium-cation basicities (LiCA and LiCB, respectively, in kJ mol^{-1}) for N atoms in monoanion calculated at the B3LYP/6-311+G(d,p) level

Metalation reaction	Site of complexation	ΔHOMED5	ΔHOMED6	ΔHOMED10	LiCA	LiCB
**P**$^-$ + Li$^+$ → **P**$^-$**1Li**$^+$	N1	–0.011	–0.062	–0.031	504.8	475.9
**P**$^-$ + Li$^+$ → **P**$^-$**39Li**$^+$	N3, N9	0.002	0.026	0.003	**617.5**	**584.5**
**P**$^-$ + Li$^+$ → **P**$^-$**7Li**$^+$	N7	–0.017	0.023	–0.016	542.4	512.8
**P**$^-$ + Li$^+$ → **P**$^-$**123456Li**$^+$	pi(-)	–0.018	–0.04	–0.018	502.4	468.9
**P**$^-$ + Li$^+$ → **P**$^-$**56789Li**$^+$	pi(-)	–0.024	0.004	–0.018	530.4	496.8

Table S10 Differences in HOMEDs {ΔHOMED = HOMED(**PLi**$^+$) – HOMED(**P**)} when proceeding from neutral to lithiated purine, and lithium-cation basicities (LiCA and LiCB, respectively, in kJ mol^{-1}) for N atoms in neutral NH tautomers calculated at the B3LYP/6-311+G(d,p) level

Metalation reaction	Site of complexation	ΔHOMED5	ΔHOMED6	ΔHOMED10	LiCA	LiCB
**P9** + Li$^+$ → **P91Li**$^+$	N1	0.016	–0.025	–0.004	188.7	157.8
**P9** + Li$^+$ → **P93Li**$^+$	N3	–0.010	–0.015	–0.014	154.1	124.3
**P9** + Li$^+$ → **P97Li**$^+$	N7	0.006	–0.001	0.001	178.8	148.0
**P7** + Li$^+$ → **P71Li**$^+$	N1	0.026	–0.033	0.000	183.2	152.4
**P7** + Li$^+$ → **P739Li**$^+$	N3, N9	0.031	0.009	0.019	**243.0**	**210.1**
**P3** + Li$^+$ → **P31Li**$^+$	N1	–0.038	–0.034	–0.030	152.7	122.2
**P3** + Li$^+$ → **P37Li**$^+$	N7	0.035	0.040	0.033	213.3	182.4
**P3** + Li$^+$ → **P39Li**$^+$	N9	–0.036	0.027	–0.015	184.6	154.2
**P1** + Li$^+$ → **P139Li**$^+$	N3, N9	0.019	0.067	0.026	251.2	219.3
**P1** + Li$^+$ → **P17Li**$^+$	N7	0.019	0.047	0.023	211.5	180.6

Table S11 Differences in the HOMED values {ΔHOMED = HOMED(**P**$^-$**Na**$^+$) – HOMED(**P**$^-$)} when proceeding from deprotonated purine to sodiated purine monoanion, and sodium-cation basicities (NaCA and NaCB, respectively, in kJ mol^{-1}) for N atoms in monoanion calculated at the B3LYP/6-311+G(d,p) level

Metalation reaction	Site of complexation	ΔHOMED5	ΔHOMED6	ΔHOMED10	NaCA	NaCB
**P**$^-$ + Na$^+$ → **P**$^-$**1Na**$^+$	N1	–0.005	–0.045	–0.020	422.3	394.9
**P**$^-$ + Na$^+$ → **P**$^-$**39Na**$^+$	N3, N9	0.001	0.021	0.002	**529.2**	**496.8**
**P**$^-$ + Na$^+$ → **P**$^-$**7Na**$^+$	N7	–0.015	0.019	–0.006	455.4	427.7

Table S12 Differences in HOMEDs {ΔHOMED = HOMED(**PNa**$^+$) – HOMED(**P**)} when proceeding from neutral to sodiated purine, and sodium-cation basicities (NaCA and NaCB, respectively, in kJ mol^{-1}) for N atoms in neutral NH tautomers calculated at the B3LYP/6-311+G(d,p) level

Metalation reaction	Site of complexation	ΔHOMED5	ΔHOMED6	ΔHOMED10	NaCA	NaCB
**P9** + Na$^+$ → **P91Na**$^+$	N1	0.016	–0.018	0.000	131.3	101.9
**P9** + Na$^+$ → **P93Na**$^+$	N3	–0.009	–0.009	–0.009	99.6	71.3
**P9** + Na$^+$ → **P97Na**$^+$	N7	0.013	–0.001	0.005	121.8	92.5
**P7** + Na$^+$ → **P71Na**$^+$	N1	0.021	–0.023	0.002	125.7	96.4
**P7** + Na$^+$ → **P739Na**$^+$	N3, N9	0.030	0.008	0.019	**186.2**	**154.3**
**P3** + Na$^+$ → **P31Na**$^+$	N1	–0.029	–0.025	–0.022	98.8	69.8
**P3** + Na$^+$ → **P37Na**$^+$	N7	0.036	0.035	0.032	150.7	121.3
**P3** + Na$^+$ → **P39Na**$^+$	N9	–0.019	0.023	–0.007	123.9	94.9
**P1** + Na$^+$ → **P139Na**$^+$	N3, N9	0.015	0.056	0.022	192.7	161.4
**P1** + Na$^+$ → **P17Na**$^+$	N7	0.022	0.038	0.023	147.6	118.2

Table S13 Gas-phase metal-cation basicities (LiCA, LiCB, NaCA, and NaCB at 298 K in kJ mol-1) for the monodentate M^+-adduct formed with the imidazole monoanion

Compound	Method	LiCA	LiCB	NaCA	NaCB
**Im⁻**	DFT	607.1	578.0	512.7	485.4
	G2	593.8	565.6	497.1	473.2
	G2MP2	593.6	565.4	497.1	473.2
	G3	601.9	573.7	514.3	490.4
	G3B3	602.1	574.6	516.8	484.4

a) NH tautomers

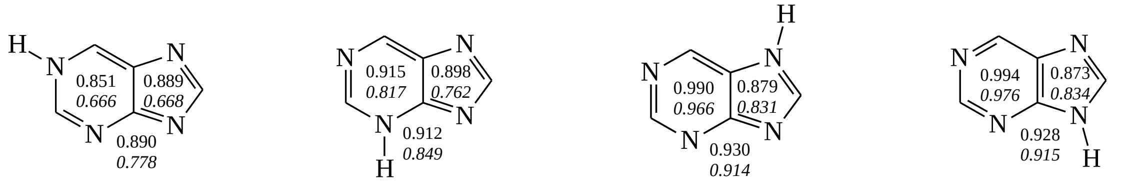

N1H (**P1** <0.01%) N3H (**P3** <0.01%) N7H (**P7** 0.2%) N9H (**P9** 99.8%)

b) CH tautomers

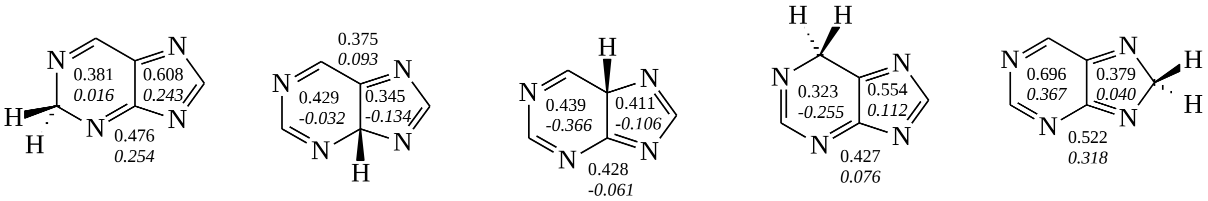

C2H (**P2** <0.01%) C4H (**P4** <0.01%) C5H (**P5** <0.01%) C6H (**P6** <0.01%) C8H (**P8** <0.01%)

Figure S1 Nine prototropic tautomers of neutral purine, four NH tautomers (a) and five CH tautomers (b), their percentage contents (placed in parentheses near abbreviation **P*i*** for tautomer), and geometry-based indices (HOMED in normal and rHOMA in italic): HOMED5 and rHOMA5 for the imidazole fragment (included in the 5-membered ring), HOMED6 and rHOMA6 for the pyrimidine part (included in the 6-membered ring), and HOMED10 and rHOMA10 for the entire purine system (placed near formula). Geometries optimized and percentage contents calculated for isolated tautomers at the B3LYP/6-311+G(d,p) level. Data taken from ref [47]

a) Monoanion isomer

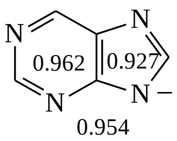

P$^-$ (100%)

b) Monocation isomers

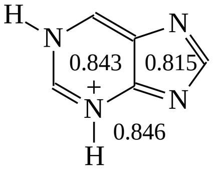

N1HN3H$^+$
(**P13H$^+$/P31H$^+$** <0.01)

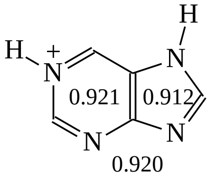

N1HN7H$^+$
(**P17H$^+$P71H$^+$** 0.03%)

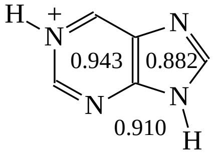

N1HN9H$^+$
(**P19H$^+$/P91H$^+$** 99.4%)

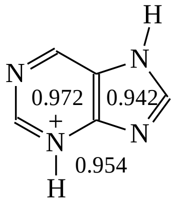

N3HN7H$^+$
(**P37H$^+$/P73H$^+$** 0.5%)

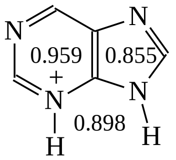

N3HN9H$^+$
(**P39H$^+$/P93H$^+$** <0.01%)

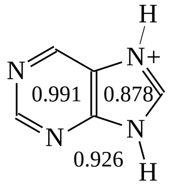

N7HN9H$^+$
(**P79H$^+$/P97H$^+$** 0.02%)

Figure S2 Monodeprotonated (a) and monoprotonated forms of purine NH tautomers (b), their percentage contents (given in parentheses), HOMED5, HOMED6 (included in the imidazole and pyrimidine rings, respectively), and HOMED10 (placed near formula). Geometries optimized and percentage contents calculated for isolated tautomers at the B3LYP/6-311+G(d,p) level

a) Lithiated monoanion isomers

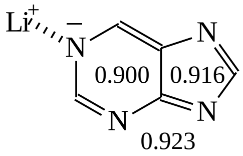

P$^-$1Li$^+$ (<0.01%)

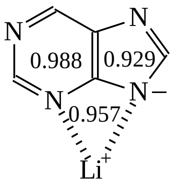

P$^-$39Li$^+$ (100%)

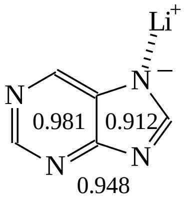

P$^-$7Li$^+$ (<0.01%)

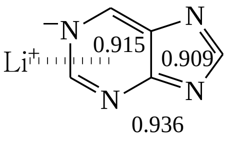

P$^-$123456Li$^+$ (<0.01%)

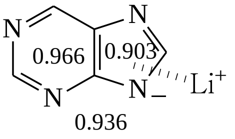

P$^-$56789Li$^+$ (<0.01%)

b) Lithiated purine NH tautomers

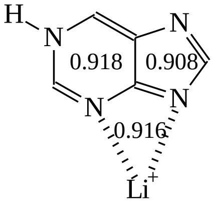

N1HN39Li$^+$
(**P139Li$^+$** <0.01%)

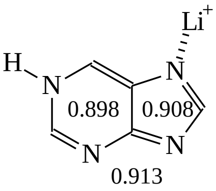

N1HN7Li$^+$
(**P17Li$^+$** <0.01%)

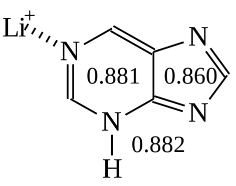

N3HN1Li$^+$
(**P31Li$^+$** <0.01%)

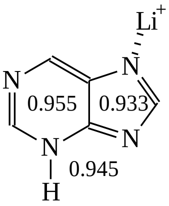

N3HN7Li$^+$
(**P37Li$^+$** <0.01%)

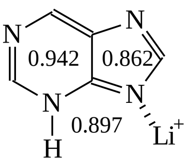

N3HN9Li$^+$
(**P39Li$^+$** <0.01%)

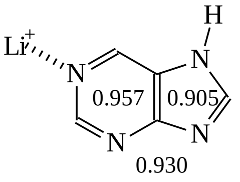

N7HN1Li$^+$
(**P71Li$^+$** <0.01%)

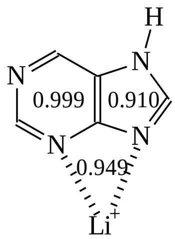

N7HN39Li$^+$
(**P739Li$^+$** 100%)

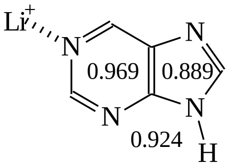

N9HN1Li$^+$
(**P91Li$^+$** <0.01%)

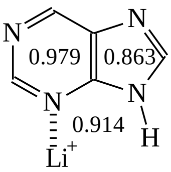

N9HN3Li$^+$
(**P93Li$^+$** <0.01%)

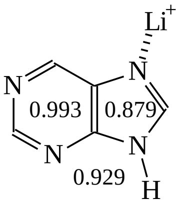

N9HN7Li$^+$
(**P97Li$^+$** <0.01%)

Figure S3 Lithiated isomers of purine monoanion (a) and of its neutral NH tautomers (b), their percentage contents (given in parentheses), HOMED5, HOMED6 (included in the imidazole and pyrimidine rings, respectively), and HOMED10 (placed near structure). Geometries optimized and percentage contents calculated for isolated isomers at the B3LYP/6-311+G(d,p) level

a) Sodiated monoanion isomers

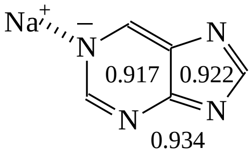

P⁻1Li+ (<0.01%)

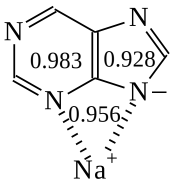

P⁻39Li+ (100%)

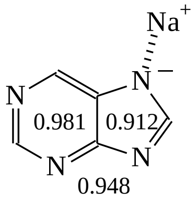

P⁻7Li+ (<0.01%)

b) Sodiated purine NH tautomers

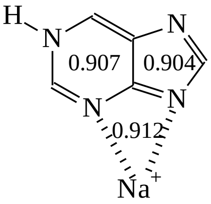

N1HN39Na+
(**P139Na**+ <0.01%)

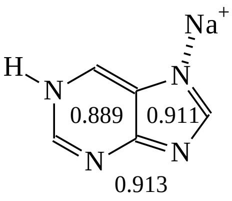

N1HN7Na+
(**P17Na**+ <0.01%)

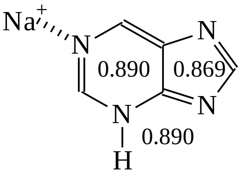

N3HN1Na+
(**P31Na**+ <0.01%)

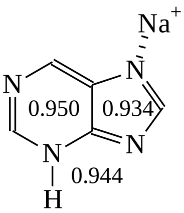

N3HN7Na+
(**P37Na**+ <0.01%)

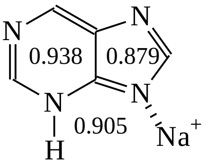

N3HN9Na+
(**P39Na**+ <0.01%)

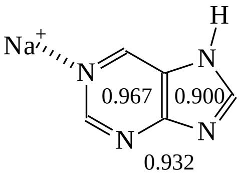

N7HN1Na+
(**P71Na**+ <0.01%)

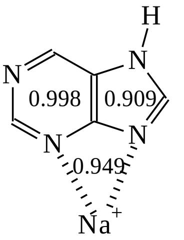

N7HN39Na+
(**P739Na**+ 100.0%)

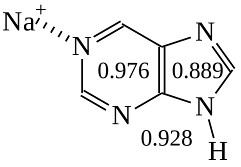

N9HN1Na+
(**P91Na**+ <0.01%)

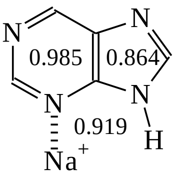

N9HN3Na+
(**P93Na**+ <0.01%)

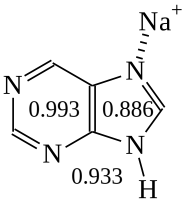

N9HN7Na+
(**P97Na**+ <0.01%)

Figure S4 Sodiated isomers of purine monoanion (a) and of its neutral NH tautomers (b), their percentage contents (given in parentheses), HOMED5, HOMED6 (included in the imidazole and pyrimidine rings, respectively), and HOMED10 (placed near structure). Geometries optimized and percentage contents calculated for isolated isomers at the B3LYP/6-311+G(d,p) level

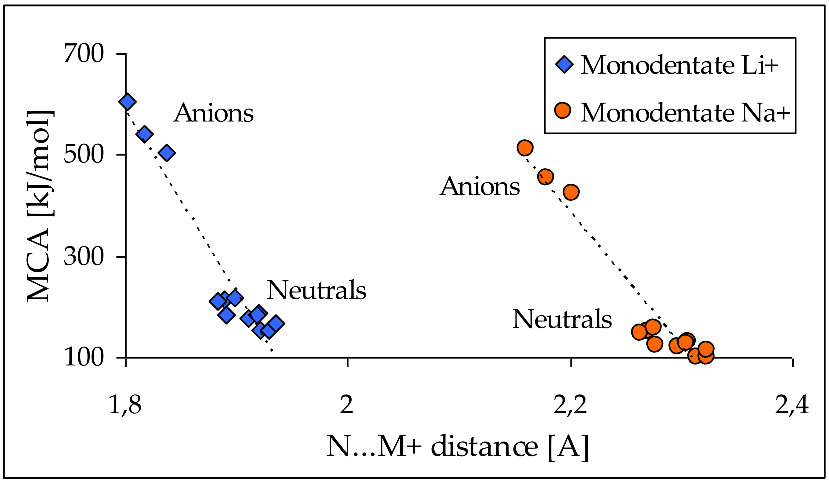

Figure S5 Linear trends between N...M$^+$ distances (in Å) and MCA (in kJ mol^{-1}) for monodentate adducts of neutral and deprotonated forms of purine and its structural building blocks found at the B3LYP/6-311+G(d,p) level

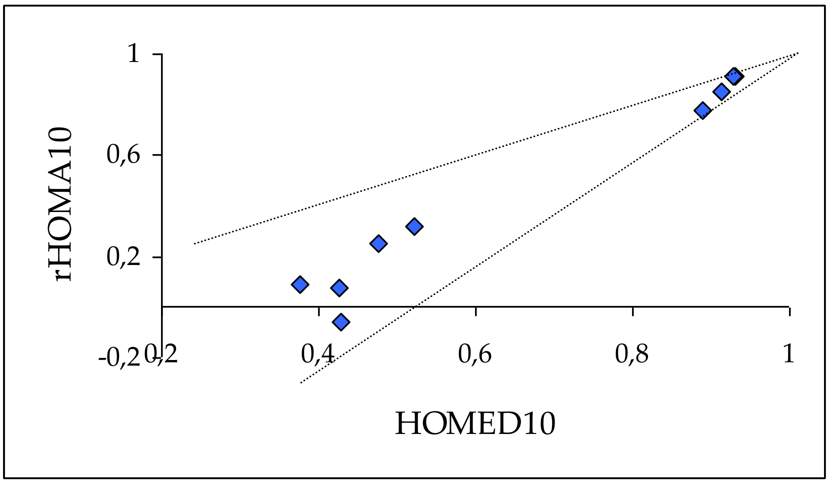

Figure S6 Lack of correlation between rHOMA and HOMED for all nine neutral purine tautomers. Data taken from ref [47]. Dotted lines correspond to the linear relationships between the geometry-based indices estimated for compounds containing only CN bonds (top line), and only CC bonds (lower line) at the same level of theory {B3LYP/6-311+G(d,p)} as for purine. Lines taken from ref [68]

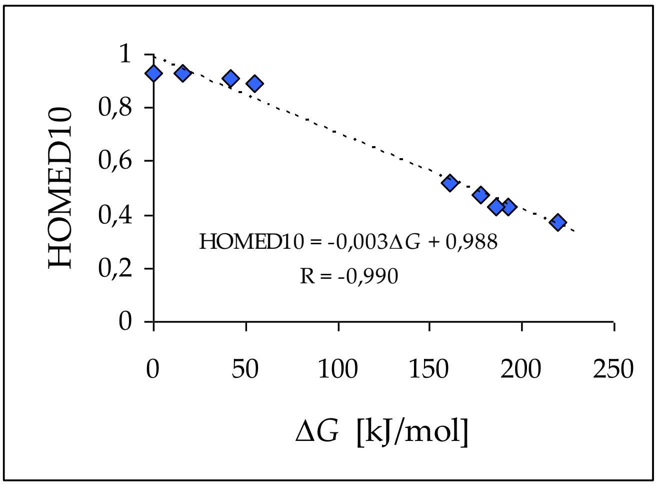

Figure S7 Linear trend between the HOMED indices and relative Gibbs energies (ΔG) estimated for all nine possible neutral purine tautomers in the gas phase. DFT data taken from refs [46,47]

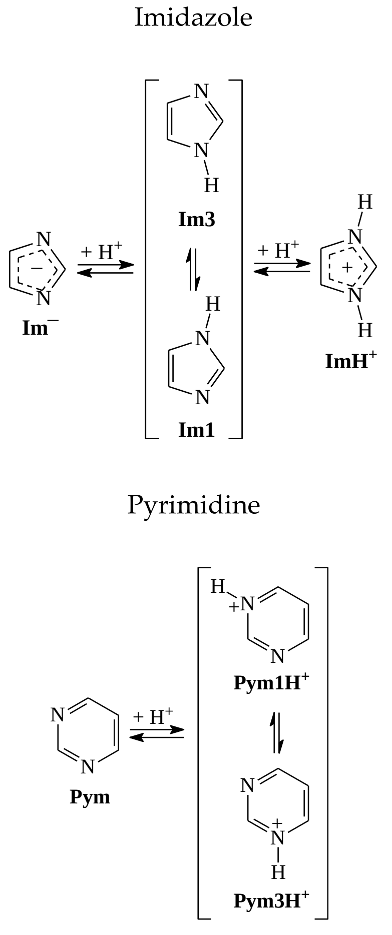

Scheme S1 Deprotonation/protonation reactions for purine building blocks

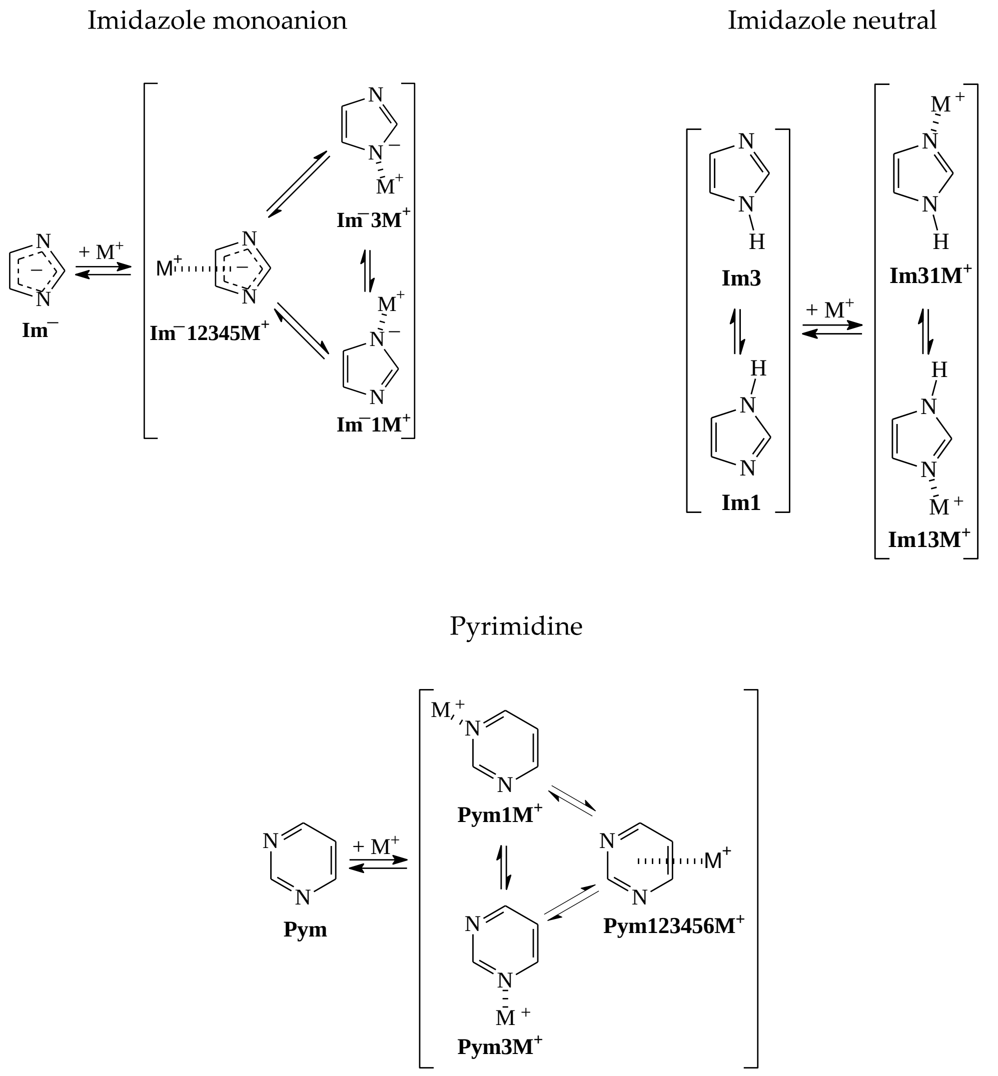

Scheme S2 Cationization reactions for purine building blocks

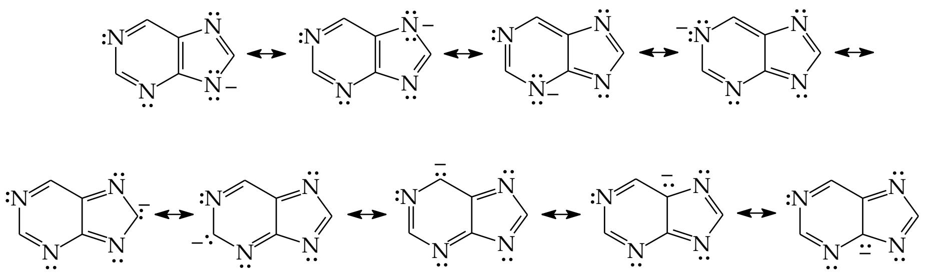

Scheme S3 Resonance structures for the purine monoanion (**P**⁻) explain nine conjugated sites that can attach a proton leading to the tautomeric mixture consisting of nine neutral tautomers (**P1**-**P9**)
